# IAVCP (Influenza A Virus Consensus and Phylogeny): Automatic Identification of the Genomic Sequence of the Influenza A Virus from High-Throughput Sequencing Data

**DOI:** 10.3390/v16060873

**Published:** 2024-05-29

**Authors:** Anastasiia Iu. Paremskaia, Pavel Yu. Volchkov, Andrei A. Deviatkin

**Affiliations:** 1 Federal Research Center for Innovator and Emerging Biomedical and Pharmaceutical Technologies, 125315 Moscow, Russia; volchkov@genlab.llc; 2 Department of Fundamental Medicine, Lomonosov Moscow State University, 119992 Moscow, Russia; 3 The MCSC Named after A. S. Loginov, 111123 Moscow, Russia; 4 Faculty of Bioengineering and Bioinformatics, Lomonosov Moscow State University, 119992 Moscow, Russia

**Keywords:** IAV, HTS (high-throughput sequencing), reassortment, phylogenetic, consensus

## Abstract

Recently, high-throughput sequencing of influenza A viruses has become a routine test. It should be noted that the extremely high diversity of the influenza A virus complicates the task of determining the sequences of all eight genome segments. For a fast and accurate analysis, it is necessary to select the most suitable reference for each segment. At the same time, there is no standardized method in the field of decoding sequencing results that allows the user to update the sequence databases to which the reads obtained by virus sequencing are compared. The IAVCP (influenza A virus consensus and phylogeny) was developed with the goal of automatically analyzing high-throughput sequencing data of influenza A viruses. Its goals include the extraction of a consensus genome directly from paired raw reads. In addition, the pipeline enables the identification of potential reassortment events in the evolutionary history of the virus of interest by analyzing the topological structure of phylogenetic trees that are automatically reconstructed.

## 1. Introduction

The influenza A virus (IAV) genome, approximately 13.5 kilobases (kb) in size, is composed of eight segments of single-stranded negative-sense RNA and encodes 17 proteins [1]. The first three segments, polymerase basic 2 (PB2), polymerase basic 1 (PB1), and polymerase acidic (PA), encode the subunits of the viral polymerase. The PA segment can also express truncated forms of the PA protein, PA-X, PA-N155, and PA-N182. Some strains also harbor an additional open reading frame in PB1, leading to the expression of the PB1-F2 protein, which enhances viral pathogenicity. A truncated N-terminal form, PB1-N40, negatively impacts virus replication by reducing polymerase enzymatic activity. The fourth and sixth segments encode surface glycoproteins—hemagglutinin (HA) and neuraminidase (NA). The fifth highly conserved segment encodes the nucleoprotein (NP), which actively participates in nuclear transport, translation, and virus replication. The seventh segment, the matrix (M) gene, encodes three proteins: M1, M2, and M42. The eighth segment, the nonstructural (NS) gene, encodes proteins NS1, NS2, and NS3 involved in suppressing the host cell’s antiviral response. In the comprehensive review by authors Chauhan and Gordon, the functions of the IAV genome segments are thoroughly examined, with descriptions of some significant mutations [2].

The genome of the IAV is highly variable, and according to some estimates, the mutation rate falls within the range of 10^−6^ to 10^−3^ substitutions per site per replication [3,4]. However, the mutation rate varies among segments, traditionally drawing the most attention to segments encoding the surface glycoproteins hemagglutinin (HA) and neuraminidase (NA), responsible for viral binding and release, respectively. These proteins also exhibit a close functional relationship, with NA capable of compensating for reduced HA affinity by increasing enzymatic activity [5,6]. The virus polymerase’s limited proofreading ability contributes to mutation accumulation [7]. This process leads to changes in the virus’s antigenic properties, enabling it to evade detection and destruction by the host’s immune system. The gradual accumulation of mutations altering antigenic specificity is termed antigenic drift.

Another aspect contributing to the high variability of IAV is antigenic shift. This is a more radical change in the HA or NA segments of the virus resulting from reassortment during the coinfection of a single host cell by two viruses [8]. Additionally, the reassortment process can involve the exchange of any of the IAV segments. Under such conditions, the assembly of a new virion can occur from all available segment variants. Consequently, viral genes undergo reassortment, potentially leading to the emergence of novel hybrid subtypes of influenza virus with a combination of genes from both parental subtypes.

Each segment of the IAV genome contains one or more genes flanked by 3′ and 5′ untranslated regions (UTRs). The UTR consists of a conservative motif and a segment-specific noncoding region, which varies between 5 and 45 nucleotides [9,10]. The identification of specific noncoding regions forms the basis of the selective genome assembly model [11], which ensures the packaging of all eight virus segments and its ability to replicate. The correct assembly of the influenza virus genome is determined by both the untranslated regions carrying packaging signals for each segment and the coding part. In particular, the conservative regions of the NP gene have been shown to play a crucial role in genome packaging [12]. Incompatibility between polymerase subunits and packaging signals limits the ability to reassort [13,14].

Nevertheless, viruses that have undergone reassortment can be extremely dangerous, as the pandemic caused by the H1N1 virus in 2009 showed. Multiple reassortments resulted in the pandemic virus containing genetic material from H1N1 avian influenza, H1N1 swine influenza, and H3N2 human seasonal influenza viruses [15]. Reassortment is thus an important evolutionary mechanism that enables viruses with segmented genomes to rapidly change their phenotypic properties.

In early studies, reassortment events were detected by direct comparisons of sequences of two viruses or individual segments [16]. Other studies use phylogenetic tree reconstruction approaches that compare topology [17] or mutation patterns [18,19,20]. The evolutionary history may be different for each segment of the influenza virus, and the number of sequences that can be included in such analyses remains extremely large.

High-throughput sequencing (HTS) technologies have become a powerful method for massive parallel sequencing of amplicons and analysis of viral genomes. Bioinformatic approaches for analyzing HTS results include de novo assembly, mapping of raw data to the reference genome, and detection of viral sequences in assembled metagenomics data. When a virus with a completely unknown genome, i.e., there is nothing to compare it to, a de novo assembly is performed. The assembly algorithms differ depending on whether long or short reads were obtained. Short-read sequencing involves generating DNA or cDNA sequences that are typically a few hundred base pairs in length. Short-read sequencing platforms like Illumina and MGI are widely utilized in genomics research and clinical applications due to their high throughput and cost-effectiveness. Long-read sequencing technologies, such as PacBio and Oxford Nanopore, afford the ability to generate extended DNA or RNA sequences spanning thousands of base pairs. This facilitates genome assembly, detection of structural variants, transcriptome exploration, and analysis of nucleotide modifications [21]. For the assembly of short reads, Hamiltonian and Eulerian de Bruijn graphs [22] are used, while for the assembly of long reads obtained with third-generation sequencers, the overlap–layout consensus (OLC) [23] algorithm is used. This approach poses numerous challenges, as described in the review paper [24].

Currently, numerous tools have been developed for the de novo assembly of viral genomes, including from metagenomic data: Metaviral SPAdes [25], SAVAGE [26], PEHaplo [27], MEGAHIT [28], VICUNA [29], and IVA [30].

Generally, such approaches are resource-intensive and are used to discover new, previously unknown viruses [24]. Thus, when the structure, approximate length, and sequence of the virus are known, de novo assembly is not applied [31,32,33].

Aligning to reference genomes significantly reduces the required computing resources and accelerates the speed of the experiments. Specialized tools have been developed for processing viral sequences. Examples include iVar [34], ViralConsensus [35], INSaFLU [36], and IRMA [37]. iVar, which counts the number of substitutions compared to the reference and determines a consensus sequence, is the method of choice for analyzing SARS-CoV-2. However, it is worth noting that this was made possible due to the low mutation rates in SARS-CoV-2 genome replication and the use of the Wuhan virus sequence as a reference.

In contrast to SARS-CoV-2, the mutation frequency of the influenza A virus (IAV) is significantly higher according to recent studies, which poses difficulties in analyzing data obtained from sequencing [38]. Indeed, IAVs have been circulating in wildlife for ages, leading to the accumulation of a vast diversity of strains. For example, NC_045512.2 (SARS-CoV-2 collected in China in December 2019) and PP179876 (SARS-CoV-2 collected in the USA in December 2023) according to BLASTn are identical in 29500 out of 29669 nucleotides (99.43%), whereas CY103892 (genomic sequence of the fourth segment of A/little yellow-shouldered bat/Guatemala/060/2010(H17N10) IAV strain) and HQ020375 (genomic sequence of the fourth segment of A/great cormorant/Qinghai/1/2009(H5N1) IAV strain) share 425 out of 633 nucleotides (67.14%) on less than a half of the fourth segment sequence, while the other part of this segment has no homology. Therefore, the availability of a representative reference sequence is a crucial prerequisite for this read-processing approach, especially for viral phylogenetic studies.

Here, we present the IAVCP pipeline, which implements flexible analysis of the segmented IAV genome, obtaining a representative consensus from raw reads, and a straightforward method for detecting possible reassortment through phylogenetic tree construction. This pipeline is freely available at https://github.com/ana-way/IAVCP_workflow (accessed on 27 May 2024).

## 2. Materials and Methods

### 2.1. Materials

All sequences listed in GenBank as representatives of the IAV species (txid11320) as of January 2023 were downloaded from GenBank (n = 872,467) in GenBank format. The GenBank file was converted to a FASTA file with the sequence segment number information stored in the FASTA header. Based on this information, the resulting set of sequences was divided into eight groups, corresponding to the number of segments in the IAV. In the next step, incomplete sequences were removed from the datasets according to the sequence length. To remove nearly identical sequences with more than 99.8% identical nucleotides, the software CD-HIT [39] was used. Finally, the resulting FASTA files for each segment were processed to exclude GeneBank entries that were erroneously annotated. For instance, the sequence of segment PB1 was annotated as belonging to segment PB2. In total, the comprehensive database of IAV sequences (IAVCP db1) contained 13,640 data records for the first segment; 13,462 for the second segment; 14,826 for the third segment; 32,352 for the fourth segment; 14,128 for the fourth segment; 33,747 for the sixth segment; 16,321 for the seventh segment; and 26,742 for the eighth segment. In IAVCP, these sequence datasets are used to search for the most appropriate reference sequence to generate the consensus sequence of the viral target genome. In addition, we selected only those viral sequences for which the complete or near-complete sequence for each segment was known (IAVCP db2). All IAV sequences from GenBank (n = 872,467) were used for this purpose. Only sequences that could be assigned to a virus in which all segment sequences are at least 500 nucleotides long (58,564 sequences for each segment) were selected according to the strain name field. These sequence datasets can be used to determine whether the viral target genome shows signs of reassortment. It should be noted that these datasets (either for consensus generation or reassortment analysis) are preset, but they can be modified to be generated from the user datasets. For example, the GISAID database contains information on viral sequences that is not available in the GenBank database. At the same time, this information cannot be made publicly available due to usage restrictions [40].

Additionally, publicly available data from the Sequence Read Archive (SRA) https://www.ncbi.nlm.nih.gov/sra/ (accessed on 27 May 2024) were used to test our pipeline, which will be further elaborated upon in Section 3.

### 2.2. Methods

The IAVCP pipeline, which operates on the Linux operating system and is built using the Snakemake tool [41], provides an efficient means for automating the analysis of influenza A virus sequencing data. Snakemake offers a declarative approach to describing the workflow, supports parallel execution, and allows for pipeline parameter customization, making it flexible and adaptable for analyzing large volumes of data. Detailed instructions on how to use the tool and its source code are available in the README file in the GitHub repository (https://github.com/ana-way/IAVCP_workflow (accessed on 27 May 2024)).

## 3. Results

**Figure 1:**
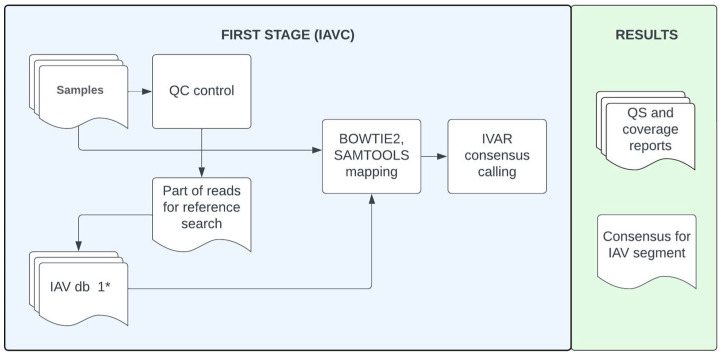
The IAVC workflow enables the generation of a consensus sequence from paired reads for each segment of the virus of interest. All steps except quality control are performed independently for each segment. * IAV db 1 represents a database of all available IAV sequences from GenBank.

**Figure 2:**
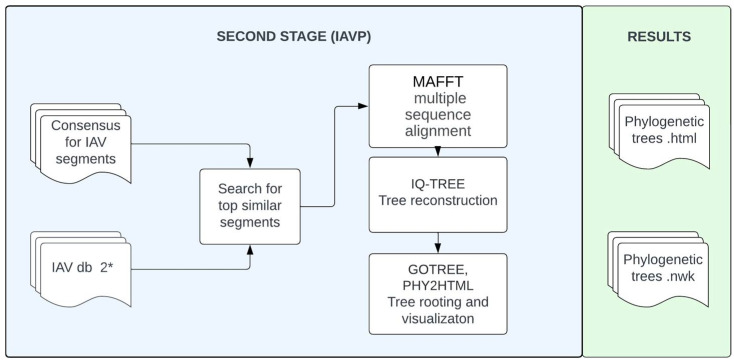
IAVP workflow for the detection of reassortment events. * IAV db 2 is a database with all complete IAV genomes from GenBank.

Two stages can be conditionally distinguished in the IAVCP pipeline: IAVC (IAV consensus)—to determine the consensus sequence from the HTS results, and IAVP (IAV phylogeny)—to theoretically recognize reassortment processes in the evolutionary history of the virus of interest. The workflows are shown schematically in Figure 1 and Figure 2. To use the pipeline, the Python interpreter should be installed together with the Conda or Mamba package manager. This ensures simple and efficient management of the environments and installation of the necessary dependencies for IAVCP. The pipeline is available for download from the GitHub repository (https://github.com/ana-way/IAVCP_workflow (accessed on 27 May 2024)).

In addition to the executable files, the mandatory files contain the sequences of the IAV segments in FASTA format, which are automatically downloaded when the software is installed. These sequences are used to create the database required for the BLASTn search for the most suitable references for each segment.

The pipeline is designed to process paired-end reads and accepts a configuration file in .csv format as input. This file contains a common prefix that is used to name the result files, as well as relative paths to the reads that are processed in the pipeline. Quality control and read trimming are performed with the FASTP tool. It was chosen as the tool because it significantly outperforms other tools in terms of processing speed for similar tasks while maintaining comparable quality standards [42]. FASTP uses the default adapter sequences for trimming the reads. The pipeline utilizes default settings of FASTP. However, users can set the minimum quality score for trimming at the 3′ and 5′ ends. It should be noted that the minimum read length has been constrained to 20, diverging from FASTP’s default setting of 15. It should be noted that if specific, unique adapters are used in the experiments, their sequences can be added to the database of sequences used by the FASTP algorithm (as specified in the instructions for using IAVCP). The current version of the pipeline is limited to working only with short paired-end reads (generated by Illumina, Ion Torrent, BGI technology). It should be noted that the default sequences of adapters used in trimming are set for Illumina technology. In the case of using a different platform, the file available at https://github.com/ana-way/IAVCP_workflow/blob/main/data/adapters/adapters.fa (accessed on 27 May 2024) should be changed to the adapter sequences for the corresponding platform in FASTA format with keeping the name of this file.

A key feature of IAVCP is the reference search for each segment in the generated databases using the BLASTn algorithm [43]. A small portion of the reads (by default, 25,000) are compared with the comprehensive database of IAV sequences. The number of reads to be processed can be increased if the proportion of viral reads in the sequencing library is low, but it is worth considering that this increases the runtime of the pipeline. The BLASTn search is constrained by the parameters qcov_hsp_perc, e-value, and max_target_seqs. The parameter “qcov_hsp_perc” makes it possible to filter reads according to a certain proportion of nucleotides in the read that are not mapped to the reference (50 by default for the primary reference search). Setting a threshold therefore ensures sequence similarity and prevents erroneous alignments where only a small segment of the query matches a reference sequence. The parameter “e-value”, expectation value, is a measure of the statistical significance of the sequence similarity between the query sequence and the database sequences. A smaller e-value indicates a more significant match. The parameter value of 0.001 is used to filter out nonsignificant hits between reads and references. Lastly, “max_target_seqs” specifies the number of references to be found for each read and written to the BLASTn search output (by default, one for the primary reference search).

The BLASTn search returns the closest sequences from the comprehensive database for each read. The most frequently represented sequence is selected as the reference sequence for further analysis. As a result, the most similar genomic IAV sequences are selected as the most appropriate reference for each segment of the virus of interest for further analysis. BOWTIE2 [44] is then used to assign the sequencing reads to the identified reference sequences for each segment. SAMtools is used to create the alignments written in BAM format and to analyze the coverage metrics. The final processing step is to generate a consensus sequence from the aligned BAM file using the iVar tool [34]. The steps from selecting the reference sequences to creating the consensus sequence are iterated for each segment.

The resulting sequences of eight segments are used in the next step of the IAVP workflow to search for reassortment events. Each segment is used as a query against databases created from complete IAV genomes. Thus, a set of viral sequences most similar to all segment sequences of the virus of interest is used to build the tree. By default, ten similar sequences are selected for each segment. If the virus of interest has segments originating from different ancestral viruses, the maximum number of viruses whose genome segments are used for phylogenetic reconstruction is 80 (10 similar sequences for each of the 8 segments). In real cases, this number will be lower because reassortment events are limited as mentioned above. It should be noted that the IAVCP automatically identifies the set of viruses for which at least one segment sequence is similar to the corresponding segment sequence of the virus of interest. Moreover, all these sequences are used to reconstruct the evolutionary relationships between the individual segment sequences. The most plausible explanation for the different topology of the phylogenetic trees constructed for different segment sequences of the same viruses is the reassortment that has taken place between the ancestors of these viruses.

The phylogenetic analysis includes the use of the tools MAFFT [45], IQ-TREE [46], and GOTREE [47]. MAFFT was used for multiple sequence alignment. The phylogenetic tree was reconstructed for each segment by IQ-TREE with the GTR + I + G substitution model. GOTREE was used to automatically select the midpoint root of the phylogenetic tree. The principle of this method is to find a point in the tree that minimizes the sum of the lengths of all paths from this point to the final vertices of the tree. This approach makes it possible to find a hypothetical root of the tree that best fits the assumption that evolutionary changes have occurred approximately equally on both sides of this point. This method is often used for virus populations, as it is hardly possible to identify an ideal outgroup for rooting. The resulting rooted tree can be visualized with special tools or with a visualization implemented in the pipeline that leads to the creation of the phylogenetic tree in html format.

The pipeline can be customized by the user to take into account different qualities of the input data. For example, if the sample contains a small amount of viral reads, it may be insufficient to consider only the first 25,000 reads for reference selection. It is therefore advisable to increase the parameter “PART”—part of the reads to be compared with the database. The IAVCP uses a quality base cutoff of 20, which is adjustable. It is also possible to change the depth of base coverage required for consensus finding using the “CONSENSUS_DEPTH” parameter. However, it is advisable to interpret the results of low-quality reads with caution and validate them with other methods. If the part of the reads used for the reference selection does not contain any viral reads, no reference sequence is selected, thus resulting in no consensus sequence being generated. In the case of low coverage for certain regions, positions will be filled with “N”, indicating that any nucleotide is possible.

It should be noted that simultaneous infection of a host by several IAV strains is possible [48]. On the other hand, coinfection is a relatively rare event [49]. Several possibilities of coinfection should be considered for detection by analyzing sequencing data.

First, coinfection with closely related viruses that differ in a point mutation is possible. The reads from different viruses are then mapped to a reference. In our pipeline, this can be taken into account by changing the default value of the “Minimum frequency threshold” parameter [34]. This parameter defines the interpretation of reference sites for which different reads contain different nucleotides. The default value (0) means that only the most strongly represented nucleotide in this ambiguous site is taken into account. Increasing this parameter makes it possible to use several nucleotides at this position. Implementing the parameter “minimum frequency threshold” with the value 0 for a position with 83 adenosines, 15 cytidines, and 4 guanosines would result in the adenosine being fixed at this position of the consensus sequence. At the same time, the implementation of this parameter with the value 0.9 would result in the degenerate letter “M” (“A” or “T”) being fixed at this position of the consensus sequence. In other words, the appearance of the degenerate nucleotides in the consensus sequence as a result of the change in the “minimum frequency threshold” may be an indication of coinfection by closely related viruses in the sample.

Secondly, it is possible that a coinfection with different viruses has taken place. To detect such an event, we have added the MIX module. Thus, the BLAST analysis is repeated against the hemagglutinin and neuraminidase databases. The number of reads used for comparison is regulated by the “PART” parameter. As a result, BLAST outputs two tables in tab-separated-value format (.tsv), one for hemagglutinin and the other for neuraminidase. To clarify this output, two additional tables have been created in text format, each showing the number of reads similar to the datasets in the respective database. An example of this output can be found at this link: https://github.com/ana-way/IAVCP_workflow/tree/main/examples/SRR27181136_mix (accessed on 27 May 2024). In the case of SRR27181136 (https://trace.ncbi.nlm.nih.gov/Traces/?view=run_browser&acc=SRR27181136&display=metadata (accessed on 27 May 2024)), for example, the results include not only the primary type H3N2, but also H1N1. This is consistent with experimental expectations.

To demonstrate the capabilities of IAVP, we generated artificial IAV in silico for which four segments were obtained from H5N1 (A/chicken/South Kalimantan/UT6028/06) and another four segments from H3N2 (A/Tokyo/Ut-Sk-1/07) viruses. The resulting virus contained the following segments: segments 1, 3, 4, and 6 belong to H3N2 and segments 2, 5, 7, and 8 belong to H5N1. The results of the phylogenetic analysis of the construct can be found at the following link: https://github.com/ana-way/IAVCP_workflow/tree/main/examples/in_silico_reassortant (accessed on 27 May 2024). According to the topology of the trees, each segment of the construct belonged to the corresponding virus.

In addition, we tested the previously mentioned virus A/Unknown/Buryatia/Arangatui-1/2020(H13N8), which showed signs of reassortment [50]. The results of the analysis with IAVP can be accessed via the following link: https://github.com/ana-way/IAVCP_workflow/tree/main/examples/OQ868199_natural_reassortant (accessed on 27 May 2024). The trees generated for different segments show different topologies, indicating reassortment events in the evolutionary history of the selected viruses.

The pipeline is available in several execution modes. The full version (IAVCP) is aimed at assembling consensus sequences from paired reads and producing basic phylogenetic reconstructions. Phylogenetic trees are constructed based on sequences that are similar to the corresponding segments in the virus of interest. The description of the closest reference virus based on GenBank annotation including the information about the subtype is specified in the output of the IAVCP. Comparing the topology of these trees should reveal reassortment events in the evolutionary history of the selected viruses. Additionally, partial pipeline execution is possible with consensus assembly (IAVC) (Figure 1) or phylogenetic analysis (IAVP) (Figure 2). Furthermore, as described above, running MIX is possible to detect possible influenza coinfections in the sample.

## 4. Discussion

IAVC uses a simple circuit. In a first step, the most suitable reference for the genome of the analyzed virus is selected. Subsequently, this reference is used to map the reads to the reference and form a consensus when mismatches are detected. This approach is potentially universal. However, the current version of the algorithm is implemented for the influenza virus, whose genome consists of eight segments. As a result of an iterative search for the closest reference sequences and their further use to generate a consensus, corresponding consensuses are generated for each segment of the sequenced virus. The current version of the algorithm is only configured to assemble the genomes of those viruses whose genome consists of eight segments, which is a technical limitation of the algorithm. In order to use the pipeline, it is also necessary to use a comprehensive database with sequences of the corresponding virus type. IAVCP provides flexible and efficient processing of high-throughput sequencing data directly from raw reads. The pipeline aims to carefully process each viral segment, assemble it, and establish its evolutionary history.

**Figure 3:**
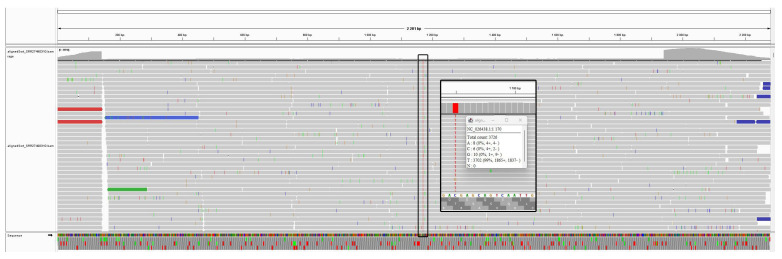
Visualization of the mapping of reads from SRR27490310 onto the reference genome A/California/07/2009(H1N1) using Integrative Genomics Viewer [51].

To assess the accuracy of assembly, three samples from experiment SRP483129 were used, where the IAV H1N1 virus was isolated from nasal wash of Mustela putorius: SRR27490307, SRR27490310, and SRR27490310. The consensus sequences of three samples obtained using IAVCP were compared with the assembly of influenza A virus (A/California/07/2009(H1N1)) GCF_001343785.1. The nucleotide sequence identity of the consensus sequences compared to (A/California/07/2009(H1N1)) was between 99% and 100%. Discrepant bases could be the result of natural viral variability or passaging in the laboratory. The alignment shows that the identified substitutions match the reads 100%, which is difficult to explain as an error in data processing (Figure 3). Full alignment of the consensus for each sample and the reference sequence A/California/07/2009(H1N1) are represented in Supplementary File S2.

It should be noted that INSaFLU [36] and IRMA (Iterative Refinement Meta-Assembler) [37] use a similar assembly strategy. INSaFLU assembles viral contigs de novo and compares them to 52 IAV reference viruses to maximize the accuracy of virus identification. In the case of reassortant viruses, the authors recommend the use of other tools (e.g., BEAST, Giraf, or BLAST) for the assignment of the closest publicly available sequence of each segment due to the incompleteness of the database used [36]. It should be noted that reassortment is likely to be a common event in the evolution of IAVs [52]. For the consensus sequences of the new virus generation using the INSaFLU reference sequences, user-defined sequences should be used, eliminating the possibility of fully automated analysis. IRMA overcomes these shortcomings and is widely used in recent publications describing new IAV sequences [52,53,54]. However, the databases used in this tool cannot be updated by users. At the same time, there is currently no gold standard for analyzing IAV high-throughput sequencing data. Some recent papers use the “manual reference selection and mapping to it” approach [55,56,57,58]. This shows the need to develop software that can do the same in an automated way.

It is worth noting that as a result of sequential processing, eight phylogenetic trees contain the same viruses for each segment. Therefore, the differences in the topology of these trees strongly indicate that the new virus is a reassortant. In addition, the tool is flexible and provides the ability to independently update the IAV segment databases while maintaining the file names, which is important for the correct operation of the pipeline.

## 5. Conclusions

IAVCP is a reliable tool for the automatic identification of the genomic sequence of influenza A virus (IAVC). In addition, another module of IAVCP (IAVP) automatically generates phylogenetic trees for all genomic segments of the virus of interest, which can be used to detect reassortment events. Thus, the presented tool can become a significant element in the surveillance of epidemiologically important variants of influenza A virus.

## Data Availability

Source code, manual, and dependent files are available in the GitHub repository (https://github.com/ana-way/IAVCP_workflow(accessed on 29 May 2024)). IAVCP db1 and db2 are available at https://github.com/ana-way/IAVCP_workflow/blob/main/data/data.tar.gz (accessed on 27 May 2024).

## Supplementary Materials

The following supporting information can be downloaded at: https://www.mdpi.com/article/10.3390/v16060873/s1, File S1: IAVCP report; File S2: Alignment of the consensus and the reference sequence A/California/07/2009(H1N1).
