# Supplementary material for: IAVCP (Influenza A Virus Consensus and Phylogeny): Automatic Identification of the Genomic Sequence of the Influenza A Virus from High-Throughput Sequencing Data
Source: Viruses. 2024 May 29;16(6):873. doi: 10.3390/v16060873 (PMC11209090; doi:10.3390/v16060873)
Supplement: Supplementary file 1 [file viruses-16-00873-s001.zip › viruses-2945568 suppl revised/S2_Supplementary file.pdf]

Supplementary

The two sequences alignment using BLASTN 2.15.0+

The reference found for SRR27490307 and the reference for Influenza A virus (A/California/07/2009(H1N1)) GCF_001343785.1.

1. Basic polymerase 2 (PB2)

```
Identities = 2279/2280 (99%), Gaps = 0/2280 (0%)

Query 27    ATGGAGAGAATAAAAGAACTGAGAGATCTAATGTCGCAGTCCCGCACTCGCGAGATACTC  86
            ||||||||||||||||||||||||||||||||||||||||||||||||||||||||||||
Sbjct 1     ATGGAGAGAATAAAAGAACTGAGAGATCTAATGTCGCAGTCCCGCACTCGCGAGATACTC  60

Query 87    ACTAAGACCACTGTGGACCATATGGCCATAATCAAAAAGTACACATCAGGAAGGCAAGAG  146
            ||||||||||||||||||||||||||||||||||||||||||||||||||||||||||||
Sbjct 61    ACTAAGACCACTGTGGACCATATGGCCATAATCAAAAAGTACACATCAGGAAGGCAAGAG  120

Query 147   AAGAACCCCGCACTCAGAATGAAGTGGATGATGGCAATGAGATACCCAATTACAGCAGAC  206
            ||||||||||||||||||||||||||||||||||||||||||||||||||||||||||||
Sbjct 121   AAGAACCCCGCACTCAGAATGAAGTGGATGATGGCAATGAGATACCCAATTACAGCAGAC  180

Query 207   AAGAGAATAATGGACATGATTCCAGAGAGGAATGAACAAGGACAAACCCTCTGGAGCAAA  266
            ||||||||||||||||||||||||||||||||||||||||||||||||||||||||||||
Sbjct 181   AAGAGAATAATGGACATGATTCCAGAGAGGAATGAACAAGGACAAACCCTCTGGAGCAAA  240

Query 267   ACAAACGATGCTGGATCAGACCGAGTGATGGTATCACCTCTGGCCGTAACATGGTGGAAT  326
            ||||||||||||||||||||||||||||||||||||||||||||||||||||||||||||
Sbjct 241   ACAAACGATGCTGGATCAGACCGAGTGATGGTATCACCTCTGGCCGTAACATGGTGGAAT  300

Query 327   AGGAATGGCCCAACAACAAGTACAGTTCATTACCCTAAGGTATATAAAACTTATTTCGAA  386
            ||||||||||||||||||||||||||||||||||||||||||||||||||||||||||||
Sbjct 301   AGGAATGGCCCAACAACAAGTACAGTTCATTACCCTAAGGTATATAAAACTTATTTCGAA  360

Query 387   AAGGTCGAAAGGTTGAAACATGGTACCTTCGGCCCTGTCCACTTCAGAAATCAAGTTAAA  446
            ||||||||||||||||||||||||||||||||||||||||||||||||||||||||||||
Sbjct 361   AAGGTCGAAAGGTTGAAACATGGTACCTTCGGCCCTGTCCACTTCAGAAATCAAGTTAAA  420

Query 447   ATAAGGAGGAGAGTTGATACAAACCCTGGCCATGCAGATCTCAGTGCCAAGGAGGCACAG  506
            ||||||||||||||||||||||||||||||||||||||||||||||||||||||||||||
Sbjct 421   ATAAGGAGGAGAGTTGATACAAACCCTGGCCATGCAGATCTCAGTGCCAAGGAGGCACAG  480

Query 507   GATGTGATTATGGAAGTTGTTTTCCCAAATGAAGTGGGGGCAAGAATACTGACATCAGAG  566
            ||||||||||||||||||||||||||||||||||||||||||||||||||||||||||||
Sbjct 481   GATGTGATTATGGAAGTTGTTTTCCCAAATGAAGTGGGGGCAAGAATACTGACATCAGAG  540

Query 567   TCACAGCTGGCAATAACAAAAGAGAAGAAAGAAGAGCTCCAGGATTGTAAAATTGCTCCC  626
            ||||||||||||||||||||||||||||||||||||||||||||||||||||||||||||
Sbjct 541   TCACAGCTGGCAATAACAAAAGAGAAGAAAGAAGAGCTCCAGGATTGTAAAATTGCTCCC  600

Query 627   TTGATGGTGGCGTACATGCTAGAAAGAGAATTGGTCCGTAAAACAAGGTTTCTCCCAGTA  686
            ||||||||||||||||||||||||||||||||||||||||||||||||||||||||||||
Sbjct 601   TTGATGGTGGCGTACATGCTAGAAAGAGAATTGGTCCGTAAAACAAGGTTTCTCCCAGTA  660

Query 687   GCCGGCGGAACAGGCAGTGTTTATATTGAAGTGTTGCACTTAACCCAAGGGACGTGCTGG  746
            ||||||||||||||||||||||||||||||||||||||||||||||||||||||||||||
Sbjct 661   GCCGGCGGAACAGGCAGTGTTTATATTGAAGTGTTGCACTTAACCCAAGGGACGTGCTGG  720

Query 747   GAGCAGATGTACACTCCAGGAGGAGAAGTGAGAAATGATGATGTTGACCAAAGTTTGATT  806
            ||||||||||||||||||||||||||||||||||||||||||||||||||||||||||||
Sbjct 721   GAGCAGATGTACACTCCAGGAGGAGAAGTGAGAAATGATGATGTTGACCAAAGTTTGATT  780

Query 807   ATCGCTGCTAGAAACATAGTAAGAAGAGCAGCAGTGTCAGCAGACCCATTAGCATCTCTC  866
            ||||||||||||||||||||||||||||||||||||||||||||||||||||||||||||
Sbjct 781   ATCGCTGCTAGAAACATAGTAAGAAGAGCAGCAGTGTCAGCAGACCCATTAGCATCTCTC  840

Query 867   TTGGAAATGTGCCACAGCACACAGATTGGAGGAGTAAGGATGGTGGACATCCTTAGACAG  926
            ||||||||||||||||||||||||||||||||||||||||||||||||||||||||||||
Sbjct 841   TTGGAAATGTGCCACAGCACACAGATTGGAGGAGTAAGGATGGTGGACATCCTTAGACAG  900
```

```
Query 927   AATCCAACTGAGGAACAAGCCGTAGACATATGCAAGGCAGCAATAGGGTTGAGGATTAGC  986
            ||||||||||||||||||||||||||||||||||||||||||||||||||||||||||||
Sbjct 901   AATCCAACTGAGGAACAAGCCGTAGACATATGCAAGGCAGCAATAGGGTTGAGGATTAGC  960

Query 987   TCATCTTTCAGTTTTGGTGGGTTCACTTTCAAAAGGACAAGCGGATCATCAGTCAAGAAA  1046
            ||||||||||||||||||||||||||||||||||||||||||||||||||||||||||||
Sbjct 961   TCATCTTTCAGTTTTGGTGGGTTCACTTTCAAAAGGACAAGCGGATCATCAGTCAAGAAA  1020

Query 1047  GAAGAAGAAGTGCTAACGGGCAACCTCCAAACACTGAAAATAAGAGTACATGAAGGGTAT  1106
            ||||||||||||||||||||||||||||||||||||||||||||||||||||||||||||
Sbjct 1021  GAAGAAGAAGTGCTAACGGGCAACCTCCAAACACTGAAAATAAGAGTACATGAAGGGTAT  1080

Query 1107  GAAGAATTCACAATGGTTGGGAGAAGAGCAACAGCTATTCTCAGAAAGGCAACCAGGAGA  1166
            ||||||||||||||||||||||||||||||||||||||||||||||||||||||||||||
Sbjct 1081  GAAGAATTCACAATGGTTGGGAGAAGAGCAACAGCTATTCTCAGAAAGGCAACCAGGAGA  1140

Query 1167  TTGATCCAGTTGATAGTAAGCGGGAGAGATGAGCAGTCAATTGCTGAGGCAATAATTGTG  1226
            ||||||||||||||||||||||||||||| ||||||||||||||||||||||||||||||
Sbjct 1141  TTGATCCAGTTGATAGTAAGCGGGAGAGACGAGCAGTCAATTGCTGAGGCAATAATTGTG  1200

Query 1227  GCCATGGTATTCTCACAGGAGGATTGCATGATCAAGGCAGTTAGGGGCGATCTGAACTTT  1286
            ||||||||||||||||||||||||||||||||||||||||||||||||||||||||||||
Sbjct 1201  GCCATGGTATTCTCACAGGAGGATTGCATGATCAAGGCAGTTAGGGGCGATCTGAACTTT  1260

Query 1287  GTCAATAGGGCAAACCAGCGACTGAACCCCATGCACCAACTCTTGAGGCATTTCCAAAAA  1346
            ||||||||||||||||||||||||||||||||||||||||||||||||||||||||||||
Sbjct 1261  GTCAATAGGGCAAACCAGCGACTGAACCCCATGCACCAACTCTTGAGGCATTTCCAAAAA  1320

Query 1347  GATGCAAAAGTGCTTTTCCAGAACTGGGGAATTGAATCCATCGACAATGTGATGGGAATG  1406
            ||||||||||||||||||||||||||||||||||||||||||||||||||||||||||||
Sbjct 1321  GATGCAAAAGTGCTTTTCCAGAACTGGGGAATTGAATCCATCGACAATGTGATGGGAATG  1380

Query 1407  ATCGGAATACTGCCCGACATGACCCCAAGCACGGAGATGTCGCTGAGAGGGATAAGAGTC  1466
            ||||||||||||||||||||||||||||||||||||||||||||||||||||||||||||
Sbjct 1381  ATCGGAATACTGCCCGACATGACCCCAAGCACGGAGATGTCGCTGAGAGGGATAAGAGTC  1440

Query 1467  AGCAAAATGGGAGTAGATGAATACTCCAGCACGGAGAGAGTGGTAGTGAGTATTGACCGA  1526
            ||||||||||||||||||||||||||||||||||||||||||||||||||||||||||||
Sbjct 1441  AGCAAAATGGGAGTAGATGAATACTCCAGCACGGAGAGAGTGGTAGTGAGTATTGACCGA  1500

Query 1527  TTTTTAAGGGTTAGAGATCAAAGAGGGAACGTACTATTGTCTCCCGAAGAAGTCAGTGAA  1586
            ||||||||||||||||||||||||||||||||||||||||||||||||||||||||||||
Sbjct 1501  TTTTTAAGGGTTAGAGATCAAAGAGGGAACGTACTATTGTCTCCCGAAGAAGTCAGTGAA  1560

Query 1587  ACGCAAGGAACTGAGAAGTTGACAATAACTTATTCGTCATCAATGATGTGGGAGATCAAT  1646
            ||||||||||||||||||||||||||||||||||||||||||||||||||||||||||||
Sbjct 1561  ACGCAAGGAACTGAGAAGTTGACAATAACTTATTCGTCATCAATGATGTGGGAGATCAAT  1620

Query 1647  GGCCCTGAGTCAGTGCTAGTCAACACTTATCAATGGATAATCAGGAACTGGGAAATTGTG  1706
            ||||||||||||||||||||||||||||||||||||||||||||||||||||||||||||
Sbjct 1621  GGCCCTGAGTCAGTGCTAGTCAACACTTATCAATGGATAATCAGGAACTGGGAAATTGTG  1680

Query 1707  AAAATTCAATGGTCACAAGATCCCACAATGTTATACAACAAAATGGAATTTGAACCATTT  1766
            ||||||||||||||||||||||||||||||||||||||||||||||||||||||||||||
Sbjct 1681  AAAATTCAATGGTCACAAGATCCCACAATGTTATACAACAAAATGGAATTTGAACCATTT  1740

Query 1767  CAGTCTCTTGTCCCTAAGGCAACCAGAAGCCGGTACAGTGGATTCGTAAGGACACTGTTC  1826
            ||||||||||||||||||||||||||||||||||||||||||||||||||||||||||||
Sbjct 1741  CAGTCTCTTGTCCCTAAGGCAACCAGAAGCCGGTACAGTGGATTCGTAAGGACACTGTTC  1800

Query 1827  CAGCAAATGCGGGATGTGCTTGGGACATTTGACACTGTCCAAATAATAAAACTTCTCCCC  1886
            ||||||||||||||||||||||||||||||||||||||||||||||||||||||||||||
Sbjct 1801  CAGCAAATGCGGGATGTGCTTGGGACATTTGACACTGTCCAAATAATAAAACTTCTCCCC  1860

Query 1887  TTTGCTGCTGCCCCACCAGAACAGAGTAGGATGCAATTTTCCTCATTGACTGTGAATGTG  1946
            ||||||||||||||||||||||||||||||||||||||||||||||||||||||||||||
Sbjct 1861  TTTGCTGCTGCCCCACCAGAACAGAGTAGGATGCAATTTTCCTCATTGACTGTGAATGTG  1920

Query 1947  AGAGGATCAGGGTTGAGGATACTGGTAAGAGGCAATTCTCCAGTATTCAATTACAACAAG  2006
            ||||||||||||||||||||||||||||||||||||||||||||||||||||||||||||
Sbjct 1921  AGAGGATCAGGGTTGAGGATACTGGTAAGAGGCAATTCTCCAGTATTCAATTACAACAAG  1980
```

```
Query 2007  GCAACCAAACGACTTACAGTTCTTGGAAAGGATGCAGGTGCATTGACTGAAGATCCAGAT  2066
            ||||||||||||||||||||||||||||||||||||||||||||||||||||||||||||
Sbjct 1981  GCAACCAAACGACTTACAGTTCTTGGAAAGGATGCAGGTGCATTGACTGAAGATCCAGAT  2040

Query 2067  GAAGGCACATCTGGGGTGGAGTCTGCTGTCCTGAGAGGATTTCTCATTTTGGGCAAAGAA  2126
            ||||||||||||||||||||||||||||||||||||||||||||||||||||||||||||
Sbjct 2041  GAAGGCACATCTGGGGTGGAGTCTGCTGTCCTGAGAGGATTTCTCATTTTGGGCAAAGAA  2100

Query 2127  GACAAGAGATATGGCCCAGCATTAAGCATCAATGAACTGAGCAATCTTGCAAAAGGAGAG  2186
            ||||||||||||||||||||||||||||||||||||||||||||||||||||||||||||
Sbjct 2101  GACAAGAGATATGGCCCAGCATTAAGCATCAATGAACTGAGCAATCTTGCAAAAGGAGAG  2160
Query 2187  AAGGCTAATGTGCTAATTGGGCAAGGGGACGTAGTGTTGGTAATGAAACGAAAACGGGAC  2246
            ||||||||||||||||||||||||||||||||||||||||||||||||||||||||||||
Sbjct 2161  AAGGCTAATGTGCTAATTGGGCAAGGGGACGTAGTGTTGGTAATGAAACGAAAACGGGAC  2220

Query 2247  TCTAGCATACTTACTGACAGCCAGACAGCGACCAAAAGAATTCGGATGGCCATCAATTAG  2306
            ||||||||||||||||||||||||||||||||||||||||||||||||||||||||||||
Sbjct 2221  TCTAGCATACTTACTGACAGCCAGACAGCGACCAAAAGAATTCGGATGGCCATCAATTAG  2280
```

2. Basic polymerase 1 (PB1)

```
Identities = 2273/2274 (99%), Gaps = 0/2274 (0%)

Query  10     ATGGATGTCAATCCGACTCTACTTTTCCTAAAAATTCCAGCGCAAAATGCCATAAGCACC  69
              ||||||||||||||||||||||||||||||||||||||||||||||||||||||||||||
Sbjct  1      ATGGATGTCAATCCGACTCTACTTTTCCTAAAAATTCCAGCGCAAAATGCCATAAGCACC  60

Query  70     ACATTCCCTTATACTGGAGATCCTCCATACAGCCATGGAACAGGAACAGGATACACCATG  129
              ||||||||||||||||||||||||||||||||||||||||||||||||||||||||||||
Sbjct  61     ACATTCCCTTATACTGGAGATCCTCCATACAGCCATGGAACAGGAACAGGATACACCATG  120

Query  130    GACACAGTAAACAGAACACACCAATACTCAGAAAAGGGAAAGTGGACGACAAACACAGAG  189
              ||||||||||||||||||||||||||||||||||||||||||||||||||||||||||||
Sbjct  121    GACACAGTAAACAGAACACACCAATACTCAGAAAAGGGAAAGTGGACGACAAACACAGAG  180

Query  190    ACTGGTGCACCCCAGCTCAACCCGATTGATGGACCACTACCTGAGGATAATGAACCAAGT  249
              ||||||||||||||||||||||||||||||||||||||||||||||||||||||||||||
Sbjct  181    ACTGGTGCACCCCAGCTCAACCCGATTGATGGACCACTACCTGAGGATAATGAACCAAGT  240

Query  250    GGGTATGCACAAACAGACTGTGTTCTAGAGGCTATGGCTTTCCTTGAAGAATCCCACCCA  309
              ||||||||||||||||||||||||||||||||||||||||||||||||||||||||||||
Sbjct  241    GGGTATGCACAAACAGACTGTGTTCTAGAGGCTATGGCTTTCCTTGAAGAATCCCACCCA  300

Query  310    GGAATATTTGAGAATTCATGCCTTGAAACAATGGAAGTTGTTCAACAAACAAGGGTAGAT  369
              ||||||||||||||||||||||||||||||||||||||||||||||||||||||||||||
Sbjct  301    GGAATATTTGAGAATTCATGCCTTGAAACAATGGAAGTTGTTCAACAAACAAGGGTAGAT  360

Query  370    AAACTAACTCAAGGTCGCCAGACTTATGATTGGACATTAAACAGAAATCAACCGGCAGCA  429
              ||||||||||||||||||||||||||||||||||||||||||||||||||||||||||||
Sbjct  361    AAACTAACTCAAGGTCGCCAGACTTATGATTGGACATTAAACAGAAATCAACCGGCAGCA  420

Query  430    ACTGCATTGGCCAACACCATAGAAGTCTTTAGATCGAATGGCCTAACAGCTAATGAGTCA  489
              ||||||||||||||||||||||||||||||||||||||||||||||||||||||||||||
Sbjct  421    ACTGCATTGGCCAACACCATAGAAGTCTTTAGATCGAATGGCCTAACAGCTAATGAGTCA  480

Query  490    GGAAGGCTAATAGATTTCTTAAAGGATGTAATGGAATCAATGAACAAAGAGGAAATAGAG  549
              ||||||||||||||||||||||||||||||||||||||||||||||||||||||||||||
Sbjct  481    GGAAGGCTAATAGATTTCTTAAAGGATGTAATGGAATCAATGAACAAAGAGGAAATAGAG  540

Query  550    ATAACAACCCACTTTCAAAGAAAAAGGAGAGTAAGAGACAACATGACCAAGAAGATGGTC  609
              ||||||||||||||||||||||||||||||||||||||||||||||||||||||||||||
Sbjct  541    ATAACAACCCACTTTCAAAGAAAAAGGAGAGTAAGAGACAACATGACCAAGAAGATGGTC  600

Query  610    ACGCAAAGAACAATAGGGAAGAAAAAACAAAGACTGAATAAGAGAGGCTATCTAATAAGA  669
              ||||||||||||||||||||||||||||||||||||||||||||||||||||||||||||
Sbjct  601    ACGCAAAGAACAATAGGGAAGAAAAAACAAAGACTGAATAAGAGAGGCTATCTAATAAGA  660

Query  670    GCACTGACATTAAATACGATGACCAAAGATGCAGAGAGAGGCAAGTTAAAAAGAAGGGCT  729
              ||||||||||||||||||||||||||||||||||||||||||||||||||||||||||||
Sbjct  661    GCACTGACATTAAATACGATGACCAAAGATGCAGAGAGAGGCAAGTTAAAAAGAAGGGCT  720

Query  730    ATCGCAACACCTGGGATGCAGATTAGAGGTTTCGTATACTTTGTTGAAACTTTAGCTAGG  789
```

```
                ||||||||||||||||||||||||||||||||||||||||||||||||||||||||||||
Sbjct  721      ATCGCAACACCTGGGATGCAGATTAGAGGTTTCGTATACTTTGTTGAAACTTTAGCTAGG  780
Query  790      AGCATTTGCGAAAAGCTTGAACAGTCTGGGCTCCCAGTAGGGGGCAATGAAAAGAAGGCC  849
                ||||||||||||||||||||||||||||||||||||||||||||||||||||||||||||
Sbjct  781      AGCATTTGCGAAAAGCTTGAACAGTCTGGGCTCCCAGTAGGGGGCAATGAAAAGAAGGCC  840

Query  850      AAACTGGCAAATGTTGTGAGAAAGATGATGACTAATTCACAAGACACAGAGATTTCTTTC  909
                ||||||||||||||||||||||||||||||||||||||||||||||||||||||||||||
Sbjct  841      AAACTGGCAAATGTTGTGAGAAAGATGATGACTAATTCACAAGACACAGAGATTTCTTTC  900

Query  910      ACAATCACTGGGGACAACACTAAGTGGAATGAAAATCAAAATCCTCGAATGTTCCTGGCG  969
                ||||||||||||||||||||||||||||||||||||||||||||||||||||||||||||
Sbjct  901      ACAATCACTGGGGACAACACTAAGTGGAATGAAAATCAAAATCCTCGAATGTTCCTGGCG  960

Query  970      ATGATTACATATATCACCAGAAATCAACCCGAGTGGTTCAGAAACATCCTGAGCATGGCA  1029
                ||||||||||||||||||||||||||||||||||||||||||||||||||||||||||||
Sbjct  961      ATGATTACATATATCACCAGAAATCAACCCGAGTGGTTCAGAAACATCCTGAGCATGGCA  1020

Query  1030     CCCATAATGTTCTCAAACAAAATGGCAAGACTAGGGAAAGGGTACATGTTCGAGAGTAAA  1089
                ||||||||||||||||||||||||||||||||||||||||||||||||||||||||||||
Sbjct  1021     CCCATAATGTTCTCAAACAAAATGGCAAGACTAGGGAAAGGGTACATGTTCGAGAGTAAA  1080

Query  1090     AGAATGAAGATTCGAACACAAATACCAGCAGAAATGCTAGCAAGCATTGACCTGAAGTAC  1149
                ||||||||||||||||||||||||||||||||||||||||||||||||||||||||||||
Sbjct  1081     AGAATGAAGATTCGAACACAAATACCAGCAGAAATGCTAGCAAGCATTGACCTGAAGTAC  1140

Query  1150     TTCAATGAATCAACAAAGAAGAAAATTGAGAAAATAAGGCCTCTTCTAATAGATGGCACA  1209
                ||||||||||||||||||||||||||||||||||||||||||||||||||||||||||||
Sbjct  1141     TTCAATGAATCAACAAAGAAGAAAATTGAGAAAATAAGGCCTCTTCTAATAGATGGCACA  1200

Query  1210     GCATCACTGAGTCCTGGGATGATGATGGGCATGTTCAACATGCTAAGTACGGTCTTGGGA  1269
                ||||||||||||||||||||||||||||||||||||||||||||||||||||||||||||
Sbjct  1201     GCATCACTGAGTCCTGGGATGATGATGGGCATGTTCAACATGCTAAGTACGGTCTTGGGA  1260

Query  1270     GTCTCGATACTGAATCTTGGACAAAAGAAATACACCAAGACAATATACTGGTGGGATGGG  1329
                ||||||||||||||||||||||||||||||||||||||||||||||||||||||||||||
Sbjct  1261     GTCTCGATACTGAATCTTGGACAAAAGAAATACACCAAGACAATATACTGGTGGGATGGG  1320

Query  1330     CTCCAATCATCCGACGATTTTGCTCTCATAGTGAATGCACCAAACCATGAGGGAATACAA  1389
                ||||||||||||||||||||||||||||||||||||||||||||||||||||||||||||
Sbjct  1321     CTCCAATCATCCGACGATTTTGCTCTCATAGTGAATGCACCAAACCATGAGGGAATACAA  1380

Query  1390     GCAGGAGTGGACAGATTCTACAGGACCTGCAAGTTAGTGGGAATCAACATGAGCAAAAAG  1449
                ||||||||||||||||||||||||||||||||||||||||||||||||||||||||||||
Sbjct  1381     GCAGGAGTGGACAGATTCTACAGGACCTGCAAGTTAGTGGGAATCAACATGAGCAAAAAG  1440

Query  1450     AAGTCCTATATAAATAAGACAGGGACATTTGAATTCACAAGCtttttttATCGCTATGGA  1509
                ||||||||||||||||||||||||||||||||||||||||||||||||||||||||||||
Sbjct  1441     AAGTCCTATATAAATAAGACAGGGACATTTGAATTCACAAGCTTTTTTTATCGCTATGGA  1500

Query  1510     TTTGTGGCTAATTTTAGCATGGAGCTACCCAGCTTTGGAGTGTCTGGAGTAAATGAATCA  1569
                ||||||||||||||||||||||||||||||||||||||||||||||||||||||||||||
Sbjct  1501     TTTGTGGCTAATTTTAGCATGGAGCTACCCAGCTTTGGAGTGTCTGGAGTAAATGAATCA  1560

Query  1570     GCTGACATGAGTATTGGAGTAACAGTGATAAAGAACAACATGATAAACAATGACCTTGGA  1629
                ||||||||||||||||||||||||||||||||||||||||||||||||||||||||||||
Sbjct  1561     GCTGACATGAGTATTGGAGTAACAGTGATAAAGAACAACATGATAAACAATGACCTTGGA  1620

Query  1630     CCTGCAACGGCCCAGATGGCTCTTCAATTGTTCATCAAAGACTACAGATACACATATAGG  1689
                ||||||||||||||||||||||||||||||||||||||||||||||||||||||||||||
Sbjct  1621     CCTGCAACGGCCCAGATGGCTCTTCAATTGTTCATCAAAGACTACAGATACACATATAGG  1680

Query  1690     TGCCATAGGGGAGACACACAAATTCAGACGAGAAGATCATTTGAGTTAAAGAAGCTGTGG  1749
                ||||||||||||||||||||||||||||||||||||||||||||||||||||||||||||
Sbjct  1681     TGCCATAGGGGAGACACACAAATTCAGACGAGAAGATCATTTGAGTTAAAGAAGCTGTGG  1740

Query  1750     GATCAAACCCAATCAAAAGTAGGGCTATTAGTATCAGATGGAGGACCAAACTTATACAAT  1809
                ||||||||||||||||| ||||||||||||||||||||||||||||||||||||||||||
Sbjct  1741     GATCAAACCCAATCAAAGGTAGGGCTATTAGTATCAGATGGAGGACCAAACTTATACAAT  1800

Query  1810     ATACGGAATCTTCACATTCCTGAAGTCTGCTTAAAATGGGAGCTAATGGATGATGATTAT  1869
                ||||||||||||||||||||||||||||||||||||||||||||||||||||||||||||
```

```
Sbjct  1801   ATACGGAATCTTCACATTCCTGAAGTCTGCTTAAAATGGGAGCTAATGGATGATGATTAT  1860

Query  1870   CGGGGAAGACTTTGTAATCCCCTGAATCCCTTTGTCAGTCATAAAGAGATTGATTCTGTA  1929
              ||||||||||||||||||||||||||||||||||||||||||||||||||||||||||||
Sbjct  1861   CGGGGAAGACTTTGTAATCCCCTGAATCCCTTTGTCAGTCATAAAGAGATTGATTCTGTA  1920

Query  1930   AACAATGCTGTGGTAATGCCAGCCCATGGTCCAGCCAAAAGCATGGAATATGATGCCGTT  1989
              ||||||||||||||||||||||||||||||||||||||||||||||||||||||||||||
Sbjct  1921   AACAATGCTGTGGTAATGCCAGCCCATGGTCCAGCCAAAAGCATGGAATATGATGCCGTT  1980

Query  1990   GCAACTACACATTCCTGGATTCCCAAGAGGAATCGTTCTATTCTCAACACAAGCCAAAGG  2049
              ||||||||||||||||||||||||||||||||||||||||||||||||||||||||||||
Sbjct  1981   GCAACTACACATTCCTGGATTCCCAAGAGGAATCGTTCTATTCTCAACACAAGCCAAAGG  2040

Query  2050   GGAATTCTTGAGGATGAACAGATGTACCAGAAGTGCTGCAATCTATTCGAGAAATTTTTC  2109
              ||||||||||||||||||||||||||||||||||||||||||||||||||||||||||||
Sbjct  2041   GGAATTCTTGAGGATGAACAGATGTACCAGAAGTGCTGCAATCTATTCGAGAAATTTTTC  2100

Query  2110   CCTAGCAGTTCATATAGGAGACCGGTTGGAATTTCTAGCATGGTGGAGGCCATGGTGTCT  2169
              ||||||||||||||||||||||||||||||||||||||||||||||||||||||||||||
Sbjct  2101   CCTAGCAGTTCATATAGGAGACCGGTTGGAATTTCTAGCATGGTGGAGGCCATGGTGTCT  2160

Query  2170   AGGGCCCGGATTGATGCCAGGGTCGACTTCGAGTCTGGACGGATCAAGAAAGAAGAGTTC  2229
              ||||||||||||||||||||||||||||||||||||||||||||||||||||||||||||
Sbjct  2161   AGGGCCCGGATTGATGCCAGGGTCGACTTCGAGTCTGGACGGATCAAGAAAGAAGAGTTC  2220

Query  2230   TCTGAGATCATGAAGATCTGTTCCACCATTGAAGAACTCAGACGGCAAAAAATAA  2283
              ||||||||||||||||||||||||||||||||||||||||||||||||||||||
Sbjct  2221   TCTGAGATCATGAAGATCTGTTCCACCATTGAAGAACTCAGACGGCAAAAAATAA  2274
```

3. Acidic polymerase (PA)

```
Identities = 2148/2150 (99%), Gaps = 0/2150 (0%)
Query  24     ATGGAAGACTTTGTGCGACAATGCTTCAATCCAATGATCGTCGAGCTTGCGGAAAAGGCA  83
              |||||||||||||||||||||||||||||||||||||||||||||||||||| |||||||
Sbjct  1      ATGGAAGACTTTGTGCGACAATGCTTCAATCCAATGATCGTCGAGCTTGCGGRAAAGGCA  60

Query  84     ATGAAAGAATATGGGGAAGATCCGAAAATCGAAACTAACAAGTTTGCTGCAATATGCACA  143
              ||||||||||||||||||||||||||||||||||||||||||||||||||||||||||||
Sbjct  61     ATGAAAGAATATGGGGAAGATCCGAAAATCGAAACTAACAAGTTTGCTGCAATATGCACA  120

Query  144    CATTTGGAAGTTTGTTTCATGTATTCGGATTTCCATTTCATCGACGAACGGGGTGAATCA  203
              ||||||||||||||||||||||||||||||||||||||||||||||||||||||||||||
Sbjct  121    CATTTGGAAGTTTGTTTCATGTATTCGGATTTCCATTTCATCGACGAACGGGGTGAATCA  180

Query  204    ATAATTGTAGAATCTGGTGACCCGAATGCACTATTGAAGCACCGATTTGAGATAATTGAA  263
              ||||||||||||||||||||||||||||||||||||||||||||||||||||||||||||
Sbjct  181    ATAATTGTAGAATCTGGTGACCCGAATGCACTATTGAAGCACCGATTTGAGATAATTGAA  240

Query  264    GGAAGAGACCGAATCATGGCCTGGACAGTGGTGAACAGTATATGTAACACAACAGGGGTA  323
              ||||||||||||||||||||||||||||||||||||||||||||||||||||||||||||
Sbjct  241    GGAAGAGACCGAATCATGGCCTGGACAGTGGTGAACAGTATATGTAACACAACAGGGGTA  300

Query  324    GAGAAGCCTAAATTTCTTCCTGATTTGTATGATTACAAAGAGAACCGGTTCATTGAAATT  383
              ||||||||||||||||||||||||||||||||||||||||||||||||||||||||||||
Sbjct  301    GAGAAGCCTAAATTTCTTCCTGATTTGTATGATTACAAAGAGAACCGGTTCATTGAAATT  360

Query  384    GGAGTAACACGGAGGGAAGTCCACATATATTACCTAGAGAAAGCCAACAAAATAAAATCT  443
              ||||||||||||||||||||||||||||||||||||||||||||||||||||||||||||
Sbjct  361    GGAGTAACACGGAGGGAAGTCCACATATATTACCTAGAGAAAGCCAACAAAATAAAATCT  420

Query  444    GAGAAGACACACATTCACATCTTTTCATTCACTGGAGAGGAGATGGCCACCAAAGCGGAC  503
              ||||||||||||||||||||||||||||||||||||||||||||||||||||||||||||
Sbjct  421    GAGAAGACACACATTCACATCTTTTCATTCACTGGAGAGGAGATGGCCACCAAAGCGGAC  480

Query  504    TACACCCTTGACGAAGAGAGCAGGGCAAGAATCAAAACTAGGCTTTTCACTATAAGACAA  563
              ||||||||||||||||||||||||||||||||||||||||||||||||||||||||||||
Sbjct  481    TACACCCTTGACGAAGAGAGCAGGGCAAGAATCAAAACTAGGCTTTTCACTATAAGACAA  540

Query  564    GAAATGGCCAGTAGGAGTCTATGGGATTCCTTTCGTCAGTCCGAAAGAGGCGAAGAGACA  623
              ||||||||||||||||||||||||||||||||||||||||||||||||||||||||||||
```

```
Sbjct  541   GAAATGGCCAGTAGGAGTCTATGGGATTCCTTTCGTCAGTCCGAAAGAGGCGAAGAGACA  600

Query  624   ATTGAAGAAAAATTTGAGATTACAGGAACTATGCGCAAGCTTGCCGACCAAAGTCTCCCA  683
             ||||||||||||||||||||||||||||||||||||||||||||||||||||||||||||
Sbjct  601   ATTGAAGAAAAATTTGAGATTACAGGAACTATGCGCAAGCTTGCCGACCAAAGTCTCCCA  660

Query  684   CCGAACTTCCCCAGCCTTGAAAACTTTAGAGCCTATGTAGATGGATTCGAGCCGAACGGC  743
             ||||||||||||||||||||||||||||||||||||||||||||||||||||||||||||
Sbjct  661   CCGAACTTCCCCAGCCTTGAAAACTTTAGAGCCTATGTAGATGGATTCGAGCCGAACGGC  720

Query  744   TGCATTGAGGGCAAGCTTTCCCAAATGTCAAAAGAAGTGAACGCCAAAATTGAACCATTC  803
             ||||||||||||||||||||||||||||||||||||||||||||||||||||||||||||
Sbjct  721   TGCATTGAGGGCAAGCTTTCCCAAATGTCAAAAGAAGTGAACGCCAAAATTGAACCATTC  780

Query  804   TTGAGGACGACACCACGCCCCCTCAGATTGCCTGATGGGCCTCTTTGCCATCAGCGGTCA  863
             ||||||||||||||||||||||||||||||||||||||||||||||||||||||||||||
Sbjct  781   TTGAGGACGACACCACGCCCCCTCAGATTGCCTGATGGGCCTCTTTGCCATCAGCGGTCA  840

Query  864   AAGTTCCTGCTGATGGATGCTCTGAAATTAAGTATTGAAGACCCGAGTCACGAGGGGGAG  923
             ||||||||||||||||||||||||||||||||||||||||||||||||||||||||||||
Sbjct  841   AAGTTCCTGCTGATGGATGCTCTGAAATTAAGTATTGAAGACCCGAGTCACGAGGGGGAG  900

Query  924   GGAATACCACTATATGATGCAATCAAATGCATGAAGACATTCTTTGGCTGGAAAGAGCCT  983
             ||||||||||||||||||||||||||||||||||||||||||||||||||||||||||||
Sbjct  901   GGAATACCACTATATGATGCAATCAAATGCATGAAGACATTCTTTGGCTGGAAAGAGCCT  960

Query  984   AACATAGTCAAACCACATGAGAAAGGCATAAATCCCAATTACCTCATGGCTTGGAAGCAG  1043
             ||||||||||||||||||||||||||||||||||||||||||||||||||||||||||||
Sbjct  961   AACATAGTCAAACCACATGAGAAAGGCATAAATCCCAATTACCTCATGGCTTGGAAGCAG  1020

Query  1044  GTGCTAGCAGAGCTACAGGACATTGAAAATGAAGAGAAGATCCCAAGGACAAAGAACATG  1103
             ||||||||||||||||||||||||||||||||||||||||||||||||||||||||||||
Sbjct  1021  GTGCTAGCAGAGCTACAGGACATTGAAAATGAAGAGAAGATCCCAAGGACAAAGAACATG  1080

Query  1104  AAGAGAACAAGCCAATTGAAGTGGGCACTCGGTGAAAATATGGCACCAGAAAAAGTAGAC  1163
             ||||||||||||||||||||||||||||||||||||||||||||||||||||||||||||
Sbjct  1081  AAGAGAACAAGCCAATTGAAGTGGGCACTCGGTGAAAATATGGCACCAGAAAAAGTAGAC  1140

Query  1164  TTTGATGACTGCAAAGATGTTGGAGACCTTAAACAGTATGACAGTGATGAGCCAGAGCCC  1223
             ||||||||||||||||||||||||||||||||||||||||||||||||||||||||||||
Sbjct  1141  TTTGATGACTGCAAAGATGTTGGAGACCTTAAACAGTATGACAGTGATGAGCCAGAGCCC  1200

Query  1224  AGATCTCTAGCAAGCTGGGTCCAAAATGAATTCAATAAGGCATGTGAATTGACTGATTCA  1283
             ||||||||||||||||||||||||||||||||||||||||||||||||||||||||||||
Sbjct  1201  AGATCTCTAGCAAGCTGGGTCCAAAATGAATTCAATAAGGCATGTGAATTGACTGATTCA  1260

Query  1284  AGCTGGATAGAACTTGATGAAATAGGAGAAGATGTTGCCCCGATTGAACATATCGCAAGC  1343
             ||||||||||||||||||||||||||||||||||||||||||||||||||||||||||||
Sbjct  1261  AGCTGGATAGAACTTGATGAAATAGGAGAAGATGTTGCCCCGATTGAACATATCGCAAGC  1320

Query  1344  ATGAGGAGGAACTATTTTACAGCAGAAGTGTCCCACTGCAGGGCTACTGAATACATAATG  1403
             ||||||||||||||||||||||||||||||||||||||||||||||||||||||||||||
Sbjct  1321  ATGAGGAGGAACTATTTTACAGCAGAAGTGTCCCACTGCAGGGCTACTGAATACATAATG  1380

Query  1404  AAGGGAGTGTACATAAATACGGCCTTGCTCAATGCATCCTGTGCAGCCATGGATGACTTT  1463
             ||||||||||||||||||||||||||||||||||||||||||||||||||||||||||||
Sbjct  1381  AAGGGAGTGTACATAAATACGGCCTTGCTCAATGCATCCTGTGCAGCCATGGATGACTTT  1440

Query  1464  CAGCTGATCCCAATGATAAGCAAATGTAGGACCAAAGAAGGAAGACGGAAAACAAACCTG  1523
             ||||||||||||||||||||||||||||||||||||||||||||||||||||||||||||
Sbjct  1441  CAGCTGATCCCAATGATAAGCAAATGTAGGACCAAAGAAGGAAGACGGAAAACAAACCTG  1500

Query  1524  TATGGGTTCATTATAAAAGGAAGGTCTCATTTGAGAAATGATACTGATGTGGTGAACTTT  1583
             ||||||||||||||||||||||||||||||||||||||||||||||||||||||||||||
Sbjct  1501  TATGGGTTCATTATAAAAGGAAGGTCTCATTTGAGAAATGATACTGATGTGGTGAACTTT  1560

Query  1584  GTAAGTATGGAGTTCTCACTCACTGACCCGAGACTGGAGCCACACAAATGGGAAAAATAC  1643
             ||||||||||||||||||||||||||||||||||||||||||||||||||||||||||||
Sbjct  1561  GTAAGTATGGAGTTCTCACTCACTGACCCGAGACTGGAGCCACACAAATGGGAAAAATAC  1620

Query  1644  TGTGTTCTTGAAATAGGAGACATGCTCTTGAGGACTGCGATAGGCCAAGTGTCGAGGCCC  1703
             ||||||||||||||||||||||||||||||||||||||||||||||||||||||||||||
```

```
Sbjct  1621   TGTGTTCTTGAAATAGGAGACATGCTCTTGAGGACTGCGATAGGCCAAGTGTCGAGGCCC  1680

Query  1704   ATGTTCCTATATGTGAGAACCAATGGAACCTCCAAGATCAAGATGAAATGGGGCATGGAA  1763
              ||||||||||||||||||||||||||||||||||||||||||||||||||||||||||||
Sbjct  1681   ATGTTCCTATATGTGAGAACCAATGGAACCTCCAAGATCAAGATGAAATGGGGCATGGAA  1740

Query  1764   ATGAGGCGCTGCCTTCTTCAGTCTCTTCAGCAGATTGAGAGCATGATTGAGGCCGAGTCT  1823
              ||||||||||||||||||||||||||||||||||||||||||||||||||||||||||||
Sbjct  1741   ATGAGGCGCTGCCTTCTTCAGTCTCTTCAGCAGATTGAGAGCATGATTGAGGCCGAGTCT  1800

Query  1824   TCTGTCAAAGAGAAAGACATGACCAAGGAATTCTTTGAAAACAAATCGGAAACATGGCCA  1883
              ||||||||||||||||||||||||||||||||||||||||||||||||||||||||||||
Sbjct  1801   TCTGTCAAAGAGAAAGACATGACCAAGGAATTCTTTGAAAACAAATCGGAAACATGGCCA  1860

Query  1884   ATCGGAGAGTCACCCAGGGGAGTGGAGGAAGGCTCTATTGGGAAAGTGTGCAGGACCTTA  1943
              ||||||||||||||||||||||||||||||||||||||||||||||||||||||||||||
Sbjct  1861   ATCGGAGAGTCACCCAGGGGAGTGGAGGAAGGCTCTATTGGGAAAGTGTGCAGGACCTTA  1920

Query  1944   CTGGCAAAATCTGTATTCAACAGTCTATATGCATCTCCACAACTTGAGGGGTTTTCGGCT  2003
              |||||||||||||||||||||||||||||||| |||||||||||||||||||||||||||
Sbjct  1921   CTGGCAAAATCTGTATTCAACAGTCTATATGCGTCTCCACAACTTGAGGGGTTTTCGGCT  1980

Query  2004   GAATCTAGAAAATTGCTTCTCATTGTTCAGGCACTTAGGGACAACCTGGAACCTGGAACC  2063
              ||||||||||||||||||||||||||||||||||||||||||||||||||||||||||||
Sbjct  1981   GAATCTAGAAAATTGCTTCTCATTGTTCAGGCACTTAGGGACAACCTGGAACCTGGAACC  2040

Query  2064   TTCGATCTTGGGGGGCTATATGAAGCAATCGAGGAGTGCCTGATTAATGATCCCTGGGTT  2123
              ||||||||||||||||||||||||||||||||||||||||||||||||||||||||||||
Sbjct  2041   TTCGATCTTGGGGGGCTATATGAAGCAATCGAGGAGTGCCTGATTAATGATCCCTGGGTT  2100

Query  2124   TTGCTTAATGCATCTTGGTTCAACTCCTTCCTCACACATGCACTGAAGTA  2173
              ||||||||||||||||||||||||||||||||||||||||||||||||||
Sbjct  2101   TTGCTTAATGCATCTTGGTTCAACTCCTTCCTCACACATGCACTGAAGTA  2150
```

4. Hemagglutinin (HA)

```
Identities = 1685/1688 (99%), Gaps = 0/1688 (0%)
Query  10     TACTAGTAGTTCTGCTATATACATTTGCAACCGCAAATGCAGACACATTATGTATAGGTT  69
              ||||||||||||||||||||||||||||||||||||||||||||||||||||||||||||
Sbjct  11     TACTAGTAGTTCTGCTATATACATTTGCAACCGCAAATGCAGACACATTATGTATAGGTT  70

Query  70     ATCATGCGAACAATTCAACAGACACTGTAGACACAGTACTAGAAAAGAATGTAACAGTAA  129
              ||||||||||||||||||||||||||||||||||||||||||||||||||||||||||||
Sbjct  71     ATCATGCGAACAATTCAACAGACACTGTAGACACAGTACTAGAAAAGAATGTAACAGTAA  130

Query  130    CACACTCTGTTAACCTTCTAGAAGACAAGCATAACGGGAAACTATGCAAACTAAGAGGGG  189
              ||||||||||||||||||||||||||||||||||||||||||||||||||||||||||||
Sbjct  131    CACACTCTGTTAACCTTCTAGAAGACAAGCATAACGGGAAACTATGCAAACTAAGAGGGG  190

Query  190    TAGCCCCATTGCATTTGGGTAAATGTAACATTGCTGGCTGGATCCTGGGAAATCCAGAGT  249
              ||||||||||||||||||||||||||||||||||||||||||||||||||||||||||||
Sbjct  191    TAGCCCCATTGCATTTGGGTAAATGTAACATTGCTGGCTGGATCCTGGGAAATCCAGAGT  250

Query  250    GTGAATCACTCTCCACAGCAAGCTCATGGTCCTACATTGTGGAAACACCTAGTTCAGACA  309
              ||||||||||||||||||||||||||||||||||||||||||||||||||||||||||||
Sbjct  251    GTGAATCACTCTCCACAGCAAGCTCATGGTCCTACATTGTGGAAACACCTAGTTCAGACA  310

Query  310    ATGGAACGTGTTACCCAGGAGATTTCATCGATTATGAGGAGCTAAGAGAGCAATTGAGCT  369
              ||||||||||||||||||||||||||||||||||||||||||||||||||||||||||||
Sbjct  311    ATGGAACGTGTTACCCAGGAGATTTCATCGATTATGAGGAGCTAAGAGAGCAATTGAGCT  370

Query  370    CAGTGTCATCATTTGAAAGGTTTGAGATATTCCCCAAGACAAGTTCATGGCCCAATCATG  429
              ||||||||||||||||||||||||||||||||||||||||||||||||||||||||||||
Sbjct  371    CAGTGTCATCATTTGAAAGGTTTGAGATATTCCCCAAGACAAGTTCATGGCCCAATCATG  430

Query  430    ACTCGAACAAAGGTGTAACGGCAGCATGTCCTCATGCTGGAGCAAAAAGCTTCTACAAAA  489
              ||||||||||||||||||||||||||||||||||||||||||||||||||||||||||||
Sbjct  431    ACTCGAACAAAGGTGTAACGGCAGCATGTCCTCATGCTGGAGCAAAAAGCTTCTACAAAA  490

Query  490    ATTTAATATGGCTAGTTAAAAAAGAAAATTCATACCCAAAGCTCAGCAAATCCTACATTA  549
              |||||||||||||||||||||||| |||||||||||||||||||||||||||||||||||
```

```
Sbjct  491   ATTTAATATGGCTAGTTAAAAAAGGAAATTCATACCCAAAGCTCAGCAAATCCTACATTA  550

Query  550   ATGATAAAGGGAAAGAAGTCCTCGTGCTATGGGGCATTCACCATCCATCTACTAGTGCTG  609
             ||||||||||||||||||||||||||||||||||||||||||||||||||||||||||||
Sbjct  551   ATGATAAAGGGAAAGAAGTCCTCGTGCTATGGGGCATTCACCATCCATCTACTAGTGCTG  610

Query  610   ACCAACAAAGTCTCTATCAGAATGCAGATGCATATGTTTTTGTGGGGTCATCAAGATACA  669
             ||||||||||||||||||||||||||||||||||||||||||||||||||||||||||||
Sbjct  611   ACCAACAAAGTCTCTATCAGAATGCAGATGCATATGTTTTTGTGGGGTCATCAAGATACA  670

Query  670   GCAAGAAGTTCAAGCCGGAAATAGCAATAAGACCCAAAGTGAGGGATCAAGAAGGGAGAA  729
             ||||||||||||||||||||||||||||||||||||||||||||| || |||||||||||
Sbjct  671   GCAAGAAGTTCAAGCCGGAAATAGCAATAAGACCCAAAGTGAGGGRTCRAGAAGGGAGAA  730

Query  730   TGAACTATTACTGGACACTAGTAGAGCCGGGAGACAAAATAACATTCGAAGCAACTGGAA  789
             ||||||||||||||||||||||||||||||||||||||||||||||||||||||||||||
Sbjct  731   TGAACTATTACTGGACACTAGTAGAGCCGGGAGACAAAATAACATTCGAAGCAACTGGAA  790

Query  790   ATCTAGTGGTACCGAGATATGCATTCGCAATGGAAAGAAATGCTGGATCTGGTATTATCA  849
             ||||||||||||||||||||||||||||||||||||||||||||||||||||||||||||
Sbjct  791   ATCTAGTGGTACCGAGATATGCATTCGCAATGGAAAGAAATGCTGGATCTGGTATTATCA  850

Query  850   TTTCAGATACACCAGTCCACGATTGCAATACAACTTGTCAAACACCCAAGGGTGCTATAA  909
             ||||||||||||||||||||||||||||||||||||||||||||||||||||||||||||
Sbjct  851   TTTCAGATACACCAGTCCACGATTGCAATACAACTTGTCAAACACCCAAGGGTGCTATAA  910

Query  910   ACACCAGCCTCCCATTTCAGAATATACATCCGATCACAATTGGAAAATGTCCAAAATATG  969
             ||||||||||||||||||||||||||||||||||||||||||||||||||||||||||||
Sbjct  911   ACACCAGCCTCCCATTTCAGAATATACATCCGATCACAATTGGAAAATGTCCAAAATATG  970

Query  970   TAAAAAGCACAAAATTGAGACTGGCCACAGGATTGAGGAATATCCCGTCTATTCAATCTA  1029
             ||||||||||||||||||||||||||||||||||||||||||||||||||||||||||||
Sbjct  971   TAAAAAGCACAAAATTGAGACTGGCCACAGGATTGAGGAATATCCCGTCTATTCAATCTA  1030

Query  1030  GAGGCCTATTTGGGGCCATTGCCGGTTTCATTGAAGGGGGGTGGACAGGGATGGTAGATG  1089
             ||||||||||||||||||||||||||||||||||||||||||||||||||||||||||||
Sbjct  1031  GAGGCCTATTTGGGGCCATTGCCGGTTTCATTGAAGGGGGGTGGACAGGGATGGTAGATG  1090

Query  1090  GATGGTACGGTTATCACCATCAAAATGAGCAGGGGTCAGGATATGCAGCCGACCTGAAGA  1149
             ||||||||||||||||||||||||||||||||||||||||||||||||||||||||||||
Sbjct  1091  GATGGTACGGTTATCACCATCAAAATGAGCAGGGGTCAGGATATGCAGCCGACCTGAAGA  1150

Query  1150  GCACACAGAATGCCATTGACGAGATTACTAACAAAGTAAATTCTGTTATTGAAAAGATGA  1209
             ||||||||||||||||||||||||||||||||||||||||||||||||||||||||||||
Sbjct  1151  GCACACAGAATGCCATTGACGAGATTACTAACAAAGTAAATTCTGTTATTGAAAAGATGA  1210

Query  1210  ATACACAGTTCACAGCAGTAGGTAAAGAGTTCAACCACCTGGAAAAAAGAATAGAGAATT  1269
             ||||||||||||||||||||||||||||||||||||||||||||||||||||||||||||
Sbjct  1211  ATACACAGTTCACAGCAGTAGGTAAAGAGTTCAACCACCTGGAAAAAAGAATAGAGAATT  1270

Query  1270  TAAATAAAAAAGTTGATGATGGTTTCCTGGACATTTGGACTTACAATGCCGAACTGTTGG  1329
             ||||||||||||||||||||||||||||||||||||||||||||||||||||||||||||
Sbjct  1271  TAAATAAAAAAGTTGATGATGGTTTCCTGGACATTTGGACTTACAATGCCGAACTGTTGG  1330

Query  1330  TTCTATTGGAAAATGAAAGAACTTTGGACTACCACGATTCAAATGTGAAGAACTTATATG  1389
             ||||||||||||||||||||||||||||||||||||||||||||||||||||||||||||
Sbjct  1331  TTCTATTGGAAAATGAAAGAACTTTGGACTACCACGATTCAAATGTGAAGAACTTATATG  1390

Query  1390  AAAAGGTAAGAAGCCAGCTAAAAAACAATGCCAAGGAAATTGGAAACGGCTGCTTTGAAT  1449
             ||||||||||||||||||||||||||||||||||||||||||||||||||||||||||||
Sbjct  1391  AAAAGGTAAGAAGCCAGCTAAAAAACAATGCCAAGGAAATTGGAAACGGCTGCTTTGAAT  1450

Query  1450  TTTACCACAAATGCGATAACACGTGCATGGAAAGTGTCAAAAATGGGACTTATGACTACC  1509
             ||||||||||||||||||||||||||||||||||||||||||||||||||||||||||||
Sbjct  1451  TTTACCACAAATGCGATAACACGTGCATGGAAAGTGTCAAAAATGGGACTTATGACTACC  1510
Query  1510  CAAAATACTCAGAGGAAGCAAAATTAAACAGAGAAGAAATAGATGGGGTAAAGCTGGAAT  1569
             ||||||||||||||||||||||||||||||||||||||||||||||||||||||||||||
Sbjct  1511  CAAAATACTCAGAGGAAGCAAAATTAAACAGAGAAGAAATAGATGGGGTAAAGCTGGAAT  1570

Query  1570  CAACAAGGATTTACCAGATTTTGGCGATCTATTCAACTGTCGCCAGTTCATTGGTACTGG  1629
             ||||||||||||||||||||||||||||||||||||||||||||||||||||||||||||
Sbjct  1571  CAACAAGGATTTACCAGATTTTGGCGATCTATTCAACTGTCGCCAGTTCATTGGTACTGG  1630
```

```
Query  1630   TAGTCTCCCTGGGGGCAATCAGTTTCTGGATGTGCTCTAATGGGTCTCTACAGTGTAGAA  1689
              ||||||||||||||||||||||||||||||||||||||||||||||||||||||||||||
Sbjct  1631   TAGTCTCCCTGGGGGCAATCAGTTTCTGGATGTGCTCTAATGGGTCTCTACAGTGTAGAA  1690

Query  1690   TATGTATT  1697
              ||||||||
Sbjct  1691   TATGTATT  1698
```

5. Nucleoprotein (NP)

```
Identities = 1494/1497 (99%), Gaps = 0/1497 (0%)
Query  45     ATGGCGTCCCAAGGCACCAAACGATCATATGAACAAATGGAGACTGGTGGGGAGCGCCAG  104
              |||||||| |||||||||||||||||||||||||||||||||||||||||||||||||||
Sbjct  1      ATGGCGTCTCAAGGCACCAAACGATCATATGAACAAATGGAGACTGGTGGGGAGCGCCAG  60

Query  105    GATGCCACAGAAATCAGAGCATCTGTCGGAAGAATGATTGGTGGAATCGGGAGATTCTAC  164
              ||||||||||||||||||||||||||||||||||||||||||||||||||||||||||||
Sbjct  61     GATGCCACAGAAATCAGAGCATCTGTCGGAAGAATGATTGGTGGAATCGGGAGATTCTAC  120

Query  165    ATCCAAATGTGCACTGAACTCAAACTCAGTGATTATGATGGACGACTAATCCAGAATAGC  224
              ||||||||||||||||||||||||||||||||||||||||||||||||||||||||||||
Sbjct  121    ATCCAAATGTGCACTGAACTCAAACTCAGTGATTATGATGGACGACTAATCCAGAATAGC  180

Query  225    ATAACAATAGAGAGGATGGTGCTTTCTGCTTTTGATGAGAGAAGAAATAAATACCTAGAA  284
              ||||||||||||||||||||||||||||||||||||||||||||||||||||||||||||
Sbjct  181    ATAACAATAGAGAGGATGGTGCTTTCTGCTTTTGATGAGAGAAGAAATAAATACCTAGAA  240

Query  285    GAGCATCCCAGTGCTGGGAAGGACCCTAAGAAAACAGGAGGACCCATATATAGAAGAGTA  344
              ||||||||||||||||||||||||||||||||||||||||||||||||||||||||||||
Sbjct  241    GAGCATCCCAGTGCTGGGAAGGACCCTAAGAAAACAGGAGGACCCATATATAGAAGAGTA  300

Query  345    GACGGAAAGTGGATGAGAGAACTCATCCTTTATGACAAAGAAGAAATAAGGAGAGTTTGG  404
              |||||||||||||||||||||||||||||||||||||||| |||||||||||||||||||
Sbjct  301    GACGGAAAGTGGATGAGAGAACTCATCCTTTATGACAAAGRAGAAATAAGGAGAGTTTGG  360

Query  405    CGCCAAGCAAACAATGGCGAAGATGCAACAGCAGGTCTTACTCATATCATGATTTGGCAT  464
              |||| |||||||||||||||||||||||||||||||||||||||||||||||||||||||
Sbjct  361    CGCCTAGCAAACAATGGCGAAGATGCAACAGCAGGTCTTACTCATATCATGATTTGGCAT  420

Query  465    TCCAACCTGAATGATGCCACATATCAGAGAACAAGAGCGCTTGTTCGCACCGGAATGGAT  524
              ||||||||||||||||||||||||||||||||||||||||||||||||||||||||||||
Sbjct  421    TCCAACCTGAATGATGCCACATATCAGAGAACAAGAGCGCTTGTTCGCACCGGAATGGAT  480

Query  525    CCCAGAATGTGCTCTCTAATGCAAGGTTCAACACTTCCCAGAAGGTCTGGTGCCGCAGGT  584
              ||||||||||||||||||||||||||||||||||||||||||||||||||||||||||||
Sbjct  481    CCCAGAATGTGCTCTCTAATGCAAGGTTCAACACTTCCCAGAAGGTCTGGTGCCGCAGGT  540

Query  585    GCTGCGGTGAAAGGAGTTGGAACAATAGCAATGGAGTTAATCAGAATGATCAAACGTGGA  644
              ||||||||||||||||||||||||||||||||||||||||||||||||||||||||||||
Sbjct  541    GCTGCGGTGAAAGGAGTTGGAACAATAGCAATGGAGTTAATCAGAATGATCAAACGTGGA  600

Query  645    ATCAATGACCGAAATTTCTGGAGGGGTGAAAATGGACGAAGGACAAGGGTTGCTTATGAA  704
              ||||||||||||||||||||||||||||||||||||||||||||||||||||||||||||
Sbjct  601    ATCAATGACCGAAATTTCTGGAGGGGTGAAAATGGACGAAGGACAAGGGTTGCTTATGAA  660

Query  705    AGAATGTGCAATATCCTCAAAGGAAAATTTCAAACAGCTGCCCAGAGGGCAATGATGGAT  764
              ||||||||||||||||||||||||||||||||||||||||||||||||||||||||||||
Sbjct  661    AGAATGTGCAATATCCTCAAAGGAAAATTTCAAACAGCTGCCCAGAGGGCAATGATGGAT  720

Query  765    CAAGTAAGAGAAAGTCGAAACCCAGGAAACGCTGAGATTGAAGACCTCATTTTCCTGGCA  824
              ||||||||||||||||||||||||||||||||||||||||||||||||||||||||||||
Sbjct  721    CAAGTAAGAGAAAGTCGAAACCCAGGAAACGCTGAGATTGAAGACCTCATTTTCCTGGCA  780

Query  825    CGGTCAGCACTCATTCTGAGGGGATCAGTTGCACATAAATCCTGCCTGCCTGCTTGTGTG  884
              ||||||||||||||||||||||||||||||||||||||||||||||||||||||||||||
Sbjct  781    CGGTCAGCACTCATTCTGAGGGGATCAGTTGCACATAAATCCTGCCTGCCTGCTTGTGTG  840

Query  885    TATGGGCTTGCAGTAGCAAGTGGGCATGACTTTGAAAGGGAAGGGTACTCACTGGTCGGG  944
              ||||||||||||||||||||||||||||||||||||||||||||||||||||||||||||
```

```
Sbjct  841    TATGGGCTTGCAGTAGCAAGTGGGCATGACTTTGAAAGGGAAGGGTACTCACTGGTCGGG  900

Query  945    ATAGACCCATTCAAATTACTCCAAAACAGCCAAGTGGTCAGCCTGATGAGACCAAATGAA  1004
              ||||||||||||||||||||||||||||||||||||||||||||||||||||||||||||
Sbjct  901    ATAGACCCATTCAAATTACTCCAAAACAGCCAAGTGGTCAGCCTGATGAGACCAAATGAA  960

Query  1005   AACCCAGCTCACAAGAGTCAATTGGTGTGGATGGCATGCCACTCTGCTGCATTTGAAGAT  1064
              ||||||||||||||||||||||||||||||||||||||||||||||||||||||||||||
Sbjct  961    AACCCAGCTCACAAGAGTCAATTGGTGTGGATGGCATGCCACTCTGCTGCATTTGAAGAT  1020

Query  1065   TTAAGAGTATCAAGTTTCATAAGAGGAAAGAAAGTGATTCCAAGAGGAAAGCTTTCCACA  1124
              ||||||||||||||||||||||||||||||||||||||||||||||||||||||||||||
Sbjct  1021   TTAAGAGTATCAAGTTTCATAAGAGGAAAGAAAGTGATTCCAAGAGGAAAGCTTTCCACA  1080

Query  1125   AGAGGGGTCCAGATTGCTTCAAATGAGAATGTGGAAACCATGGACTCCAATACCCTGGAA  1184
              ||||||||||||||||||||||||||||||||||||||||||||||||||||||||||||
Sbjct  1081   AGAGGGGTCCAGATTGCTTCAAATGAGAATGTGGAAACCATGGACTCCAATACCCTGGAA  1140

Query  1185   CTGAGAAGCAGATACTGGGCCATAAGGACCAGGAGTGGAGGAAATACCAATCAACAAAAG  1244
              ||||||||||||||||||||||||||||||||||||||||||||||||||||||||||||
Sbjct  1141   CTGAGAAGCAGATACTGGGCCATAAGGACCAGGAGTGGAGGAAATACCAATCAACAAAAG  1200

Query  1245   GCATCCGCAGGCCAGATCAGTGTGCAGCCTACATTCTCAGTGCAGCGGAATCTCCCTTTT  1304
              ||||||||||||||||||||||||||||||||||||||||||||||||||||||||||||
Sbjct  1201   GCATCCGCAGGCCAGATCAGTGTGCAGCCTACATTCTCAGTGCAGCGGAATCTCCCTTTT  1260

Query  1305   GAAAGAGCAACCGTTATGGCAGCATTCAGCGGGAACAATGAAGGACGGACATCCGACATG  1364
              ||||||||||||||||||||||||||||||||||||||||||||||||||||||||||||
Sbjct  1261   GAAAGAGCAACCGTTATGGCAGCATTCAGCGGGAACAATGAAGGACGGACATCCGACATG  1320

Query  1365   CGAACAGAAGTTATAAGAATGATGGAAAGTGCAAAGCCAGAAGATTTGTCCTTCCAGGGG  1424
              ||||||||||||||||||||||||||||||||||||||||||||||||||||||||||||
Sbjct  1321   CGAACAGAAGTTATAAGAATGATGGAAAGTGCAAAGCCAGAAGATTTGTCCTTCCAGGGG  1380

Query  1425   CGGGGAGTCTTCGAGCTCTCGGACGAAAAGGCAACGAACCCGATCGTGCCTTCCTTTGAC  1484
              ||||||||||||||||||||||||||||||||||||||||||||||||||||||||||||
Sbjct  1381   CGGGGAGTCTTCGAGCTCTCGGACGAAAAGGCAACGAACCCGATCGTGCCTTCCTTTGAC  1440

Query  1485   ATGAGTAATGAAGGGTCTTATTTCTTCGGAGACAATGCAGAGGAGTATGACAGTTGA  1541
              |||||||||||||||||||||||||||||||||||||||||||||||||||||||||
Sbjct  1441   ATGAGTAATGAAGGGTCTTATTTCTTCGGAGACAATGCAGAGGAGTATGACAGTTGA  1497
```

6. Neuraminidase (NA)

```
    Identities = 1399/1399 (100%), Gaps = 0/1399 (0%)
Query  11     CCAAAAGATAATAACCATTGGTTCGGTCTGTATGACAATTGGAATGGCTAACTTAATATT  70
              ||||||||||||||||||||||||||||||||||||||||||||||||||||||||||||
Sbjct  12     CCAAAAGATAATAACCATTGGTTCGGTCTGTATGACAATTGGAATGGCTAACTTAATATT  71

Query  71     ACAAATTGGAAACATAATCTCAATATGGATTAGCCACTCAATTCAACTTGGGAATCAAAA  130
              ||||||||||||||||||||||||||||||||||||||||||||||||||||||||||||
Sbjct  72     ACAAATTGGAAACATAATCTCAATATGGATTAGCCACTCAATTCAACTTGGGAATCAAAA  131

Query  131    TCAGATTGAAACATGCAATCAAAGCGTCATTACTTATGAAAACAACACTTGGGTAAATCA  190
              ||||||||||||||||||||||||||||||||||||||||||||||||||||||||||||
Sbjct  132    TCAGATTGAAACATGCAATCAAAGCGTCATTACTTATGAAAACAACACTTGGGTAAATCA  191

Query  191    GACATATGTTAACATCAGCAACACCAACTTTGCTGCTGGACAGTCAGTGGTTTCCGTGAA  250
              ||||||||||||||||||||||||||||||||||||||||||||||||||||||||||||
Sbjct  192    GACATATGTTAACATCAGCAACACCAACTTTGCTGCTGGACAGTCAGTGGTTTCCGTGAA  251

Query  251    ATTAGCGGGCAATTCCTCTCTCTGCCCTGTTAGTGGATGGGCTATATACAGTAAAGACAA  310
              ||||||||||||||||||||||||||||||||||||||||||||||||||||||||||||
Sbjct  252    ATTAGCGGGCAATTCCTCTCTCTGCCCTGTTAGTGGATGGGCTATATACAGTAAAGACAA  311

Query  311    CAGTGTAAGAATCGGTTCCAAGGGGGATGTGTTTGTCATAAGGGAACCATTCATATCATG  370
              ||||||||||||||||||||||||||||||||||||||||||||||||||||||||||||
Sbjct  312    CAGTGTAAGAATCGGTTCCAAGGGGGATGTGTTTGTCATAAGGGAACCATTCATATCATG  371

Query  371    CTCCCCCTTGGAATGCAGAACCTTCTTCTTGACTCAAGGGGCCTTGCTAAATGACAAACA  430
              ||||||||||||||||||||||||||||||||||||||||||||||||||||||||||||
```

```
Sbjct  372   CTCCCCCTTGGAATGCAGAACCTTCTTCTTGACTCAAGGGGCCTTGCTAAATGACAAACA  431

Query  431   TTCCAATGGAACCATTAAAGACAGGAGCCCATATCGAACCCTAATGAGCTGTCCTATTGG  490
             ||||||||||||||||||||||||||||||||||||||||||||||||||||||||||||
Sbjct  432   TTCCAATGGAACCATTAAAGACAGGAGCCCATATCGAACCCTAATGAGCTGTCCTATTGG  491

Query  491   TGAAGTTCCCTCTCCATACAACTCAAGATTTGAGTCAGTCGCTTGGTCAGCAAGTGCTTG  550
             ||||||||||||||||||||||||||||||||||||||||||||||||||||||||||||
Sbjct  492   TGAAGTTCCCTCTCCATACAACTCAAGATTTGAGTCAGTCGCTTGGTCAGCAAGTGCTTG  551

Query  551   TCATGATGGCATCAATTGGCTAACAATTGGAATTTCTGGCCCAGACAATGGGGCAGTGGC  610
             ||||||||||||||||||||||||||||||||||||||||||||||||||||||||||||
Sbjct  552   TCATGATGGCATCAATTGGCTAACAATTGGAATTTCTGGCCCAGACAATGGGGCAGTGGC  611

Query  611   TGTGTTAAAGTACAACGGCATAATAACAGACACTATCAAGAGTTGGAGAAACAATATATT  670
             ||||||||||||||||||||||||||||||||||||||||||||||||||||||||||||
Sbjct  612   TGTGTTAAAGTACAACGGCATAATAACAGACACTATCAAGAGTTGGAGAAACAATATATT  671

Query  671   GAGAACACAAGAGTCTGAATGTGCATGTGTAAATGGTTCTTGCTTTACTGTAATGACCGA  730
             ||||||||||||||||||||||||||||||||||||||||||||||||||||||||||||
Sbjct  672   GAGAACACAAGAGTCTGAATGTGCATGTGTAAATGGTTCTTGCTTTACTGTAATGACCGA  731

Query  731   TGGACCAAGTAATGGACAGGCCTCATACAAGATCTTCAGAATAGAAAAGGGAAAGATAGT  790
             ||||||||||||||||||||||||||||||||||||||||||||||||||||||||||||
Sbjct  732   TGGACCAAGTAATGGACAGGCCTCATACAAGATCTTCAGAATAGAAAAGGGAAAGATAGT  791

Query  791   CAAATCAGTCGAAATGAATGCCCCTAATTATCACTATGAGGAATGCTCCTGTTATCCTGA  850
             ||||||||||||||||||||||||||||||||||||||||||||||||||||||||||||
Sbjct  792   CAAATCAGTCGAAATGAATGCCCCTAATTATCACTATGAGGAATGCTCCTGTTATCCTGA  851

Query  851   TTCTAGTGAAATCACATGTGTGTGCAGGGATAACTGGCATGGCTCGAATCGACCGTGGGT  910
             ||||||||||||||||||||||||||||||||||||||||||||||||||||||||||||
Sbjct  852   TTCTAGTGAAATCACATGTGTGTGCAGGGATAACTGGCATGGCTCGAATCGACCGTGGGT  911

Query  911   GTCTTTCAACCAGAATCTGGAATATCAGATAGGATACATATGCAGTGGGATTTTCGGAGA  970
             ||||||||||||||||||||||||||||||||||||||||||||||||||||||||||||
Sbjct  912   GTCTTTCAACCAGAATCTGGAATATCAGATAGGATACATATGCAGTGGGATTTTCGGAGA  971

Query  971   CAATCCACGCCCTAATGATAAGACAGGCAGTTGTGGTCCAGTATCGTCTAATGGAGCAAA  1030
             ||||||||||||||||||||||||||||||||||||||||||||||||||||||||||||
Sbjct  972   CAATCCACGCCCTAATGATAAGACAGGCAGTTGTGGTCCAGTATCGTCTAATGGAGCAAA  1031

Query  1031  TGGAGTAAAAGGGTTTTCATTCAAATACGGCAATGGTGTTTGGATAGGGAGAACTAAAAG  1090
             ||||||||||||||||||||||||||||||||||||||||||||||||||||||||||||
Sbjct  1032  TGGAGTAAAAGGGTTTTCATTCAAATACGGCAATGGTGTTTGGATAGGGAGAACTAAAAG  1091

Query  1091  CATTAGTTCAAGAAACGGTTTTGAGATGATTTGGGATCCGAACGGATGGACTGGGACAGA  1150
             ||||||||||||||||||||||||||||||||||||||||||||||||||||||||||||
Sbjct  1092  CATTAGTTCAAGAAACGGTTTTGAGATGATTTGGGATCCGAACGGATGGACTGGGACAGA  1151

Query  1151  CAATAACTTCTCAATAAAGCAAGATATCGTAGGAATAAATGAGTGGTCAGGATATAGCGG  1210
             ||||||||||||||||||||||||||||||||||||||||||||||||||||||||||||
Sbjct  1152  CAATAACTTCTCAATAAAGCAAGATATCGTAGGAATAAATGAGTGGTCAGGATATAGCGG  1211

Query  1211  GAGTTTTGTTCAGCATCCAGAACTAACAGGGCTGGATTGTATAAGACCTTGCTTCTGGGT  1270
             ||||||||||||||||||||||||||||||||||||||||||||||||||||||||||||
Sbjct  1212  GAGTTTTGTTCAGCATCCAGAACTAACAGGGCTGGATTGTATAAGACCTTGCTTCTGGGT  1271

Query  1271  TGAACTAATCAGAGGGCGACCCAAAGAGAACACAATCTGGACTAGCGGGAGCAGCATATC  1330
             ||||||||||||||||||||||||||||||||||||||||||||||||||||||||||||
Sbjct  1272  TGAACTAATCAGAGGGCGACCCAAAGAGAACACAATCTGGACTAGCGGGAGCAGCATATC  1331

Query  1331  CTTTTGTGGTGTAAACAGTGACACTGTGGGTTGGTCTTGGCCAGACGGTGCTGAGTTGCC  1390
             ||||||||||||||||||||||||||||||||||||||||||||||||||||||||||||
Sbjct  1332  CTTTTGTGGTGTAAACAGTGACACTGTGGGTTGGTCTTGGCCAGACGGTGCTGAGTTGCC  1391

Query  1391  ATTTACCATTGACAAGTAA  1409
             |||||||||||||||||||
Sbjct  1392  ATTTACCATTGACAAGTAA  1410
```

7. Matrix (M)

```
Identities = 855/855 (100%), Gaps = 0/855 (0%)

Query  26   ATGGACTCCAACACCATGTCAAGCTTTCAGGTAGACTGTTTCCTTTGGCATATCCGCAAG  85
            ||||||||||||||||||||||||||||||||||||||||||||||||||||||||||||
Sbjct  1    ATGGACTCCAACACCATGTCAAGCTTTCAGGTAGACTGTTTCCTTTGGCATATCCGCAAG  60

Query  86   CGATTTGCAGACAATGGATTGGGTGATGCCCCATTCCTTGATCGGCTCCGCCGAGATCAA  145
            ||||||||||||||||||||||||||||||||||||||||||||||||||||||||||||
Sbjct  61   CGATTTGCAGACAATGGATTGGGTGATGCCCCATTCCTTGATCGGCTCCGCCGAGATCAA  120

Query  146  AAGTCCTTAAAAGGAAGAGGCAACACCCTTGGCCTCGATATCGAAACAGCCACTCTTGTT  205
            ||||||||||||||||||||||||||||||||||||||||||||||||||||||||||||
Sbjct  121  AAGTCCTTAAAAGGAAGAGGCAACACCCTTGGCCTCGATATCGAAACAGCCACTCTTGTT  180

Query  206  GGGAAACAAATCGTGGAATGGATCTTGAAAGAGGAATCCAGCGAGACACTTAGAATGACA  265
            ||||||||||||||||||||||||||||||||||||||||||||||||||||||||||||
Sbjct  181  GGGAAACAAATCGTGGAATGGATCTTGAAAGAGGAATCCAGCGAGACACTTAGAATGACA  240

Query  266  ATTGCATCTGTACCTACTTCGCGCTACCTTTCTGACATGACCCTCGAGGAAATGTCACGA  325
            ||||||||||||||||||||||||||||||||||||||||||||||||||||||||||||
Sbjct  241  ATTGCATCTGTACCTACTTCGCGCTACCTTTCTGACATGACCCTCGAGGAAATGTCACGA  300

Query  326  GACTGGTTCATGCTCATGCCTAGGCAAAAGATAATAGGCCCTCTTTGCGTGCGATTGGAC  385
            ||||||||||||||||||||||||||||||||||||||||||||||||||||||||||||
Sbjct  301  GACTGGTTCATGCTCATGCCTAGGCAAAAGATAATAGGCCCTCTTTGCGTGCGATTGGAC  360

Query  386  CAGGCGATCATGGAAAAGAACATAGTACTGAAAGCGAACTTCAGTGTAATCTTTAACCGA  445
            ||||||||||||||||||||||||||||||||||||||||||||||||||||||||||||
Sbjct  361  CAGGCGATCATGGAAAAGAACATAGTACTGAAAGCGAACTTCAGTGTAATCTTTAACCGA  420

Query  446  TTAGAGACCTTGATACTACTAAGGGCTTTCACTGAGGAGGGAGCAATAGTTGGAGAAATT  505
            ||||||||||||||||||||||||||||||||||||||||||||||||||||||||||||
Sbjct  421  TTAGAGACCTTGATACTACTAAGGGCTTTCACTGAGGAGGGAGCAATAGTTGGAGAAATT  480

Query  506  TCACCATTACCTTCTCTTCCAGGACATACTTATGAGGATGTCAAAAATGCAGTTGGGGTC  565
            ||||||||||||||||||||||||||||||||||||||||||||||||||||||||||||
Sbjct  481  TCACCATTACCTTCTCTTCCAGGACATACTTATGAGGATGTCAAAAATGCAGTTGGGGTC  540

Query  566  CTCATCGGAGGACTTGAATGGAATGGTAACACGGTTCGAGTCTCTGAAAATATACAGAGA  625
            ||||||||||||||||||||||||||||||||||||||||||||||||||||||||||||
Sbjct  541  CTCATCGGAGGACTTGAATGGAATGGTAACACGGTTCGAGTCTCTGAAAATATACAGAGA  600

Query  626  TTCGCTTGGAGAAACTGTGATGAGAATGGGAGACCTTCACTACCTCCAGAGCAGAAATGA  685
            ||||||||||||||||||||||||||||||||||||||||||||||||||||||||||||
Sbjct  601  TTCGCTTGGAGAAACTGTGATGAGAATGGGAGACCTTCACTACCTCCAGAGCAGAAATGA  660

Query  686  AAAGTGGCGAGAGCAATTGGGACAGAAATTTGAGGAAATAAGGTGGTTAATTGAAGAAAT  745
            ||||||||||||||||||||||||||||||||||||||||||||||||||||||||||||
Sbjct  661  AAAGTGGCGAGAGCAATTGGGACAGAAATTTGAGGAAATAAGGTGGTTAATTGAAGAAAT  720

Query  746  GCGGCACAGATTGAAAGCGACAGAGAATAGTTTCGAACAAATAACATTTATGCAAGCCTT  805
            ||||||||||||||||||||||||||||||||||||||||||||||||||||||||||||
Sbjct  721  GCGGCACAGATTGAAAGCGACAGAGAATAGTTTCGAACAAATAACATTTATGCAAGCCTT  780

Query  806  ACAACTACTGCTTGAAGTAGAACAAGAGATAAGAGCTTTCTCGTTTCAGCTTATTTAATG  865
            ||||||||||||||||||||||||||||||||||||||||||||||||||||||||||||
Sbjct  781  ACAACTACTGCTTGAAGTAGAACAAGAGATAAGAGCTTTCTCGTTTCAGCTTATTTAATG  840

Query  866  ATAAAAAACACCCTT  880
            |||||||||||||||
Sbjct  841  ATAAAAAACACCCTT  855
```

8. Nonstructural protein (NS)

```
Identities = 855/855 (100%), Gaps = 0/855 (0%)



Query  26   ATGGACTCCAACACCATGTCAAGCTTTCAGGTAGACTGTTTCCTTTGGCATATCCGCAAG  85
```

```
                ||||||||||||||||||||||||||||||||||||||||||||||||||||||||||||
Sbjct  1    ATGGACTCCAACACCATGTCAAGCTTTCAGGTAGACTGTTTCCTTTGGCATATCCGCAAG  60

Query  86   CGATTTGCAGACAATGGATTGGGTGATGCCCCATTCCTTGATCGGCTCCGCCGAGATCAA  145
            ||||||||||||||||||||||||||||||||||||||||||||||||||||||||||||
Sbjct  61   CGATTTGCAGACAATGGATTGGGTGATGCCCCATTCCTTGATCGGCTCCGCCGAGATCAA  120

Query  146  AAGTCCTTAAAAGGAAGAGGCAACACCCTTGGCCTCGATATCGAAACAGCCACTCTTGTT  205
            ||||||||||||||||||||||||||||||||||||||||||||||||||||||||||||
Sbjct  121  AAGTCCTTAAAAGGAAGAGGCAACACCCTTGGCCTCGATATCGAAACAGCCACTCTTGTT  180

Query  206  GGGAAACAAATCGTGGAATGGATCTTGAAAGAGGAATCCAGCGAGACACTTAGAATGACA  265
            ||||||||||||||||||||||||||||||||||||||||||||||||||||||||||||
Sbjct  181  GGGAAACAAATCGTGGAATGGATCTTGAAAGAGGAATCCAGCGAGACACTTAGAATGACA  240

Query  266  ATTGCATCTGTACCTACTTCGCGCTACCTTTCTGACATGACCCTCGAGGAAATGTCACGA  325
            ||||||||||||||||||||||||||||||||||||||||||||||||||||||||||||
Sbjct  241  ATTGCATCTGTACCTACTTCGCGCTACCTTTCTGACATGACCCTCGAGGAAATGTCACGA  300

Query  326  GACTGGTTCATGCTCATGCCTAGGCAAAAGATAATAGGCCCTCTTTGCGTGCGATTGGAC  385
            ||||||||||||||||||||||||||||||||||||||||||||||||||||||||||||
Sbjct  301  GACTGGTTCATGCTCATGCCTAGGCAAAAGATAATAGGCCCTCTTTGCGTGCGATTGGAC  360

Query  386  CAGGCGATCATGGAAAAGAACATAGTACTGAAAGCGAACTTCAGTGTAATCTTTAACCGA  445
            ||||||||||||||||||||||||||||||||||||||||||||||||||||||||||||
Sbjct  361  CAGGCGATCATGGAAAAGAACATAGTACTGAAAGCGAACTTCAGTGTAATCTTTAACCGA  420

Query  446  TTAGAGACCTTGATACTACTAAGGGCTTTCACTGAGGAGGGAGCAATAGTTGGAGAAATT  505
            ||||||||||||||||||||||||||||||||||||||||||||||||||||||||||||
Sbjct  421  TTAGAGACCTTGATACTACTAAGGGCTTTCACTGAGGAGGGAGCAATAGTTGGAGAAATT  480

Query  506  TCACCATTACCTTCTCTTCCAGGACATACTTATGAGGATGTCAAAAATGCAGTTGGGGTC  565
            ||||||||||||||||||||||||||||||||||||||||||||||||||||||||||||
Sbjct  481  TCACCATTACCTTCTCTTCCAGGACATACTTATGAGGATGTCAAAAATGCAGTTGGGGTC  540

Query  566  CTCATCGGAGGACTTGAATGGAATGGTAACACGGTTCGAGTCTCTGAAAATATACAGAGA  625
            ||||||||||||||||||||||||||||||||||||||||||||||||||||||||||||
Sbjct  541  CTCATCGGAGGACTTGAATGGAATGGTAACACGGTTCGAGTCTCTGAAAATATACAGAGA  600

Query  626  TTCGCTTGGAGAAACTGTGATGAGAATGGGAGACCTTCACTACCTCCAGAGCAGAAATGA  685
            ||||||||||||||||||||||||||||||||||||||||||||||||||||||||||||
Sbjct  601  TTCGCTTGGAGAAACTGTGATGAGAATGGGAGACCTTCACTACCTCCAGAGCAGAAATGA  660

Query  686  AAAGTGGCGAGAGCAATTGGGACAGAAATTTGAGGAAATAAGGTGGTTAATTGAAGAAAT  745
            ||||||||||||||||||||||||||||||||||||||||||||||||||||||||||||
Sbjct  661  AAAGTGGCGAGAGCAATTGGGACAGAAATTTGAGGAAATAAGGTGGTTAATTGAAGAAAT  720

Query  746  GCGGCACAGATTGAAAGCGACAGAGAATAGTTTCGAACAAATAACATTTATGCAAGCCTT  805
            ||||||||||||||||||||||||||||||||||||||||||||||||||||||||||||
Sbjct  721  GCGGCACAGATTGAAAGCGACAGAGAATAGTTTCGAACAAATAACATTTATGCAAGCCTT  780

Query  806  ACAACTACTGCTTGAAGTAGAACAAGAGATAAGAGCTTTCTCGTTTCAGCTTATTTAATG  865
            ||||||||||||||||||||||||||||||||||||||||||||||||||||||||||||
Sbjct  781  ACAACTACTGCTTGAAGTAGAACAAGAGATAAGAGCTTTCTCGTTTCAGCTTATTTAATG  840

Query  866  ATAAAAAACACCCTT  880
            |||||||||||||||
Sbjct  841  ATAAAAAACACCCTT  855
```

The reference found for SRR27490310 and the reference for Influenza A virus (A/California/07/2009(H1N1)) GCF_001343785.1.

1. Basic polymerase 2 (PB2)

```
Identities = 2279/2280 (99%), Gaps = 0/2280 (0%)

Query  27    ATGGAGAGAATAAAAGAACTGAGAGATCTAATGTCGCAGTCCCGCACTCGCGAGATACTC  86
             ||||||||||||||||||||||||||||||||||||||||||||||||||||||||||||
Sbjct  1     ATGGAGAGAATAAAAGAACTGAGAGATCTAATGTCGCAGTCCCGCACTCGCGAGATACTC  60
```

```
Query  87    ACTAAGACCACTGTGGACCATATGGCCATAATCAAAAAGTACACATCAGGAAGGCAAGAG  146
             ||||||||||||||||||||||||||||||||||||||||||||||||||||||||||||
Sbjct  61    ACTAAGACCACTGTGGACCATATGGCCATAATCAAAAAGTACACATCAGGAAGGCAAGAG  120

Query  147   AAGAACCCCGCACTCAGAATGAAGTGGATGATGGCAATGAGATACCCAATTACAGCAGAC  206
             ||||||||||||||||||||||||||||||||||||||||||||||||||||||||||||
Sbjct  121   AAGAACCCCGCACTCAGAATGAAGTGGATGATGGCAATGAGATACCCAATTACAGCAGAC  180

Query  207   AAGAGAATAATGGACATGATTCCAGAGAGGAATGAACAAGGACAAACCCTCTGGAGCAAA  266
             ||||||||||||||||||||||||||||||||||||||||||||||||||||||||||||
Sbjct  181   AAGAGAATAATGGACATGATTCCAGAGAGGAATGAACAAGGACAAACCCTCTGGAGCAAA  240

Query  267   ACAAACGATGCTGGATCAGACCGAGTGATGGTATCACCTCTGGCCGTAACATGGTGGAAT  326
             ||||||||||||||||||||||||||||||||||||||||||||||||||||||||||||
Sbjct  241   ACAAACGATGCTGGATCAGACCGAGTGATGGTATCACCTCTGGCCGTAACATGGTGGAAT  300

Query  327   AGGAATGGCCCAACAACAAGTACAGTTCATTACCCTAAGGTATATAAAACTTATTTCGAA  386
             ||||||||||||||||||||||||||||||||||||||||||||||||||||||||||||
Sbjct  301   AGGAATGGCCCAACAACAAGTACAGTTCATTACCCTAAGGTATATAAAACTTATTTCGAA  360

Query  387   AAGGTCGAAAGGTTGAAACATGGTACCTTCGGCCCTGTCCACTTCAGAAATCAAGTTAAA  446
             ||||||||||||||||||||||||||||||||||||||||||||||||||||||||||||
Sbjct  361   AAGGTCGAAAGGTTGAAACATGGTACCTTCGGCCCTGTCCACTTCAGAAATCAAGTTAAA  420

Query  447   ATAAGGAGGAGAGTTGATACAAACCCTGGCCATGCAGATCTCAGTGCCAAGGAGGCACAG  506
             ||||||||||||||||||||||||||||||||||||||||||||||||||||||||||||
Sbjct  421   ATAAGGAGGAGAGTTGATACAAACCCTGGCCATGCAGATCTCAGTGCCAAGGAGGCACAG  480

Query  507   GATGTGATTATGGAAGTTGTTTTCCCAAATGAAGTGGGGGCAAGAATACTGACATCAGAG  566
             ||||||||||||||||||||||||||||||||||||||||||||||||||||||||||||
Sbjct  481   GATGTGATTATGGAAGTTGTTTTCCCAAATGAAGTGGGGGCAAGAATACTGACATCAGAG  540

Query  567   TCACAGCTGGCAATAACAAAAGAGAAGAAAGAAGAGCTCCAGGATTGTAAAATTGCTCCC  626
             ||||||||||||||||||||||||||||||||||||||||||||||||||||||||||||
Sbjct  541   TCACAGCTGGCAATAACAAAAGAGAAGAAAGAAGAGCTCCAGGATTGTAAAATTGCTCCC  600

Query  627   TTGATGGTGGCGTACATGCTAGAAAGAGAATTGGTCCGTAAAACAAGGTTTCTCCCAGTA  686
             ||||||||||||||||||||||||||||||||||||||||||||||||||||||||||||
Sbjct  601   TTGATGGTGGCGTACATGCTAGAAAGAGAATTGGTCCGTAAAACAAGGTTTCTCCCAGTA  660

Query  687   GCCGGCGGAACAGGCAGTGTTTATATTGAAGTGTTGCACTTAACCCAAGGGACGTGCTGG  746
             ||||||||||||||||||||||||||||||||||||||||||||||||||||||||||||
Sbjct  661   GCCGGCGGAACAGGCAGTGTTTATATTGAAGTGTTGCACTTAACCCAAGGGACGTGCTGG  720

Query  747   GAGCAGATGTACACTCCAGGAGGAGAAGTGAGAAATGATGATGTTGACCAAAGTTTGATT  806
             ||||||||||||||||||||||||||||||||||||||||||||||||||||||||||||
Sbjct  721   GAGCAGATGTACACTCCAGGAGGAGAAGTGAGAAATGATGATGTTGACCAAAGTTTGATT  780

Query  807   ATCGCTGCTAGAAACATAGTAAGAAGAGCAGCAGTGTCAGCAGACCCATTAGCATCTCTC  866
             ||||||||||||||||||||||||||||||||||||||||||||||||||||||||||||
Sbjct  781   ATCGCTGCTAGAAACATAGTAAGAAGAGCAGCAGTGTCAGCAGACCCATTAGCATCTCTC  840

Query  867   TTGGAAATGTGCCACAGCACACAGATTGGAGGAGTAAGGATGGTGGACATCCTTAGACAG  926
             ||||||||||||||||||||||||||||||||||||||||||||||||||||||||||||
Sbjct  841   TTGGAAATGTGCCACAGCACACAGATTGGAGGAGTAAGGATGGTGGACATCCTTAGACAG  900

Query  927   AATCCAACTGAGGAACAAGCCGTAGACATATGCAAGGCAGCAATAGGGTTGAGGATTAGC  986
             ||||||||||||||||||||||||||||||||||||||||||||||||||||||||||||
Sbjct  901   AATCCAACTGAGGAACAAGCCGTAGACATATGCAAGGCAGCAATAGGGTTGAGGATTAGC  960

Query  987   TCATCTTTCAGTTTTGGTGGGTTCACTTTCAAAAGGACAAGCGGATCATCAGTCAAGAAA  1046
             ||||||||||||||||||||||||||||||||||||||||||||||||||||||||||||
Sbjct  961   TCATCTTTCAGTTTTGGTGGGTTCACTTTCAAAAGGACAAGCGGATCATCAGTCAAGAAA  1020

Query  1047  GAAGAAGAAGTGCTAACGGGCAACCTCCAAACACTGAAAATAAGAGTACATGAAGGGTAT  1106
             ||||||||||||||||||||||||||||||||||||||||||||||||||||||||||||
Sbjct  1021  GAAGAAGAAGTGCTAACGGGCAACCTCCAAACACTGAAAATAAGAGTACATGAAGGGTAT  1080

Query  1107  GAAGAATTCACAATGGTTGGGAGAAGAGCAACAGCTATTCTCAGAAAGGCAACCAGGAGA  1166
             ||||||||||||||||||||||||||||||||||||||||||||||||||||||||||||
```

```
Sbjct  1081   GAAGAATTCACAATGGTTGGGAGAAGAGCAACAGCTATTCTCAGAAAGGCAACCAGGAGA  1140

Query  1167   TTGATCCAGTTGATAGTAAGCGGGAGAGATGAGCAGTCAATTGCTGAGGCAATAATTGTG  1226
              ||||||||||||||||||||||||||||| ||||||||||||||||||||||||||||||
Sbjct  1141   TTGATCCAGTTGATAGTAAGCGGGAGAGACGAGCAGTCAATTGCTGAGGCAATAATTGTG  1200

Query  1227   GCCATGGTATTCTCACAGGAGGATTGCATGATCAAGGCAGTTAGGGGCGATCTGAACTTT  1286
              ||||||||||||||||||||||||||||||||||||||||||||||||||||||||||||
Sbjct  1201   GCCATGGTATTCTCACAGGAGGATTGCATGATCAAGGCAGTTAGGGGCGATCTGAACTTT  1260

Query  1287   GTCAATAGGGCAAACCAGCGACTGAACCCCATGCACCAACTCTTGAGGCATTTCCAAAAA  1346
              ||||||||||||||||||||||||||||||||||||||||||||||||||||||||||||
Sbjct  1261   GTCAATAGGGCAAACCAGCGACTGAACCCCATGCACCAACTCTTGAGGCATTTCCAAAAA  1320

Query  1347   GATGCAAAAGTGCTTTTCCAGAACTGGGGAATTGAATCCATCGACAATGTGATGGGAATG  1406
              ||||||||||||||||||||||||||||||||||||||||||||||||||||||||||||
Sbjct  1321   GATGCAAAAGTGCTTTTCCAGAACTGGGGAATTGAATCCATCGACAATGTGATGGGAATG  1380

Query  1407   ATCGGAATACTGCCCGACATGACCCCAAGCACGGAGATGTCGCTGAGAGGGATAAGAGTC  1466
              ||||||||||||||||||||||||||||||||||||||||||||||||||||||||||||
Sbjct  1381   ATCGGAATACTGCCCGACATGACCCCAAGCACGGAGATGTCGCTGAGAGGGATAAGAGTC  1440

Query  1467   AGCAAAATGGGAGTAGATGAATACTCCAGCACGGAGAGAGTGGTAGTGAGTATTGACCGA  1526
              ||||||||||||||||||||||||||||||||||||||||||||||||||||||||||||
Sbjct  1441   AGCAAAATGGGAGTAGATGAATACTCCAGCACGGAGAGAGTGGTAGTGAGTATTGACCGA  1500

Query  1527   TTTTTAAGGGTTAGAGATCAAAGAGGGAACGTACTATTGTCTCCCGAAGAAGTCAGTGAA  1586
              ||||||||||||||||||||||||||||||||||||||||||||||||||||||||||||
Sbjct  1501   TTTTTAAGGGTTAGAGATCAAAGAGGGAACGTACTATTGTCTCCCGAAGAAGTCAGTGAA  1560

Query  1587   ACGCAAGGAACTGAGAAGTTGACAATAACTTATTCGTCATCAATGATGTGGGAGATCAAT  1646
              ||||||||||||||||||||||||||||||||||||||||||||||||||||||||||||
Sbjct  1561   ACGCAAGGAACTGAGAAGTTGACAATAACTTATTCGTCATCAATGATGTGGGAGATCAAT  1620

Query  1647   GGCCCTGAGTCAGTGCTAGTCAACACTTATCAATGGATAATCAGGAACTGGGAAATTGTG  1706
              ||||||||||||||||||||||||||||||||||||||||||||||||||||||||||||
Sbjct  1621   GGCCCTGAGTCAGTGCTAGTCAACACTTATCAATGGATAATCAGGAACTGGGAAATTGTG  1680

Query  1707   AAAATTCAATGGTCACAAGATCCCACAATGTTATACAACAAAATGGAATTTGAACCATTT  1766
              ||||||||||||||||||||||||||||||||||||||||||||||||||||||||||||
Sbjct  1681   AAAATTCAATGGTCACAAGATCCCACAATGTTATACAACAAAATGGAATTTGAACCATTT  1740

Query  1767   CAGTCTCTTGTCCCTAAGGCAACCAGAAGCCGGTACAGTGGATTCGTAAGGACACTGTTC  1826
              ||||||||||||||||||||||||||||||||||||||||||||||||||||||||||||
Sbjct  1741   CAGTCTCTTGTCCCTAAGGCAACCAGAAGCCGGTACAGTGGATTCGTAAGGACACTGTTC  1800

Query  1827   CAGCAAATGCGGGATGTGCTTGGGACATTTGACACTGTCCAAATAATAAAACTTCTCCCC  1886
              ||||||||||||||||||||||||||||||||||||||||||||||||||||||||||||
Sbjct  1801   CAGCAAATGCGGGATGTGCTTGGGACATTTGACACTGTCCAAATAATAAAACTTCTCCCC  1860

Query  1887   TTTGCTGCTGCCCCACCAGAACAGAGTAGGATGCAATTTTCCTCATTGACTGTGAATGTG  1946
              ||||||||||||||||||||||||||||||||||||||||||||||||||||||||||||
Sbjct  1861   TTTGCTGCTGCCCCACCAGAACAGAGTAGGATGCAATTTTCCTCATTGACTGTGAATGTG  1920

Query  1947   AGAGGATCAGGGTTGAGGATACTGGTAAGAGGCAATTCTCCAGTATTCAATTACAACAAG  2006
              ||||||||||||||||||||||||||||||||||||||||||||||||||||||||||||
Sbjct  1921   AGAGGATCAGGGTTGAGGATACTGGTAAGAGGCAATTCTCCAGTATTCAATTACAACAAG  1980

Query  2007   GCAACCAAACGACTTACAGTTCTTGGAAAGGATGCAGGTGCATTGACTGAAGATCCAGAT  2066
              ||||||||||||||||||||||||||||||||||||||||||||||||||||||||||||
Sbjct  1981   GCAACCAAACGACTTACAGTTCTTGGAAAGGATGCAGGTGCATTGACTGAAGATCCAGAT  2040

Query  2067   GAAGGCACATCTGGGGTGGAGTCTGCTGTCCTGAGAGGATTTCTCATTTTGGGCAAAGAA  2126
              ||||||||||||||||||||||||||||||||||||||||||||||||||||||||||||
Sbjct  2041   GAAGGCACATCTGGGGTGGAGTCTGCTGTCCTGAGAGGATTTCTCATTTTGGGCAAAGAA  2100

Query  2127   GACAAGAGATATGGCCCAGCATTAAGCATCAATGAACTGAGCAATCTTGCAAAAGGAGAG  2186
              ||||||||||||||||||||||||||||||||||||||||||||||||||||||||||||
Sbjct  2101   GACAAGAGATATGGCCCAGCATTAAGCATCAATGAACTGAGCAATCTTGCAAAAGGAGAG  2160

Query  2187   AAGGCTAATGTGCTAATTGGGCAAGGGGACGTAGTGTTGGTAATGAAACGAAAACGGGAC  2246
              ||||||||||||||||||||||||||||||||||||||||||||||||||||||||||||
```

```
Sbjct  2161  AAGGCTAATGTGCTAATTGGGCAAGGGGACGTAGTGTTGGTAATGAAACGAAAACGGGAC  2220

Query  2247  TCTAGCATACTTACTGACAGCCAGACAGCGACCAAAAGAATTCGGATGGCCATCAATTAG  2306
             ||||||||||||||||||||||||||||||||||||||||||||||||||||||||||||
Sbjct  2221  TCTAGCATACTTACTGACAGCCAGACAGCGACCAAAAGAATTCGGATGGCCATCAATTAG  2280
```

2. Basic polymerase 1 (PB1)

```
Identities = 2272/2273 (99%), Gaps = 0/2273 (0%)

Query  11    TGGATGTCAATCCGACTCTACTTTTCCTAAAAATTCCAGCGCAAAATGCCATAAGCACCA  70
             ||||||||||||||||||||||||||||||||||||||||||||||||||||||||||||
Sbjct  2     TGGATGTCAATCCGACTCTACTTTTCCTAAAAATTCCAGCGCAAAATGCCATAAGCACCA  61

Query  71    CATTCCCTTATACTGGAGATCCTCCATACAGCCATGGAACAGGAACAGGATACACCATGG  130
             ||||||||||||||||||||||||||||||||||||||||||||||||||||||||||||
Sbjct  62    CATTCCCTTATACTGGAGATCCTCCATACAGCCATGGAACAGGAACAGGATACACCATGG  121

Query  131   ACACAGTAAACAGAACACACCAATACTCAGAAAAGGGAAAGTGGACGACAAACACAGAGA  190
             ||||||||||||||||||||||||||||||||||||||||||||||||||||||||||||
Sbjct  122   ACACAGTAAACAGAACACACCAATACTCAGAAAAGGGAAAGTGGACGACAAACACAGAGA  181

Query  191   CTGGTGCACCCCAGCTCAACCCGATTGATGGACCACTACCTGAGGATAATGAACCAAGTG  250
             ||||||||||||||||||||||||||||||||||||||||||||||||||||||||||||
Sbjct  182   CTGGTGCACCCCAGCTCAACCCGATTGATGGACCACTACCTGAGGATAATGAACCAAGTG  241

Query  251   GGTATGCACAAACAGACTGTGTTCTAGAGGCTATGGCTTTCCTTGAAGAATCCCACCCAG  310
             ||||||||||||||||||||||||||||||||||||||||||||||||||||||||||||
Sbjct  242   GGTATGCACAAACAGACTGTGTTCTAGAGGCTATGGCTTTCCTTGAAGAATCCCACCCAG  301

Query  311   GAATATTTGAGAATTCATGCCTTGAAACAATGGAAGTTGTTCAACAAACAAGGGTAGATA  370
             ||||||||||||||||||||||||||||||||||||||||||||||||||||||||||||
Sbjct  302   GAATATTTGAGAATTCATGCCTTGAAACAATGGAAGTTGTTCAACAAACAAGGGTAGATA  361

Query  371   AACTAACTCAAGGTCGCCAGACTTATGATTGGACATTAAACAGAAATCAACCGGCAGCAA  430
             ||||||||||||||||||||||||||||||||||||||||||||||||||||||||||||
Sbjct  362   AACTAACTCAAGGTCGCCAGACTTATGATTGGACATTAAACAGAAATCAACCGGCAGCAA  421

Query  431   CTGCATTGGCCAACACCATAGAAGTCTTTAGATCGAATGGCCTAACAGCTAATGAGTCAG  490
             ||||||||||||||||||||||||||||||||||||||||||||||||||||||||||||
Sbjct  422   CTGCATTGGCCAACACCATAGAAGTCTTTAGATCGAATGGCCTAACAGCTAATGAGTCAG  481

Query  491   GAAGGCTAATAGATTTCTTAAAGGATGTAATGGAATCAATGAACAAAGAGGAAATAGAGA  550
             ||||||||||||||||||||||||||||||||||||||||||||||||||||||||||||
Sbjct  482   GAAGGCTAATAGATTTCTTAAAGGATGTAATGGAATCAATGAACAAAGAGGAAATAGAGA  541

Query  551   TAACAACCCACTTTCAAAGAAAAAGGAGAGTAAGAGACAACATGACCAAGAAGATGGTCA  610
             ||||||||||||||||||||||||||||||||||||||||||||||||||||||||||||
Sbjct  542   TAACAACCCACTTTCAAAGAAAAAGGAGAGTAAGAGACAACATGACCAAGAAGATGGTCA  601

Query  611   CGCAAAGAACAATAGGGAAGAAAAAACAAAGACTGAATAAGAGAGGCTATCTAATAAGAG  670
             ||||||||||||||||||||||||||||||||||||||||||||||||||||||||||||
Sbjct  602   CGCAAAGAACAATAGGGAAGAAAAAACAAAGACTGAATAAGAGAGGCTATCTAATAAGAG  661

Query  671   CACTGACATTAAATACGATGACCAAAGATGCAGAGAGAGGCAAGTTAAAAAGAAGGGCTA  730
             ||||||||||||||||||||||||||||||||||||||||||||||||||||||||||||
Sbjct  662   CACTGACATTAAATACGATGACCAAAGATGCAGAGAGAGGCAAGTTAAAAAGAAGGGCTA  721

Query  731   TCGCAACACCTGGGATGCAGATTAGAGGTTTCGTATACTTTGTTGAAACTTTAGCTAGGA  790
             ||||||||||||||||||||||||||||||||||||||||||||||||||||||||||||
Sbjct  722   TCGCAACACCTGGGATGCAGATTAGAGGTTTCGTATACTTTGTTGAAACTTTAGCTAGGA  781

Query  791   GCATTTGCGAAAAGCTTGAACAGTCTGGGCTCCCAGTAGGGGGCAATGAAAAGAAGGCCA  850
             ||||||||||||||||||||||||||||||||||||||||||||||||||||||||||||
Sbjct  782   GCATTTGCGAAAAGCTTGAACAGTCTGGGCTCCCAGTAGGGGGCAATGAAAAGAAGGCCA  841

Query  851   AACTGGCAAATGTTGTGAGAAAGATGATGACTAATTCACAAGACACAGAGATTTCTTTCA  910
             ||||||||||||||||||||||||||||||||||||||||||||||||||||||||||||
Sbjct  842   AACTGGCAAATGTTGTGAGAAAGATGATGACTAATTCACAAGACACAGAGATTTCTTTCA  901

Query  911   CAATCACTGGGGACAACACTAAGTGGAATGAAAATCAAAATCCTCGAATGTTCCTGGCGA  970
             ||||||||||||||||||||||||||||||||||||||||||||||||||||||||||||
```

```
Sbjct  902   CAATCACTGGGGACAACACTAAGTGGAATGAAAATCAAAATCCTCGAATGTTCCTGGCGA  961

Query  971   TGATTACATATATCACCAGAAATCAACCCGAGTGGTTCAGAAACATCCTGAGCATGGCAC  1030
             ||||||||||||||||||||||||||||||||||||||||||||||||||||||||||||
Sbjct  962   TGATTACATATATCACCAGAAATCAACCCGAGTGGTTCAGAAACATCCTGAGCATGGCAC  1021

Query  1031  CCATAATGTTCTCAAACAAAATGGCAAGACTAGGGAAAGGGTACATGTTCGAGAGTAAAA  1090
             ||||||||||||||||||||||||||||||||||||||||||||||||||||||||||||
Sbjct  1022  CCATAATGTTCTCAAACAAAATGGCAAGACTAGGGAAAGGGTACATGTTCGAGAGTAAAA  1081

Query  1091  GAATGAAGATTCGAACACAAATACCAGCAGAAATGCTAGCAAGCATTGACCTGAAGTACT  1150
             ||||||||||||||||||||||||||||||||||||||||||||||||||||||||||||
Sbjct  1082  GAATGAAGATTCGAACACAAATACCAGCAGAAATGCTAGCAAGCATTGACCTGAAGTACT  1141

Query  1151  TCAATGAATCAACAAAGAAGAAAATTGAGAAAATAAGGCCTCTTCTAATAGATGGCACAG  1210
             ||||||||||||||||||||||||||||||||||||||||||||||||||||||||||||
Sbjct  1142  TCAATGAATCAACAAAGAAGAAAATTGAGAAAATAAGGCCTCTTCTAATAGATGGCACAG  1201

Query  1211  CATCACTGAGTCCTGGGATGATGATGGGCATGTTCAACATGCTAAGTACGGTCTTGGGAG  1270
             ||||||||||||||||||||||||||||||||||||||||||||||||||||||||||||
Sbjct  1202  CATCACTGAGTCCTGGGATGATGATGGGCATGTTCAACATGCTAAGTACGGTCTTGGGAG  1261

Query  1271  TCTCGATACTGAATCTTGGACAAAAGAAATACACCAAGACAATATACTGGTGGGATGGGC  1330
             ||||||||||||||||||||||||||||||||||||||||||||||||||||||||||||
Sbjct  1262  TCTCGATACTGAATCTTGGACAAAAGAAATACACCAAGACAATATACTGGTGGGATGGGC  1321

Query  1331  TCCAATCATCCGACGATTTTGCTCTCATAGTGAATGCACCAAACCATGAGGGAATACAAG  1390
             ||||||||||||||||||||||||||||||||||||||||||||||||||||||||||||
Sbjct  1322  TCCAATCATCCGACGATTTTGCTCTCATAGTGAATGCACCAAACCATGAGGGAATACAAG  1381

Query  1391  CAGGAGTGGACAGATTCTACAGGACCTGCAAGTTAGTGGGAATCAACATGAGCAAAAAGA  1450
             ||||||||||||||||||||||||||||||||||||||||||||||||||||||||||||
Sbjct  1382  CAGGAGTGGACAGATTCTACAGGACCTGCAAGTTAGTGGGAATCAACATGAGCAAAAAGA  1441

Query  1451  AGTCCTATATAAATAAGACAGGGACATTTGAATTCACAAGCtttttttATCGCTATGGAT  1510
             ||||||||||||||||||||||||||||||||||||||||||||||||||||||||||||
Sbjct  1442  AGTCCTATATAAATAAGACAGGGACATTTGAATTCACAAGCTTTTTTTATCGCTATGGAT  1501

Query  1511  TTGTGGCTAATTTTAGCATGGAGCTACCCAGCTTTGGAGTGTCTGGAGTAAATGAATCAG  1570
             ||||||||||||||||||||||||||||||||||||||||||||||||||||||||||||
Sbjct  1502  TTGTGGCTAATTTTAGCATGGAGCTACCCAGCTTTGGAGTGTCTGGAGTAAATGAATCAG  1561

Query  1571  CTGACATGAGTATTGGAGTAACAGTGATAAAGAACAACATGATAAACAATGACCTTGGAC  1630
             ||||||||||||||||||||||||||||||||||||||||||||||||||||||||||||
Sbjct  1562  CTGACATGAGTATTGGAGTAACAGTGATAAAGAACAACATGATAAACAATGACCTTGGAC  1621

Query  1631  CTGCAACGGCCCAGATGGCTCTTCAATTGTTCATCAAAGACTACAGATACACATATAGGT  1690
             ||||||||||||||||||||||||||||||||||||||||||||||||||||||||||||
Sbjct  1622  CTGCAACGGCCCAGATGGCTCTTCAATTGTTCATCAAAGACTACAGATACACATATAGGT  1681

Query  1691  GCCATAGGGGAGACACACAAATTCAGACGAGAAGATCATTTGAGTTAAAGAAGCTGTGGG  1750
             ||||||||||||||||||||||||||||||||||||||||||||||||||||||||||||
Sbjct  1682  GCCATAGGGGAGACACACAAATTCAGACGAGAAGATCATTTGAGTTAAAGAAGCTGTGGG  1741

Query  1751  ATCAAACCCAATCAAAAGTAGGGCTATTAGTATCAGATGGAGGACCAAACTTATACAATA  1810
             |||||||||||||||| |||||||||||||||||||||||||||||||||||||||||||
Sbjct  1742  ATCAAACCCAATCAAAGGTAGGGCTATTAGTATCAGATGGAGGACCAAACTTATACAATA  1801

Query  1811  TACGGAATCTTCACATTCCTGAAGTCTGCTTAAAATGGGAGCTAATGGATGATGATTATC  1870
             ||||||||||||||||||||||||||||||||||||||||||||||||||||||||||||
Sbjct  1802  TACGGAATCTTCACATTCCTGAAGTCTGCTTAAAATGGGAGCTAATGGATGATGATTATC  1861

Query  1871  GGGGAAGACTTTGTAATCCCCTGAATCCCTTTGTCAGTCATAAAGAGATTGATTCTGTAA  1930
             ||||||||||||||||||||||||||||||||||||||||||||||||||||||||||||
Sbjct  1862  GGGGAAGACTTTGTAATCCCCTGAATCCCTTTGTCAGTCATAAAGAGATTGATTCTGTAA  1921

Query  1931  ACAATGCTGTGGTAATGCCAGCCCATGGTCCAGCCAAAAGCATGGAATATGATGCCGTTG  1990
             ||||||||||||||||||||||||||||||||||||||||||||||||||||||||||||
Sbjct  1922  ACAATGCTGTGGTAATGCCAGCCCATGGTCCAGCCAAAAGCATGGAATATGATGCCGTTG  1981

Query  1991  CAACTACACATTCCTGGATTCCCAAGAGGAATCGTTCTATTCTCAACACAAGCCAAAGGG  2050
             ||||||||||||||||||||||||||||||||||||||||||||||||||||||||||||
```

```
Sbjct  1982  CAACTACACATTCCTGGATTCCCAAGAGGAATCGTTCTATTCTCAACACAAGCCAAAGGG  2041

Query  2051  GAATTCTTGAGGATGAACAGATGTACCAGAAGTGCTGCAATCTATTCGAGAAATTTTTCC  2110
             ||||||||||||||||||||||||||||||||||||||||||||||||||||||||||||
Sbjct  2042  GAATTCTTGAGGATGAACAGATGTACCAGAAGTGCTGCAATCTATTCGAGAAATTTTTCC  2101

Query  2111  CTAGCAGTTCATATAGGAGACCGGTTGGAATTTCTAGCATGGTGGAGGCCATGGTGTCTA  2170
             ||||||||||||||||||||||||||||||||||||||||||||||||||||||||||||
Sbjct  2102  CTAGCAGTTCATATAGGAGACCGGTTGGAATTTCTAGCATGGTGGAGGCCATGGTGTCTA  2161

Query  2171  GGGCCCGGATTGATGCCAGGGTCGACTTCGAGTCTGGACGGATCAAGAAAGAAGAGTTCT  2230
             ||||||||||||||||||||||||||||||||||||||||||||||||||||||||||||
Sbjct  2162  GGGCCCGGATTGATGCCAGGGTCGACTTCGAGTCTGGACGGATCAAGAAAGAAGAGTTCT  2221

Query  2231  CTGAGATCATGAAGATCTGTTCCACCATTGAAGAACTCAGACGGCAAAAATAA  2283
             |||||||||||||||||||||||||||||||||||||||||||||||||||||
Sbjct  2222  CTGAGATCATGAAGATCTGTTCCACCATTGAAGAACTCAGACGGCAAAAATAA  2274
```

3. Acidic polymerase (PA)

```
Identities = 2148/2150 (99%), Gaps = 0/2150 (0%)

Query  24    ATGGAAGACTTTGTGCGACAATGCTTCAATCCAATGATCGTCGAGCTTGCGGAAAAGGCA  83
             |||||||||||||||||||||||||||||||||||||||||||||||||||| |||||||
Sbjct  1     ATGGAAGACTTTGTGCGACAATGCTTCAATCCAATGATCGTCGAGCTTGCGGRAAAGGCA  60

Query  84    ATGAAAGAATATGGGGAAGATCCGAAAATCGAAACTAACAAGTTTGCTGCAATATGCACA  143
             ||||||||||||||||||||||||||||||||||||||||||||||||||||||||||||
Sbjct  61    ATGAAAGAATATGGGGAAGATCCGAAAATCGAAACTAACAAGTTTGCTGCAATATGCACA  120

Query  144   CATTTGGAAGTTTGTTTCATGTATTCGGATTTCCATTTCATCGACGAACGGGGTGAATCA  203
             ||||||||||||||||||||||||||||||||||||||||||||||||||||||||||||
Sbjct  121   CATTTGGAAGTTTGTTTCATGTATTCGGATTTCCATTTCATCGACGAACGGGGTGAATCA  180

Query  204   ATAATTGTAGAATCTGGTGACCCGAATGCACTATTGAAGCACCGATTTGAGATAATTGAA  263
             ||||||||||||||||||||||||||||||||||||||||||||||||||||||||||||
Sbjct  181   ATAATTGTAGAATCTGGTGACCCGAATGCACTATTGAAGCACCGATTTGAGATAATTGAA  240

Query  264   GGAAGAGACCGAATCATGGCCTGGACAGTGGTGAACAGTATATGTAACACAACAGGGGTA  323
             ||||||||||||||||||||||||||||||||||||||||||||||||||||||||||||
Sbjct  241   GGAAGAGACCGAATCATGGCCTGGACAGTGGTGAACAGTATATGTAACACAACAGGGGTA  300

Query  324   GAGAAGCCTAAATTTCTTCCTGATTTGTATGATTACAAAGAGAACCGGTTCATTGAAATT  383
             ||||||||||||||||||||||||||||||||||||||||||||||||||||||||||||
Sbjct  301   GAGAAGCCTAAATTTCTTCCTGATTTGTATGATTACAAAGAGAACCGGTTCATTGAAATT  360

Query  384   GGAGTAACACGGAGGGAAGTCCACATATATTACCTAGAGAAAGCCAACAAAATAAAATCT  443
             ||||||||||||||||||||||||||||||||||||||||||||||||||||||||||||
Sbjct  361   GGAGTAACACGGAGGGAAGTCCACATATATTACCTAGAGAAAGCCAACAAAATAAAATCT  420

Query  444   GAGAAGACACACATTCACATCTTTTCATTCACTGGAGAGGAGATGGCCACCAAAGCGGAC  503
             ||||||||||||||||||||||||||||||||||||||||||||||||||||||||||||
Sbjct  421   GAGAAGACACACATTCACATCTTTTCATTCACTGGAGAGGAGATGGCCACCAAAGCGGAC  480

Query  504   TACACCCTTGACGAAGAGAGCAGGGCAAGAATCAAAACTAGGCTTTTCACTATAAGACAA  563
             ||||||||||||||||||||||||||||||||||||||||||||||||||||||||||||
Sbjct  481   TACACCCTTGACGAAGAGAGCAGGGCAAGAATCAAAACTAGGCTTTTCACTATAAGACAA  540

Query  564   GAAATGGCCAGTAGGAGTCTATGGGATTCCTTTCGTCAGTCCGAAAGAGGCGAAGAGACA  623
             ||||||||||||||||||||||||||||||||||||||||||||||||||||||||||||
Sbjct  541   GAAATGGCCAGTAGGAGTCTATGGGATTCCTTTCGTCAGTCCGAAAGAGGCGAAGAGACA  600

Query  624   ATTGAAGAAAAAATTTGAGATTACAGGAACTATGCGCAAGCTTGCCGACCAAAGTCTCCCA  683
             ||||||||||||||||||||||||||||||||||||||||||||||||||||||||||||
Sbjct  601   ATTGAAGAAAAAATTTGAGATTACAGGAACTATGCGCAAGCTTGCCGACCAAAGTCTCCCA  660

Query  684   CCGAACTTCCCCAGCCTTGAAAACTTTAGAGCCTATGTAGATGGATTCGAGCCGAACGGC  743
             ||||||||||||||||||||||||||||||||||||||||||||||||||||||||||||
Sbjct  661   CCGAACTTCCCCAGCCTTGAAAACTTTAGAGCCTATGTAGATGGATTCGAGCCGAACGGC  720

Query  744   TGCATTGAGGGCAAGCTTTCCCAAATGTCAAAAGAAGTGAACGCCAAAATTGAACCATTC  803
```

```
                ||||||||||||||||||||||||||||||||||||||||||||||||||||||||||||
Sbjct  721      TGCATTGAGGGCAAGCTTTCCCAAATGTCAAAAGAAGTGAACGCCAAAATTGAACCATTC  780

Query  804      TTGAGGACGACACCACGCCCCCTCAGATTGCCTGATGGGCCTCTTTGCCATCAGCGGTCA  863
                ||||||||||||||||||||||||||||||||||||||||||||||||||||||||||||
Sbjct  781      TTGAGGACGACACCACGCCCCCTCAGATTGCCTGATGGGCCTCTTTGCCATCAGCGGTCA  840

Query  864      AAGTTCCTGCTGATGGATGCTCTGAAATTAAGTATTGAAGACCCGAGTCACGAGGGGGAG  923
                ||||||||||||||||||||||||||||||||||||||||||||||||||||||||||||
Sbjct  841      AAGTTCCTGCTGATGGATGCTCTGAAATTAAGTATTGAAGACCCGAGTCACGAGGGGGAG  900

Query  924      GGAATACCACTATATGATGCAATCAAATGCATGAAGACATTCTTTGGCTGGAAAGAGCCT  983
                ||||||||||||||||||||||||||||||||||||||||||||||||||||||||||||
Sbjct  901      GGAATACCACTATATGATGCAATCAAATGCATGAAGACATTCTTTGGCTGGAAAGAGCCT  960

Query  984      AACATAGTCAAACCACATGAGAAAGGCATAAATCCCAATTACCTCATGGCTTGGAAGCAG  1043
                ||||||||||||||||||||||||||||||||||||||||||||||||||||||||||||
Sbjct  961      AACATAGTCAAACCACATGAGAAAGGCATAAATCCCAATTACCTCATGGCTTGGAAGCAG  1020

Query  1044     GTGCTAGCAGAGCTACAGGACATTGAAAATGAAGAGAAGATCCCAAGGACAAAGAACATG  1103
                ||||||||||||||||||||||||||||||||||||||||||||||||||||||||||||
Sbjct  1021     GTGCTAGCAGAGCTACAGGACATTGAAAATGAAGAGAAGATCCCAAGGACAAAGAACATG  1080

Query  1104     AAGAGAACAAGCCAATTGAAGTGGGCACTCGGTGAAAATATGGCACCAGAAAAAGTAGAC  1163
                ||||||||||||||||||||||||||||||||||||||||||||||||||||||||||||
Sbjct  1081     AAGAGAACAAGCCAATTGAAGTGGGCACTCGGTGAAAATATGGCACCAGAAAAAGTAGAC  1140

Query  1164     TTTGATGACTGCAAAGATGTTGGAGACCTTAAACAGTATGACAGTGATGAGCCAGAGCCC  1223
                ||||||||||||||||||||||||||||||||||||||||||||||||||||||||||||
Sbjct  1141     TTTGATGACTGCAAAGATGTTGGAGACCTTAAACAGTATGACAGTGATGAGCCAGAGCCC  1200

Query  1224     AGATCTCTAGCAAGCTGGGTCCAAAATGAATTCAATAAGGCATGTGAATTGACTGATTCA  1283
                ||||||||||||||||||||||||||||||||||||||||||||||||||||||||||||
Sbjct  1201     AGATCTCTAGCAAGCTGGGTCCAAAATGAATTCAATAAGGCATGTGAATTGACTGATTCA  1260

Query  1284     AGCTGGATAGAACTTGATGAAATAGGAGAAGATGTTGCCCCGATTGAACATATCGCAAGC  1343
                ||||||||||||||||||||||||||||||||||||||||||||||||||||||||||||
Sbjct  1261     AGCTGGATAGAACTTGATGAAATAGGAGAAGATGTTGCCCCGATTGAACATATCGCAAGC  1320

Query  1344     ATGAGGAGGAACTATTTTACAGCAGAAGTGTCCCACTGCAGGGCTACTGAATACATAATG  1403
                ||||||||||||||||||||||||||||||||||||||||||||||||||||||||||||
Sbjct  1321     ATGAGGAGGAACTATTTTACAGCAGAAGTGTCCCACTGCAGGGCTACTGAATACATAATG  1380

Query  1404     AAGGGAGTGTACATAAATACGGCCTTGCTCAATGCATCCTGTGCAGCCATGGATGACTTT  1463
                ||||||||||||||||||||||||||||||||||||||||||||||||||||||||||||
Sbjct  1381     AAGGGAGTGTACATAAATACGGCCTTGCTCAATGCATCCTGTGCAGCCATGGATGACTTT  1440

Query  1464     CAGCTGATCCCAATGATAAGCAAATGTAGGACCAAAGAAGGAAGACGGAAAACAAACCTG  1523
                ||||||||||||||||||||||||||||||||||||||||||||||||||||||||||||
Sbjct  1441     CAGCTGATCCCAATGATAAGCAAATGTAGGACCAAAGAAGGAAGACGGAAAACAAACCTG  1500

Query  1524     TATGGGTTCATTATAAAAGGAAGGTCTCATTTGAGAAATGATACTGATGTGGTGAACTTT  1583
                ||||||||||||||||||||||||||||||||||||||||||||||||||||||||||||
Sbjct  1501     TATGGGTTCATTATAAAAGGAAGGTCTCATTTGAGAAATGATACTGATGTGGTGAACTTT  1560

Query  1584     GTAAGTATGGAGTTCTCACTCACTGACCCGAGACTGGAGCCACACAAATGGGAAAAATAC  1643
                ||||||||||||||||||||||||||||||||||||||||||||||||||||||||||||
Sbjct  1561     GTAAGTATGGAGTTCTCACTCACTGACCCGAGACTGGAGCCACACAAATGGGAAAAATAC  1620

Query  1644     TGTGTTCTTGAAATAGGAGACATGCTCTTGAGGACTGCGATAGGCCAAGTGTCGAGGCCC  1703
                ||||||||||||||||||||||||||||||||||||||||||||||||||||||||||||
Sbjct  1621     TGTGTTCTTGAAATAGGAGACATGCTCTTGAGGACTGCGATAGGCCAAGTGTCGAGGCCC  1680

Query  1704     ATGTTCCTATATGTGAGAACCAATGGAACCTCCAAGATCAAGATGAAATGGGGCATGGAA  1763
                ||||||||||||||||||||||||||||||||||||||||||||||||||||||||||||
Sbjct  1681     ATGTTCCTATATGTGAGAACCAATGGAACCTCCAAGATCAAGATGAAATGGGGCATGGAA  1740

Query  1764     ATGAGGCGCTGCCTTCTTCAGTCTCTTCAGCAGATTGAGAGCATGATTGAGGCCGAGTCT  1823
                ||||||||||||||||||||||||||||||||||||||||||||||||||||||||||||
Sbjct  1741     ATGAGGCGCTGCCTTCTTCAGTCTCTTCAGCAGATTGAGAGCATGATTGAGGCCGAGTCT  1800

Query  1824     TCTGTCAAAGAGAAAGACATGACCAAGGAATTCTTTGAAAACAAATCGGAAACATGGCCA  1883
```

```
               ||||||||||||||||||||||||||||||||||||||||||||||||||||||||||||
Sbjct  1801    TCTGTCAAAGAGAAAGACATGACCAAGGAATTCTTTGAAAACAAATCGGAAACATGGCCA  1860

Query  1884    ATCGGAGAGTCACCCAGGGGAGTGGAGGAAGGCTCTATTGGGAAAGTGTGCAGGACCTTA  1943
               ||||||||||||||||||||||||||||||||||||||||||||||||||||||||||||
Sbjct  1861    ATCGGAGAGTCACCCAGGGGAGTGGAGGAAGGCTCTATTGGGAAAGTGTGCAGGACCTTA  1920

Query  1944    CTGGCAAAATCTGTATTCAACAGTCTATATGCATCTCCACAACTTGAGGGGTTTTCGGCT  2003
               |||||||||||||||||||||||||||||||| |||||||||||||||||||||||||||
Sbjct  1921    CTGGCAAAATCTGTATTCAACAGTCTATATGCGTCTCCACAACTTGAGGGGTTTTCGGCT  1980

Query  2004    GAATCTAGAAAATTGCTTCTCATTGTTCAGGCACTTAGGGACAACCTGGAACCTGGAACC  2063
               ||||||||||||||||||||||||||||||||||||||||||||||||||||||||||||
Sbjct  1981    GAATCTAGAAAATTGCTTCTCATTGTTCAGGCACTTAGGGACAACCTGGAACCTGGAACC  2040

Query  2064    TTCGATCTTGGGGGGCTATATGAAGCAATCGAGGAGTGCCTGATTAATGATCCCTGGGTT  2123
               ||||||||||||||||||||||||||||||||||||||||||||||||||||||||||||
Sbjct  2041    TTCGATCTTGGGGGGCTATATGAAGCAATCGAGGAGTGCCTGATTAATGATCCCTGGGTT  2100

Query  2124    TTGCTTAATGCATCTTGGTTCAACTCCTTCCTCACACATGCACTGAAGTA  2173
               ||||||||||||||||||||||||||||||||||||||||||||||||||
Sbjct  2101    TTGCTTAATGCATCTTGGTTCAACTCCTTCCTCACACATGCACTGAAGTA  2150
```

4. Hemagglutinin (HA)

```
Identities = 1685/1688 (99%), Gaps = 0/1688 (0%)

Query  10      TACTAGTAGTTCTGCTATATACATTTGCAACCGCAAATGCAGACACATTATGTATAGGTT  69
               ||||||||||||||||||||||||||||||||||||||||||||||||||||||||||||
Sbjct  11      TACTAGTAGTTCTGCTATATACATTTGCAACCGCAAATGCAGACACATTATGTATAGGTT  70

Query  70      ATCATGCGAACAATTCAACAGACACTGTAGACACAGTACTAGAAAAGAATGTAACAGTAA  129
               ||||||||||||||||||||||||||||||||||||||||||||||||||||||||||||
Sbjct  71      ATCATGCGAACAATTCAACAGACACTGTAGACACAGTACTAGAAAAGAATGTAACAGTAA  130

Query  130     CACACTCTGTTAACCTTCTAGAAGACAAGCATAACGGGAAACTATGCAAACTAAGAGGGG  189
               ||||||||||||||||||||||||||||||||||||||||||||||||||||||||||||
Sbjct  131     CACACTCTGTTAACCTTCTAGAAGACAAGCATAACGGGAAACTATGCAAACTAAGAGGGG  190

Query  190     TAGCCCCATTGCATTTGGGTAAATGTAACATTGCTGGCTGGATCCTGGGAAATCCAGAGT  249
               ||||||||||||||||||||||||||||||||||||||||||||||||||||||||||||
Sbjct  191     TAGCCCCATTGCATTTGGGTAAATGTAACATTGCTGGCTGGATCCTGGGAAATCCAGAGT  250

Query  250     GTGAATCACTCTCCACAGCAAGCTCATGGTCCTACATTGTGGAAACACCTAGTTCAGACA  309
               ||||||||||||||||||||||||||||||||||||||||||||||||||||||||||||
Sbjct  251     GTGAATCACTCTCCACAGCAAGCTCATGGTCCTACATTGTGGAAACACCTAGTTCAGACA  310

Query  310     ATGGAACGTGTTACCCAGGAGATTTCATCGATTATGAGGAGCTAAGAGAGCAATTGAGCT  369
               ||||||||||||||||||||||||||||||||||||||||||||||||||||||||||||
Sbjct  311     ATGGAACGTGTTACCCAGGAGATTTCATCGATTATGAGGAGCTAAGAGAGCAATTGAGCT  370

Query  370     CAGTGTCATCATTTGAAAGGTTTGAGATATTCCCCAAGACAAGTTCATGGCCCAATCATG  429
               ||||||||||||||||||||||||||||||||||||||||||||||||||||||||||||
Sbjct  371     CAGTGTCATCATTTGAAAGGTTTGAGATATTCCCCAAGACAAGTTCATGGCCCAATCATG  430

Query  430     ACTCGAACAAAGGTGTAACGGCAGCATGTCCTCATGCTGGAGCAAAAAGCTTCTACAAAA  489
               ||||||||||||||||||||||||||||||||||||||||||||||||||||||||||||
Sbjct  431     ACTCGAACAAAGGTGTAACGGCAGCATGTCCTCATGCTGGAGCAAAAAGCTTCTACAAAA  490

Query  490     ATTTAATATGGCTAGTTAAAAAAGAAAATTCATACCCAAAGCTCAGCAAATCCTACATTA  549
               |||||||||||||||||||||||| |||||||||||||||||||||||||||||||||||
Sbjct  491     ATTTAATATGGCTAGTTAAAAAAGGAAATTCATACCCAAAGCTCAGCAAATCCTACATTA  550

Query  550     ATGATAAAGGGAAAGAAGTCCTCGTGCTATGGGGCATTCACCATCCATCTACTAGTGCTG  609
               ||||||||||||||||||||||||||||||||||||||||||||||||||||||||||||
Sbjct  551     ATGATAAAGGGAAAGAAGTCCTCGTGCTATGGGGCATTCACCATCCATCTACTAGTGCTG  610

Query  610     ACCAACAAAGTCTCTATCAGAATGCAGATGCATATGTTTTTGTGGGGTCATCAAGATACA  669
               ||||||||||||||||||||||||||||||||||||||||||||||||||||||||||||
Sbjct  611     ACCAACAAAGTCTCTATCAGAATGCAGATGCATATGTTTTTGTGGGGTCATCAAGATACA  670
```

```
Query  670   GCAAGAAGTTCAAGCCGGAAATAGCAATAAGACCCAAAGTGAGGGATCAAGAAGGGAGAA  729
             |||||||||||||||||||||||||||||||||||||||||||||| || |||||||||||
Sbjct  671   GCAAGAAGTTCAAGCCGGAAATAGCAATAAGACCCAAAGTGAGGGRTCRAGAAGGGAGAA  730

Query  730   TGAACTATTACTGGACACTAGTAGAGCCGGGAGACAAAATAACATTCGAAGCAACTGGAA  789
             ||||||||||||||||||||||||||||||||||||||||||||||||||||||||||||
Sbjct  731   TGAACTATTACTGGACACTAGTAGAGCCGGGAGACAAAATAACATTCGAAGCAACTGGAA  790

Query  790   ATCTAGTGGTACCGAGATATGCATTCGCAATGGAAAGAAATGCTGGATCTGGTATTATCA  849
             ||||||||||||||||||||||||||||||||||||||||||||||||||||||||||||
Sbjct  791   ATCTAGTGGTACCGAGATATGCATTCGCAATGGAAAGAAATGCTGGATCTGGTATTATCA  850

Query  850   TTTCAGATACACCAGTCCACGATTGCAATACAACTTGTCAAACACCCAAGGGTGCTATAA  909
             ||||||||||||||||||||||||||||||||||||||||||||||||||||||||||||
Sbjct  851   TTTCAGATACACCAGTCCACGATTGCAATACAACTTGTCAAACACCCAAGGGTGCTATAA  910

Query  910   ACACCAGCCTCCCATTTCAGAATATACATCCGATCACAATTGGAAAATGTCCAAAATATG  969
             ||||||||||||||||||||||||||||||||||||||||||||||||||||||||||||
Sbjct  911   ACACCAGCCTCCCATTTCAGAATATACATCCGATCACAATTGGAAAATGTCCAAAATATG  970

Query  970   TAAAAAGCACAAAATTGAGACTGGCCACAGGATTGAGGAATATCCCGTCTATTCAATCTA  1029
             ||||||||||||||||||||||||||||||||||||||||||||||||||||||||||||
Sbjct  971   TAAAAAGCACAAAATTGAGACTGGCCACAGGATTGAGGAATATCCCGTCTATTCAATCTA  1030

Query  1030  GAGGCCTATTTGGGGCCATTGCCGGTTTCATTGAAGGGGGGTGGACAGGGATGGTAGATG  1089
             ||||||||||||||||||||||||||||||||||||||||||||||||||||||||||||
Sbjct  1031  GAGGCCTATTTGGGGCCATTGCCGGTTTCATTGAAGGGGGGTGGACAGGGATGGTAGATG  1090

Query  1090  GATGGTACGGTTATCACCATCAAAATGAGCAGGGGTCAGGATATGCAGCCGACCTGAAGA  1149
             ||||||||||||||||||||||||||||||||||||||||||||||||||||||||||||
Sbjct  1091  GATGGTACGGTTATCACCATCAAAATGAGCAGGGGTCAGGATATGCAGCCGACCTGAAGA  1150

Query  1150  GCACACAGAATGCCATTGACGAGATTACTAACAAAGTAAATTCTGTTATTGAAAAGATGA  1209
             ||||||||||||||||||||||||||||||||||||||||||||||||||||||||||||
Sbjct  1151  GCACACAGAATGCCATTGACGAGATTACTAACAAAGTAAATTCTGTTATTGAAAAGATGA  1210

Query  1210  ATACACAGTTCACAGCAGTAGGTAAAGAGTTCAACCACCTGGAAAAAAGAATAGAGAATT  1269
             ||||||||||||||||||||||||||||||||||||||||||||||||||||||||||||
Sbjct  1211  ATACACAGTTCACAGCAGTAGGTAAAGAGTTCAACCACCTGGAAAAAAGAATAGAGAATT  1270

Query  1270  TAAATAAAAAAGTTGATGATGGTTTCCTGGACATTTGGACTTACAATGCCGAACTGTTGG  1329
             ||||||||||||||||||||||||||||||||||||||||||||||||||||||||||||
Sbjct  1271  TAAATAAAAAAGTTGATGATGGTTTCCTGGACATTTGGACTTACAATGCCGAACTGTTGG  1330

Query  1330  TTCTATTGGAAAATGAAAGAACTTTGGACTACCACGATTCAAATGTGAAGAACTTATATG  1389
             ||||||||||||||||||||||||||||||||||||||||||||||||||||||||||||
Sbjct  1331  TTCTATTGGAAAATGAAAGAACTTTGGACTACCACGATTCAAATGTGAAGAACTTATATG  1390

Query  1390  AAAAGGTAAGAAGCCAGCTAAAAAACAATGCCAAGGAAATTGGAAACGGCTGCTTTGAAT  1449
             ||||||||||||||||||||||||||||||||||||||||||||||||||||||||||||
Sbjct  1391  AAAAGGTAAGAAGCCAGCTAAAAAACAATGCCAAGGAAATTGGAAACGGCTGCTTTGAAT  1450

Query  1450  TTTACCACAAATGCGATAACACGTGCATGGAAAGTGTCAAAAATGGGACTTATGACTACC  1509
             ||||||||||||||||||||||||||||||||||||||||||||||||||||||||||||
Sbjct  1451  TTTACCACAAATGCGATAACACGTGCATGGAAAGTGTCAAAAATGGGACTTATGACTACC  1510

Query  1510  CAAAATACTCAGAGGAAGCAAAATTAAACAGAGAAGAAATAGATGGGGTAAAGCTGGAAT  1569
             ||||||||||||||||||||||||||||||||||||||||||||||||||||||||||||
Sbjct  1511  CAAAATACTCAGAGGAAGCAAAATTAAACAGAGAAGAAATAGATGGGGTAAAGCTGGAAT  1570

Query  1570  CAACAAGGATTTACCAGATTTTGGCGATCTATTCAACTGTCGCCAGTTCATTGGTACTGG  1629
             ||||||||||||||||||||||||||||||||||||||||||||||||||||||||||||
Sbjct  1571  CAACAAGGATTTACCAGATTTTGGCGATCTATTCAACTGTCGCCAGTTCATTGGTACTGG  1630

Query  1630  TAGTCTCCCTGGGGGCAATCAGTTTCTGGATGTGCTCTAATGGGTCTCTACAGTGTAGAA  1689
             ||||||||||||||||||||||||||||||||||||||||||||||||||||||||||||
Sbjct  1631  TAGTCTCCCTGGGGGCAATCAGTTTCTGGATGTGCTCTAATGGGTCTCTACAGTGTAGAA  1690

Query  1690  TATGTATT  1697
             ||||||||
```

```
Sbjct  1691  TATGTATT  1698
```

5. Nucleoprotein (NP)

```
Identities = 1492/1495 (99%), Gaps = 0/1495 (0%)

Query  30    ATGGCGTCCCAAGGCACCAAACGATCATATGAACAAATGGAGACTGGTGGGGAGCGCCAG  89
             |||||||| |||||||||||||||||||||||||||||||||||||||||||||||||||
Sbjct  1     ATGGCGTCTCAAGGCACCAAACGATCATATGAACAAATGGAGACTGGTGGGGAGCGCCAG  60

Query  90    GATGCCACAGAAATCAGAGCATCTGTCGGAAGAATGATTGGTGGAATCGGGAGATTCTAC  149
             ||||||||||||||||||||||||||||||||||||||||||||||||||||||||||||
Sbjct  61    GATGCCACAGAAATCAGAGCATCTGTCGGAAGAATGATTGGTGGAATCGGGAGATTCTAC  120

Query  150   ATCCAAATGTGCACTGAACTCAAACTCAGTGATTATGATGGACGACTAATCCAGAATAGC  209
             ||||||||||||||||||||||||||||||||||||||||||||||||||||||||||||
Sbjct  121   ATCCAAATGTGCACTGAACTCAAACTCAGTGATTATGATGGACGACTAATCCAGAATAGC  180

Query  210   ATAACAATAGAGAGGATGGTGCTTTCTGCTTTTGATGAGAGAAGAAATAAATACCTAGAA  269
             ||||||||||||||||||||||||||||||||||||||||||||||||||||||||||||
Sbjct  181   ATAACAATAGAGAGGATGGTGCTTTCTGCTTTTGATGAGAGAAGAAATAAATACCTAGAA  240

Query  270   GAGCATCCCAGTGCTGGGAAGGACCCTAAGAAAACAGGAGGACCCATATATAGAAGAGTA  329
             ||||||||||||||||||||||||||||||||||||||||||||||||||||||||||||
Sbjct  241   GAGCATCCCAGTGCTGGGAAGGACCCTAAGAAAACAGGAGGACCCATATATAGAAGAGTA  300

Query  330   GACGGAAAGTGGATGAGAGAACTCATCCTTTATGACAAAGAAGAAATAAGGAGAGTTTGG  389
             |||||||||||||||||||||||||||||||||||||||| |||||||||||||||||||
Sbjct  301   GACGGAAAGTGGATGAGAGAACTCATCCTTTATGACAAAGRAGAAATAAGGAGAGTTTGG  360

Query  390   CGCCAAGCAAACAATGGCGAAGATGCAACAGCAGGTCTTACTCATATCATGATTTGGCAT  449
             |||| |||||||||||||||||||||||||||||||||||||||||||||||||||||||
Sbjct  361   CGCCTAGCAAACAATGGCGAAGATGCAACAGCAGGTCTTACTCATATCATGATTTGGCAT  420

Query  450   TCCAACCTGAATGATGCCACATATCAGAGAACAAGAGCGCTTGTTCGCACCGGAATGGAT  509
             ||||||||||||||||||||||||||||||||||||||||||||||||||||||||||||
Sbjct  421   TCCAACCTGAATGATGCCACATATCAGAGAACAAGAGCGCTTGTTCGCACCGGAATGGAT  480

Query  510   CCCAGAATGTGCTCTCTAATGCAAGGTTCAACACTTCCCAGAAGGTCTGGTGCCGCAGGT  569
             ||||||||||||||||||||||||||||||||||||||||||||||||||||||||||||
Sbjct  481   CCCAGAATGTGCTCTCTAATGCAAGGTTCAACACTTCCCAGAAGGTCTGGTGCCGCAGGT  540

Query  570   GCTGCGGTGAAAGGAGTTGGAACAATAGCAATGGAGTTAATCAGAATGATCAAACGTGGA  629
             ||||||||||||||||||||||||||||||||||||||||||||||||||||||||||||
Sbjct  541   GCTGCGGTGAAAGGAGTTGGAACAATAGCAATGGAGTTAATCAGAATGATCAAACGTGGA  600

Query  630   ATCAATGACCGAAATTTCTGGAGGGGTGAAAATGGACGAAGGACAAGGGTTGCTTATGAA  689
             ||||||||||||||||||||||||||||||||||||||||||||||||||||||||||||
Sbjct  601   ATCAATGACCGAAATTTCTGGAGGGGTGAAAATGGACGAAGGACAAGGGTTGCTTATGAA  660

Query  690   AGAATGTGCAATATCCTCAAAGGAAAATTTCAAACAGCTGCCCAGAGGGCAATGATGGAT  749
             ||||||||||||||||||||||||||||||||||||||||||||||||||||||||||||
Sbjct  661   AGAATGTGCAATATCCTCAAAGGAAAATTTCAAACAGCTGCCCAGAGGGCAATGATGGAT  720

Query  750   CAAGTAAGAGAAAGTCGAAACCCAGGAAACGCTGAGATTGAAGACCTCATTTTCCTGGCA  809
             ||||||||||||||||||||||||||||||||||||||||||||||||||||||||||||
Sbjct  721   CAAGTAAGAGAAAGTCGAAACCCAGGAAACGCTGAGATTGAAGACCTCATTTTCCTGGCA  780

Query  810   CGGTCAGCACTCATTCTGAGGGGATCAGTTGCACATAAATCCTGCCTGCCTGCTTGTGTG  869
             ||||||||||||||||||||||||||||||||||||||||||||||||||||||||||||
Sbjct  781   CGGTCAGCACTCATTCTGAGGGGATCAGTTGCACATAAATCCTGCCTGCCTGCTTGTGTG  840

Query  870   TATGGGCTTGCAGTAGCAAGTGGGCATGACTTTGAAAGGGAAGGGTACTCACTGGTCGGG  929
             ||||||||||||||||||||||||||||||||||||||||||||||||||||||||||||
Sbjct  841   TATGGGCTTGCAGTAGCAAGTGGGCATGACTTTGAAAGGGAAGGGTACTCACTGGTCGGG  900

Query  930   ATAGACCCATTCAAATTACTCCAAAACAGCCAAGTGGTCAGCCTGATGAGACCAAATGAA  989
             ||||||||||||||||||||||||||||||||||||||||||||||||||||||||||||
Sbjct  901   ATAGACCCATTCAAATTACTCCAAAACAGCCAAGTGGTCAGCCTGATGAGACCAAATGAA  960

Query  990   AACCCAGCTCACAAGAGTCAATTGGTGTGGATGGCATGCCACTCTGCTGCATTTGAAGAT  1049
```

```
                ||||||||||||||||||||||||||||||||||||||||||||||||||||||||||||
Sbjct  961   AACCCAGCTCACAAGAGTCAATTGGTGTGGATGGCATGCCACTCTGCTGCATTTGAAGAT  1020

Query  1050  TTAAGAGTATCAAGTTTCATAAGAGGAAAGAAAGTGATTCCAAGAGGAAAGCTTTCCACA  1109
             ||||||||||||||||||||||||||||||||||||||||||||||||||||||||||||
Sbjct  1021  TTAAGAGTATCAAGTTTCATAAGAGGAAAGAAAGTGATTCCAAGAGGAAAGCTTTCCACA  1080

Query  1110  AGAGGGGTCCAGATTGCTTCAAATGAGAATGTGGAAACCATGGACTCCAATACCCTGGAA  1169
             ||||||||||||||||||||||||||||||||||||||||||||||||||||||||||||
Sbjct  1081  AGAGGGGTCCAGATTGCTTCAAATGAGAATGTGGAAACCATGGACTCCAATACCCTGGAA  1140

Query  1170  CTGAGAAGCAGATACTGGGCCATAAGGACCAGGAGTGGAGGAAATACCAATCAACAAAAG  1229
             ||||||||||||||||||||||||||||||||||||||||||||||||||||||||||||
Sbjct  1141  CTGAGAAGCAGATACTGGGCCATAAGGACCAGGAGTGGAGGAAATACCAATCAACAAAAG  1200

Query  1230  GCATCCGCAGGCCAGATCAGTGTGCAGCCTACATTCTCAGTGCAGCGGAATCTCCCTTTT  1289
             ||||||||||||||||||||||||||||||||||||||||||||||||||||||||||||
Sbjct  1201  GCATCCGCAGGCCAGATCAGTGTGCAGCCTACATTCTCAGTGCAGCGGAATCTCCCTTTT  1260

Query  1290  GAAAGAGCAACCGTTATGGCAGCATTCAGCGGGAACAATGAAGGACGGACATCCGACATG  1349
             ||||||||||||||||||||||||||||||||||||||||||||||||||||||||||||
Sbjct  1261  GAAAGAGCAACCGTTATGGCAGCATTCAGCGGGAACAATGAAGGACGGACATCCGACATG  1320

Query  1350  CGAACAGAAGTTATAAGAATGATGGAAAGTGCAAAGCCAGAAGATTTGTCCTTCCAGGGG  1409
             ||||||||||||||||||||||||||||||||||||||||||||||||||||||||||||
Sbjct  1321  CGAACAGAAGTTATAAGAATGATGGAAAGTGCAAAGCCAGAAGATTTGTCCTTCCAGGGG  1380

Query  1410  CGGGGAGTCTTCGAGCTCTCGGACGAAAAGGCAACGAACCCGATCGTGCCTTCCTTTGAC  1469
             ||||||||||||||||||||||||||||||||||||||||||||||||||||||||||||
Sbjct  1381  CGGGGAGTCTTCGAGCTCTCGGACGAAAAGGCAACGAACCCGATCGTGCCTTCCTTTGAC  1440

Query  1470  ATGAGTAATGAAGGGTCTTATTTCTTCGGAGACAATGCAGAGGAGTATGACAGTT  1524
             |||||||||||||||||||||||||||||||||||||||||||||||||||||||
Sbjct  1441  ATGAGTAATGAAGGGTCTTATTTCTTCGGAGACAATGCAGAGGAGTATGACAGTT  1495
```

6. Neuraminidase (NA)

```
Identities = 1400/1400 (100%), Gaps = 0/1400 (0%)

Query  10    ACCAAAAGATAATAACCATTGGTTCGGTCTGTATGACAATTGGAATGGCTAACTTAATAT  69
             ||||||||||||||||||||||||||||||||||||||||||||||||||||||||||||
Sbjct  11    ACCAAAAGATAATAACCATTGGTTCGGTCTGTATGACAATTGGAATGGCTAACTTAATAT  70

Query  70    TACAAATTGGAAACATAATCTCAATATGGATTAGCCACTCAATTCAACTTGGGAATCAAA  129
             ||||||||||||||||||||||||||||||||||||||||||||||||||||||||||||
Sbjct  71    TACAAATTGGAAACATAATCTCAATATGGATTAGCCACTCAATTCAACTTGGGAATCAAA  130

Query  130   ATCAGATTGAAACATGCAATCAAAGCGTCATTACTTATGAAAACAACACTTGGGTAAATC  189
             ||||||||||||||||||||||||||||||||||||||||||||||||||||||||||||
Sbjct  131   ATCAGATTGAAACATGCAATCAAAGCGTCATTACTTATGAAAACAACACTTGGGTAAATC  190

Query  190   AGACATATGTTAACATCAGCAACACCAACTTTGCTGCTGGACAGTCAGTGGTTTCCGTGA  249
             ||||||||||||||||||||||||||||||||||||||||||||||||||||||||||||
Sbjct  191   AGACATATGTTAACATCAGCAACACCAACTTTGCTGCTGGACAGTCAGTGGTTTCCGTGA  250

Query  250   AATTAGCGGGCAATTCCTCTCTCTGCCCTGTTAGTGGATGGGCTATATACAGTAAAGACA  309
             ||||||||||||||||||||||||||||||||||||||||||||||||||||||||||||
Sbjct  251   AATTAGCGGGCAATTCCTCTCTCTGCCCTGTTAGTGGATGGGCTATATACAGTAAAGACA  310

Query  310   ACAGTGTAAGAATCGGTTCCAAGGGGGATGTGTTTGTCATAAGGGAACCATTCATATCAT  369
             ||||||||||||||||||||||||||||||||||||||||||||||||||||||||||||
Sbjct  311   ACAGTGTAAGAATCGGTTCCAAGGGGGATGTGTTTGTCATAAGGGAACCATTCATATCAT  370

Query  370   GCTCCCCCTTGGAATGCAGAACCTTCTTCTTGACTCAAGGGGCCTTGCTAAATGACAAAC  429
             ||||||||||||||||||||||||||||||||||||||||||||||||||||||||||||
Sbjct  371   GCTCCCCCTTGGAATGCAGAACCTTCTTCTTGACTCAAGGGGCCTTGCTAAATGACAAAC  430

Query  430   ATTCCAATGGAACCATTAAAGACAGGAGCCCATATCGAACCCTAATGAGCTGTCCTATTG  489
             ||||||||||||||||||||||||||||||||||||||||||||||||||||||||||||
Sbjct  431   ATTCCAATGGAACCATTAAAGACAGGAGCCCATATCGAACCCTAATGAGCTGTCCTATTG  490
```

```
Query  490   GTGAAGTTCCCTCTCCATACAACTCAAGATTTGAGTCAGTCGCTTGGTCAGCAAGTGCTT  549
             ||||||||||||||||||||||||||||||||||||||||||||||||||||||||||||
Sbjct  491   GTGAAGTTCCCTCTCCATACAACTCAAGATTTGAGTCAGTCGCTTGGTCAGCAAGTGCTT  550

Query  550   GTCATGATGGCATCAATTGGCTAACAATTGGAATTTCTGGCCCAGACAATGGGGCAGTGG  609
             ||||||||||||||||||||||||||||||||||||||||||||||||||||||||||||
Sbjct  551   GTCATGATGGCATCAATTGGCTAACAATTGGAATTTCTGGCCCAGACAATGGGGCAGTGG  610

Query  610   CTGTGTTAAAGTACAACGGCATAATAACAGACACTATCAAGAGTTGGAGAAACAATATAT  669
             ||||||||||||||||||||||||||||||||||||||||||||||||||||||||||||
Sbjct  611   CTGTGTTAAAGTACAACGGCATAATAACAGACACTATCAAGAGTTGGAGAAACAATATAT  670

Query  670   TGAGAACACAAGAGTCTGAATGTGCATGTGTAAATGGTTCTTGCTTTACTGTAATGACCG  729
             ||||||||||||||||||||||||||||||||||||||||||||||||||||||||||||
Sbjct  671   TGAGAACACAAGAGTCTGAATGTGCATGTGTAAATGGTTCTTGCTTTACTGTAATGACCG  730

Query  730   ATGGACCAAGTAATGGACAGGCCTCATACAAGATCTTCAGAATAGAAAAGGGAAAGATAG  789
             ||||||||||||||||||||||||||||||||||||||||||||||||||||||||||||
Sbjct  731   ATGGACCAAGTAATGGACAGGCCTCATACAAGATCTTCAGAATAGAAAAGGGAAAGATAG  790

Query  790   TCAAATCAGTCGAAATGAATGCCCCTAATTATCACTATGAGGAATGCTCCTGTTATCCTG  849
             ||||||||||||||||||||||||||||||||||||||||||||||||||||||||||||
Sbjct  791   TCAAATCAGTCGAAATGAATGCCCCTAATTATCACTATGAGGAATGCTCCTGTTATCCTG  850

Query  850   ATTCTAGTGAAATCACATGTGTGTGCAGGGATAACTGGCATGGCTCGAATCGACCGTGGG  909
             ||||||||||||||||||||||||||||||||||||||||||||||||||||||||||||
Sbjct  851   ATTCTAGTGAAATCACATGTGTGTGCAGGGATAACTGGCATGGCTCGAATCGACCGTGGG  910

Query  910   TGTCTTTCAACCAGAATCTGGAATATCAGATAGGATACATATGCAGTGGGATTTTCGGAG  969
             ||||||||||||||||||||||||||||||||||||||||||||||||||||||||||||
Sbjct  911   TGTCTTTCAACCAGAATCTGGAATATCAGATAGGATACATATGCAGTGGGATTTTCGGAG  970

Query  970   ACAATCCACGCCCTAATGATAAGACAGGCAGTTGTGGTCCAGTATCGTCTAATGGAGCAA  1029
             ||||||||||||||||||||||||||||||||||||||||||||||||||||||||||||
Sbjct  971   ACAATCCACGCCCTAATGATAAGACAGGCAGTTGTGGTCCAGTATCGTCTAATGGAGCAA  1030

Query  1030  ATGGAGTAAAAGGGTTTTCATTCAAATACGGCAATGGTGTTTGGATAGGGAGAACTAAAA  1089
             ||||||||||||||||||||||||||||||||||||||||||||||||||||||||||||
Sbjct  1031  ATGGAGTAAAAGGGTTTTCATTCAAATACGGCAATGGTGTTTGGATAGGGAGAACTAAAA  1090

Query  1090  GCATTAGTTCAAGAAACGGTTTTGAGATGATTTGGGATCCGAACGGATGGACTGGGACAG  1149
             ||||||||||||||||||||||||||||||||||||||||||||||||||||||||||||
Sbjct  1091  GCATTAGTTCAAGAAACGGTTTTGAGATGATTTGGGATCCGAACGGATGGACTGGGACAG  1150

Query  1150  ACAATAACTTCTCAATAAAGCAAGATATCGTAGGAATAAATGAGTGGTCAGGATATAGCG  1209
             ||||||||||||||||||||||||||||||||||||||||||||||||||||||||||||
Sbjct  1151  ACAATAACTTCTCAATAAAGCAAGATATCGTAGGAATAAATGAGTGGTCAGGATATAGCG  1210

Query  1210  GGAGTTTTGTTCAGCATCCAGAACTAACAGGGCTGGATTGTATAAGACCTTGCTTCTGGG  1269
             ||||||||||||||||||||||||||||||||||||||||||||||||||||||||||||
Sbjct  1211  GGAGTTTTGTTCAGCATCCAGAACTAACAGGGCTGGATTGTATAAGACCTTGCTTCTGGG  1270

Query  1270  TTGAACTAATCAGAGGGCGACCCAAAGAGAACACAATCTGGACTAGCGGGAGCAGCATAT  1329
             ||||||||||||||||||||||||||||||||||||||||||||||||||||||||||||
Sbjct  1271  TTGAACTAATCAGAGGGCGACCCAAAGAGAACACAATCTGGACTAGCGGGAGCAGCATAT  1330

Query  1330  CCTTTTGTGGTGTAAACAGTGACACTGTGGGTTGGTCTTGGCCAGACGGTGCTGAGTTGC  1389
             ||||||||||||||||||||||||||||||||||||||||||||||||||||||||||||
Sbjct  1331  CCTTTTGTGGTGTAAACAGTGACACTGTGGGTTGGTCTTGGCCAGACGGTGCTGAGTTGC  1390

Query  1390  CATTTACCATTGACAAGTAA  1409
             ||||||||||||||||||||
Sbjct  1391  CATTTACCATTGACAAGTAA  1410
```

7. Matrix (M)

```
Identities = 855/855 (100%), Gaps = 0/855 (0%)

Query  26   ATGGACTCCAACACCATGTCAAGCTTTCAGGTAGACTGTTTCCTTTGGCATATCCGCAAG  85
            ||||||||||||||||||||||||||||||||||||||||||||||||||||||||||||
Sbjct  1    ATGGACTCCAACACCATGTCAAGCTTTCAGGTAGACTGTTTCCTTTGGCATATCCGCAAG  60
```

```
Query  86   CGATTTGCAGACAATGGATTGGGTGATGCCCCATTCCTTGATCGGCTCCGCCGAGATCAA  145
            ||||||||||||||||||||||||||||||||||||||||||||||||||||||||||||
Sbjct  61   CGATTTGCAGACAATGGATTGGGTGATGCCCCATTCCTTGATCGGCTCCGCCGAGATCAA  120

Query  146  AAGTCCTTAAAAGGAAGAGGCAACACCCTTGGCCTCGATATCGAAACAGCCACTCTTGTT  205
            ||||||||||||||||||||||||||||||||||||||||||||||||||||||||||||
Sbjct  121  AAGTCCTTAAAAGGAAGAGGCAACACCCTTGGCCTCGATATCGAAACAGCCACTCTTGTT  180

Query  206  GGGAAACAAATCGTGGAATGGATCTTGAAAGAGGAATCCAGCGAGACACTTAGAATGACA  265
            ||||||||||||||||||||||||||||||||||||||||||||||||||||||||||||
Sbjct  181  GGGAAACAAATCGTGGAATGGATCTTGAAAGAGGAATCCAGCGAGACACTTAGAATGACA  240

Query  266  ATTGCATCTGTACCTACTTCGCGCTACCTTTCTGACATGACCCTCGAGGAAATGTCACGA  325
            ||||||||||||||||||||||||||||||||||||||||||||||||||||||||||||
Sbjct  241  ATTGCATCTGTACCTACTTCGCGCTACCTTTCTGACATGACCCTCGAGGAAATGTCACGA  300

Query  326  GACTGGTTCATGCTCATGCCTAGGCAAAAGATAATAGGCCCTCTTTGCGTGCGATTGGAC  385
            ||||||||||||||||||||||||||||||||||||||||||||||||||||||||||||
Sbjct  301  GACTGGTTCATGCTCATGCCTAGGCAAAAGATAATAGGCCCTCTTTGCGTGCGATTGGAC  360

Query  386  CAGGCGATCATGGAAAAGAACATAGTACTGAAAGCGAACTTCAGTGTAATCTTTAACCGA  445
            ||||||||||||||||||||||||||||||||||||||||||||||||||||||||||||
Sbjct  361  CAGGCGATCATGGAAAAGAACATAGTACTGAAAGCGAACTTCAGTGTAATCTTTAACCGA  420

Query  446  TTAGAGACCTTGATACTACTAAGGGCTTTCACTGAGGAGGGAGCAATAGTTGGAGAAATT  505
            ||||||||||||||||||||||||||||||||||||||||||||||||||||||||||||
Sbjct  421  TTAGAGACCTTGATACTACTAAGGGCTTTCACTGAGGAGGGAGCAATAGTTGGAGAAATT  480

Query  506  TCACCATTACCTTCTCTTCCAGGACATACTTATGAGGATGTCAAAAATGCAGTTGGGGTC  565
            ||||||||||||||||||||||||||||||||||||||||||||||||||||||||||||
Sbjct  481  TCACCATTACCTTCTCTTCCAGGACATACTTATGAGGATGTCAAAAATGCAGTTGGGGTC  540

Query  566  CTCATCGGAGGACTTGAATGGAATGGTAACACGGTTCGAGTCTCTGAAAATATACAGAGA  625
            ||||||||||||||||||||||||||||||||||||||||||||||||||||||||||||
Sbjct  541  CTCATCGGAGGACTTGAATGGAATGGTAACACGGTTCGAGTCTCTGAAAATATACAGAGA  600

Query  626  TTCGCTTGGAGAAACTGTGATGAGAATGGGAGACCTTCACTACCTCCAGAGCAGAAATGA  685
            ||||||||||||||||||||||||||||||||||||||||||||||||||||||||||||
Sbjct  601  TTCGCTTGGAGAAACTGTGATGAGAATGGGAGACCTTCACTACCTCCAGAGCAGAAATGA  660

Query  686  AAAGTGGCGAGAGCAATTGGGACAGAAATTTGAGGAAATAAGGTGGTTAATTGAAGAAAT  745
            ||||||||||||||||||||||||||||||||||||||||||||||||||||||||||||
Sbjct  661  AAAGTGGCGAGAGCAATTGGGACAGAAATTTGAGGAAATAAGGTGGTTAATTGAAGAAAT  720

Query  746  GCGGCACAGATTGAAAGCGACAGAGAATAGTTTCGAACAAATAACATTTATGCAAGCCTT  805
            ||||||||||||||||||||||||||||||||||||||||||||||||||||||||||||
Sbjct  721  GCGGCACAGATTGAAAGCGACAGAGAATAGTTTCGAACAAATAACATTTATGCAAGCCTT  780

Query  806  ACAACTACTGCTTGAAGTAGAACAAGAGATAAGAGCTTTCTCGTTTCAGCTTATTTAATG  865
            ||||||||||||||||||||||||||||||||||||||||||||||||||||||||||||
Sbjct  781  ACAACTACTGCTTGAAGTAGAACAAGAGATAAGAGCTTTCTCGTTTCAGCTTATTTAATG  840

Query  866  ATAAAAAACACCCTT  880
            |||||||||||||||
Sbjct  841  ATAAAAAACACCCTT  855
```

8. Nonstructural protein (NS)

```
Identities = 855/855 (100%), Gaps = 0/855 (0%)

Query  26   ATGGACTCCAACACCATGTCAAGCTTTCAGGTAGACTGTTTCCTTTGGCATATCCGCAAG  85
            ||||||||||||||||||||||||||||||||||||||||||||||||||||||||||||
Sbjct  1    ATGGACTCCAACACCATGTCAAGCTTTCAGGTAGACTGTTTCCTTTGGCATATCCGCAAG  60

Query  86   CGATTTGCAGACAATGGATTGGGTGATGCCCCATTCCTTGATCGGCTCCGCCGAGATCAA  145
            ||||||||||||||||||||||||||||||||||||||||||||||||||||||||||||
Sbjct  61   CGATTTGCAGACAATGGATTGGGTGATGCCCCATTCCTTGATCGGCTCCGCCGAGATCAA  120

Query  146  AAGTCCTTAAAAGGAAGAGGCAACACCCTTGGCCTCGATATCGAAACAGCCACTCTTGTT  205
```

```
                ||||||||||||||||||||||||||||||||||||||||||||||||||||||||||||
Sbjct  121  AAGTCCTTAAAAGGAAGAGGCAACACCCTTGGCCTCGATATCGAAACAGCCACTCTTGTT  180

Query  206  GGGAAACAAATCGTGGAATGGATCTTGAAAGAGGAATCCAGCGAGACACTTAGAATGACA  265
            ||||||||||||||||||||||||||||||||||||||||||||||||||||||||||||
Sbjct  181  GGGAAACAAATCGTGGAATGGATCTTGAAAGAGGAATCCAGCGAGACACTTAGAATGACA  240

Query  266  ATTGCATCTGTACCTACTTCGCGCTACCTTTCTGACATGACCCTCGAGGAAATGTCACGA  325
            ||||||||||||||||||||||||||||||||||||||||||||||||||||||||||||
Sbjct  241  ATTGCATCTGTACCTACTTCGCGCTACCTTTCTGACATGACCCTCGAGGAAATGTCACGA  300

Query  326  GACTGGTTCATGCTCATGCCTAGGCAAAAGATAATAGGCCCTCTTTGCGTGCGATTGGAC  385
            ||||||||||||||||||||||||||||||||||||||||||||||||||||||||||||
Sbjct  301  GACTGGTTCATGCTCATGCCTAGGCAAAAGATAATAGGCCCTCTTTGCGTGCGATTGGAC  360

Query  386  CAGGCGATCATGGAAAAGAACATAGTACTGAAAGCGAACTTCAGTGTAATCTTTAACCGA  445
            ||||||||||||||||||||||||||||||||||||||||||||||||||||||||||||
Sbjct  361  CAGGCGATCATGGAAAAGAACATAGTACTGAAAGCGAACTTCAGTGTAATCTTTAACCGA  420

Query  446  TTAGAGACCTTGATACTACTAAGGGCTTTCACTGAGGAGGGAGCAATAGTTGGAGAAATT  505
            ||||||||||||||||||||||||||||||||||||||||||||||||||||||||||||
Sbjct  421  TTAGAGACCTTGATACTACTAAGGGCTTTCACTGAGGAGGGAGCAATAGTTGGAGAAATT  480

Query  506  TCACCATTACCTTCTCTTCCAGGACATACTTATGAGGATGTCAAAAATGCAGTTGGGGTC  565
            ||||||||||||||||||||||||||||||||||||||||||||||||||||||||||||
Sbjct  481  TCACCATTACCTTCTCTTCCAGGACATACTTATGAGGATGTCAAAAATGCAGTTGGGGTC  540

Query  566  CTCATCGGAGGACTTGAATGGAATGGTAACACGGTTCGAGTCTCTGAAAATATACAGAGA  625
            ||||||||||||||||||||||||||||||||||||||||||||||||||||||||||||
Sbjct  541  CTCATCGGAGGACTTGAATGGAATGGTAACACGGTTCGAGTCTCTGAAAATATACAGAGA  600

Query  626  TTCGCTTGGAGAAACTGTGATGAGAATGGGAGACCTTCACTACCTCCAGAGCAGAAATGA  685
            ||||||||||||||||||||||||||||||||||||||||||||||||||||||||||||
Sbjct  601  TTCGCTTGGAGAAACTGTGATGAGAATGGGAGACCTTCACTACCTCCAGAGCAGAAATGA  660

Query  686  AAAGTGGCGAGAGCAATTGGGACAGAAATTTGAGGAAATAAGGTGGTTAATTGAAGAAAT  745
            ||||||||||||||||||||||||||||||||||||||||||||||||||||||||||||
Sbjct  661  AAAGTGGCGAGAGCAATTGGGACAGAAATTTGAGGAAATAAGGTGGTTAATTGAAGAAAT  720

Query  746  GCGGCACAGATTGAAAGCGACAGAGAATAGTTTCGAACAAATAACATTTATGCAAGCCTT  805
            ||||||||||||||||||||||||||||||||||||||||||||||||||||||||||||
Sbjct  721  GCGGCACAGATTGAAAGCGACAGAGAATAGTTTCGAACAAATAACATTTATGCAAGCCTT  780

Query  806  ACAACTACTGCTTGAAGTAGAACAAGAGATAAGAGCTTTCTCGTTTCAGCTTATTTAATG  865
            ||||||||||||||||||||||||||||||||||||||||||||||||||||||||||||
Sbjct  781  ACAACTACTGCTTGAAGTAGAACAAGAGATAAGAGCTTTCTCGTTTCAGCTTATTTAATG  840

Query  866  ATAAAAAACACCCTT  880
            |||||||||||||||
Sbjct  841  ATAAAAAACACCCTT  855
```

The reference found for SRR27490351 and the reference for Influenza A virus (A/California/07/2009(H1N1)) GCF_001343785.1.

1. Basic polymerase 2 (PB2)

```
Identities = 2279/2280 (99%), Gaps = 0/2280 (0%)

Query  27    ATGGAGAGAATAAAAGAACTGAGAGATCTAATGTCGCAGTCCCGCACTCGCGAGATACTC  86
             ||||||||||||||||||||||||||||||||||||||||||||||||||||||||||||
Sbjct  1     ATGGAGAGAATAAAAGAACTGAGAGATCTAATGTCGCAGTCCCGCACTCGCGAGATACTC  60

Query  87    ACTAAGACCACTGTGGACCATATGGCCATAATCAAAAAGTACACATCAGGAAGGCAAGAG  146
             ||||||||||||||||||||||||||||||||||||||||||||||||||||||||||||
Sbjct  61    ACTAAGACCACTGTGGACCATATGGCCATAATCAAAAAGTACACATCAGGAAGGCAAGAG  120

Query  147   AAGAACCCCGCACTCAGAATGAAGTGGATGATGGCAATGAGATACCCAATTACAGCAGAC  206
             ||||||||||||||||||||||||||||||||||||||||||||||||||||||||||||
Sbjct  121   AAGAACCCCGCACTCAGAATGAAGTGGATGATGGCAATGAGATACCCAATTACAGCAGAC  180
```

```
Query  207   AAGAGAATAATGGACATGATTCCAGAGAGGAATGAACAAGGACAAACCCTCTGGAGCAAA  266
             ||||||||||||||||||||||||||||||||||||||||||||||||||||||||||||
Sbjct  181   AAGAGAATAATGGACATGATTCCAGAGAGGAATGAACAAGGACAAACCCTCTGGAGCAAA  240

Query  267   ACAAACGATGCTGGATCAGACCGAGTGATGGTATCACCTCTGGCCGTAACATGGTGGAAT  326
             ||||||||||||||||||||||||||||||||||||||||||||||||||||||||||||
Sbjct  241   ACAAACGATGCTGGATCAGACCGAGTGATGGTATCACCTCTGGCCGTAACATGGTGGAAT  300

Query  327   AGGAATGGCCCAACAACAAGTACAGTTCATTACCCTAAGGTATATAAAACTTATTTCGAA  386
             ||||||||||||||||||||||||||||||||||||||||||||||||||||||||||||
Sbjct  301   AGGAATGGCCCAACAACAAGTACAGTTCATTACCCTAAGGTATATAAAACTTATTTCGAA  360

Query  387   AAGGTCGAAAGGTTGAAACATGGTACCTTCGGCCCTGTCCACTTCAGAAATCAAGTTAAA  446
             ||||||||||||||||||||||||||||||||||||||||||||||||||||||||||||
Sbjct  361   AAGGTCGAAAGGTTGAAACATGGTACCTTCGGCCCTGTCCACTTCAGAAATCAAGTTAAA  420

Query  447   ATAAGGAGGAGAGTTGATACAAACCCTGGCCATGCAGATCTCAGTGCCAAGGAGGCACAG  506
             ||||||||||||||||||||||||||||||||||||||||||||||||||||||||||||
Sbjct  421   ATAAGGAGGAGAGTTGATACAAACCCTGGCCATGCAGATCTCAGTGCCAAGGAGGCACAG  480

Query  507   GATGTGATTATGGAAGTTGTTTTCCCAAATGAAGTGGGGGCAAGAATACTGACATCAGAG  566
             ||||||||||||||||||||||||||||||||||||||||||||||||||||||||||||
Sbjct  481   GATGTGATTATGGAAGTTGTTTTCCCAAATGAAGTGGGGGCAAGAATACTGACATCAGAG  540

Query  567   TCACAGCTGGCAATAACAAAAGAGAAGAAAGAAGAGCTCCAGGATTGTAAAATTGCTCCC  626
             ||||||||||||||||||||||||||||||||||||||||||||||||||||||||||||
Sbjct  541   TCACAGCTGGCAATAACAAAAGAGAAGAAAGAAGAGCTCCAGGATTGTAAAATTGCTCCC  600

Query  627   TTGATGGTGGCGTACATGCTAGAAAGAGAATTGGTCCGTAAAACAAGGTTTCTCCCAGTA  686
             ||||||||||||||||||||||||||||||||||||||||||||||||||||||||||||
Sbjct  601   TTGATGGTGGCGTACATGCTAGAAAGAGAATTGGTCCGTAAAACAAGGTTTCTCCCAGTA  660

Query  687   GCCGGCGGAACAGGCAGTGTTTATATTGAAGTGTTGCACTTAACCCAAGGGACGTGCTGG  746
             ||||||||||||||||||||||||||||||||||||||||||||||||||||||||||||
Sbjct  661   GCCGGCGGAACAGGCAGTGTTTATATTGAAGTGTTGCACTTAACCCAAGGGACGTGCTGG  720

Query  747   GAGCAGATGTACACTCCAGGAGGAGAAGTGAGAAATGATGATGTTGACCAAAGTTTGATT  806
             ||||||||||||||||||||||||||||||||||||||||||||||||||||||||||||
Sbjct  721   GAGCAGATGTACACTCCAGGAGGAGAAGTGAGAAATGATGATGTTGACCAAAGTTTGATT  780

Query  807   ATCGCTGCTAGAAACATAGTAAGAAGAGCAGCAGTGTCAGCAGACCCATTAGCATCTCTC  866
             ||||||||||||||||||||||||||||||||||||||||||||||||||||||||||||
Sbjct  781   ATCGCTGCTAGAAACATAGTAAGAAGAGCAGCAGTGTCAGCAGACCCATTAGCATCTCTC  840

Query  867   TTGGAAATGTGCCACAGCACACAGATTGGAGGAGTAAGGATGGTGGACATCCTTAGACAG  926
             ||||||||||||||||||||||||||||||||||||||||||||||||||||||||||||
Sbjct  841   TTGGAAATGTGCCACAGCACACAGATTGGAGGAGTAAGGATGGTGGACATCCTTAGACAG  900

Query  927   AATCCAACTGAGGAACAAGCCGTAGACATATGCAAGGCAGCAATAGGGTTGAGGATTAGC  986
             ||||||||||||||||||||||||||||||||||||||||||||||||||||||||||||
Sbjct  901   AATCCAACTGAGGAACAAGCCGTAGACATATGCAAGGCAGCAATAGGGTTGAGGATTAGC  960

Query  987   TCATCTTTCAGTTTTGGTGGGTTCACTTTCAAAAGGACAAGCGGATCATCAGTCAAGAAA  1046
             ||||||||||||||||||||||||||||||||||||||||||||||||||||||||||||
Sbjct  961   TCATCTTTCAGTTTTGGTGGGTTCACTTTCAAAAGGACAAGCGGATCATCAGTCAAGAAA  1020

Query  1047  GAAGAAGAAGTGCTAACGGGCAACCTCCAAACACTGAAAATAAGAGTACATGAAGGGTAT  1106
             ||||||||||||||||||||||||||||||||||||||||||||||||||||||||||||
Sbjct  1021  GAAGAAGAAGTGCTAACGGGCAACCTCCAAACACTGAAAATAAGAGTACATGAAGGGTAT  1080

Query  1107  GAAGAATTCACAATGGTTGGGAGAAGAGCAACAGCTATTCTCAGAAAGGCAACCAGGAGA  1166
             ||||||||||||||||||||||||||||||||||||||||||||||||||||||||||||
Sbjct  1081  GAAGAATTCACAATGGTTGGGAGAAGAGCAACAGCTATTCTCAGAAAGGCAACCAGGAGA  1140

Query  1167  TTGATCCAGTTGATAGTAAGCGGGAGAGATGAGCAGTCAATTGCTGAGGCAATAATTGTG  1226
             ||||||||||||||||||||||||||||| ||||||||||||||||||||||||||||||
Sbjct  1141  TTGATCCAGTTGATAGTAAGCGGGAGAGACGAGCAGTCAATTGCTGAGGCAATAATTGTG  1200

Query  1227  GCCATGGTATTCTCACAGGAGGATTGCATGATCAAGGCAGTTAGGGGCGATCTGAACTTT  1286
             ||||||||||||||||||||||||||||||||||||||||||||||||||||||||||||
Sbjct  1201  GCCATGGTATTCTCACAGGAGGATTGCATGATCAAGGCAGTTAGGGGCGATCTGAACTTT  1260
```

```
Query  1287  GTCAATAGGGCAAACCAGCGACTGAACCCCATGCACCAACTCTTGAGGCATTTCCAAAAA  1346
             ||||||||||||||||||||||||||||||||||||||||||||||||||||||||||||
Sbjct  1261  GTCAATAGGGCAAACCAGCGACTGAACCCCATGCACCAACTCTTGAGGCATTTCCAAAAA  1320

Query  1347  GATGCAAAAGTGCTTTTCCAGAACTGGGGAATTGAATCCATCGACAATGTGATGGGAATG  1406
             ||||||||||||||||||||||||||||||||||||||||||||||||||||||||||||
Sbjct  1321  GATGCAAAAGTGCTTTTCCAGAACTGGGGAATTGAATCCATCGACAATGTGATGGGAATG  1380

Query  1407  ATCGGAATACTGCCCGACATGACCCCAAGCACGGAGATGTCGCTGAGAGGGATAAGAGTC  1466
             ||||||||||||||||||||||||||||||||||||||||||||||||||||||||||||
Sbjct  1381  ATCGGAATACTGCCCGACATGACCCCAAGCACGGAGATGTCGCTGAGAGGGATAAGAGTC  1440

Query  1467  AGCAAAATGGGAGTAGATGAATACTCCAGCACGGAGAGAGTGGTAGTGAGTATTGACCGA  1526
             ||||||||||||||||||||||||||||||||||||||||||||||||||||||||||||
Sbjct  1441  AGCAAAATGGGAGTAGATGAATACTCCAGCACGGAGAGAGTGGTAGTGAGTATTGACCGA  1500

Query  1527  TTTTTAAGGGTTAGAGATCAAAGAGGGAACGTACTATTGTCTCCCGAAGAAGTCAGTGAA  1586
             ||||||||||||||||||||||||||||||||||||||||||||||||||||||||||||
Sbjct  1501  TTTTTAAGGGTTAGAGATCAAAGAGGGAACGTACTATTGTCTCCCGAAGAAGTCAGTGAA  1560

Query  1587  ACGCAAGGAACTGAGAAGTTGACAATAACTTATTCGTCATCAATGATGTGGGAGATCAAT  1646
             ||||||||||||||||||||||||||||||||||||||||||||||||||||||||||||
Sbjct  1561  ACGCAAGGAACTGAGAAGTTGACAATAACTTATTCGTCATCAATGATGTGGGAGATCAAT  1620

Query  1647  GGCCCTGAGTCAGTGCTAGTCAACACTTATCAATGGATAATCAGGAACTGGGAAATTGTG  1706
             ||||||||||||||||||||||||||||||||||||||||||||||||||||||||||||
Sbjct  1621  GGCCCTGAGTCAGTGCTAGTCAACACTTATCAATGGATAATCAGGAACTGGGAAATTGTG  1680

Query  1707  AAAATTCAATGGTCACAAGATCCCACAATGTTATACAACAAAATGGAATTTGAACCATTT  1766
             ||||||||||||||||||||||||||||||||||||||||||||||||||||||||||||
Sbjct  1681  AAAATTCAATGGTCACAAGATCCCACAATGTTATACAACAAAATGGAATTTGAACCATTT  1740

Query  1767  CAGTCTCTTGTCCCTAAGGCAACCAGAAGCCGGTACAGTGGATTCGTAAGGACACTGTTC  1826
             ||||||||||||||||||||||||||||||||||||||||||||||||||||||||||||
Sbjct  1741  CAGTCTCTTGTCCCTAAGGCAACCAGAAGCCGGTACAGTGGATTCGTAAGGACACTGTTC  1800

Query  1827  CAGCAAATGCGGGATGTGCTTGGGACATTTGACACTGTCCAAATAATAAAACTTCTCCCC  1886
             ||||||||||||||||||||||||||||||||||||||||||||||||||||||||||||
Sbjct  1801  CAGCAAATGCGGGATGTGCTTGGGACATTTGACACTGTCCAAATAATAAAACTTCTCCCC  1860

Query  1887  TTTGCTGCTGCCCCACCAGAACAGAGTAGGATGCAATTTTCCTCATTGACTGTGAATGTG  1946
             ||||||||||||||||||||||||||||||||||||||||||||||||||||||||||||
Sbjct  1861  TTTGCTGCTGCCCCACCAGAACAGAGTAGGATGCAATTTTCCTCATTGACTGTGAATGTG  1920

Query  1947  AGAGGATCAGGGTTGAGGATACTGGTAAGAGGCAATTCTCCAGTATTCAATTACAACAAG  2006
             ||||||||||||||||||||||||||||||||||||||||||||||||||||||||||||
Sbjct  1921  AGAGGATCAGGGTTGAGGATACTGGTAAGAGGCAATTCTCCAGTATTCAATTACAACAAG  1980

Query  2007  GCAACCAAACGACTTACAGTTCTTGGAAAGGATGCAGGTGCATTGACTGAAGATCCAGAT  2066
             ||||||||||||||||||||||||||||||||||||||||||||||||||||||||||||
Sbjct  1981  GCAACCAAACGACTTACAGTTCTTGGAAAGGATGCAGGTGCATTGACTGAAGATCCAGAT  2040

Query  2067  GAAGGCACATCTGGGGTGGAGTCTGCTGTCCTGAGAGGATTTCTCATTTTGGGCAAAGAA  2126
             ||||||||||||||||||||||||||||||||||||||||||||||||||||||||||||
Sbjct  2041  GAAGGCACATCTGGGGTGGAGTCTGCTGTCCTGAGAGGATTTCTCATTTTGGGCAAAGAA  2100

Query  2127  GACAAGAGATATGGCCCAGCATTAAGCATCAATGAACTGAGCAATCTTGCAAAAGGAGAG  2186
             ||||||||||||||||||||||||||||||||||||||||||||||||||||||||||||
Sbjct  2101  GACAAGAGATATGGCCCAGCATTAAGCATCAATGAACTGAGCAATCTTGCAAAAGGAGAG  2160

Query  2187  AAGGCTAATGTGCTAATTGGGCAAGGGGACGTAGTGTTGGTAATGAAACGAAAACGGGAC  2246
             ||||||||||||||||||||||||||||||||||||||||||||||||||||||||||||
Sbjct  2161  AAGGCTAATGTGCTAATTGGGCAAGGGGACGTAGTGTTGGTAATGAAACGAAAACGGGAC  2220

Query  2247  TCTAGCATACTTACTGACAGCCAGACAGCGACCAAAAGAATTCGGATGGCCATCAATTAG  2306
             ||||||||||||||||||||||||||||||||||||||||||||||||||||||||||||
Sbjct  2221  TCTAGCATACTTACTGACAGCCAGACAGCGACCAAAAGAATTCGGATGGCCATCAATTAG  2280
```

2. Basic polymerase 1 (PB1)

Identities = 2271/2272 (99%), Gaps = 0/2272 (0%)

```
Query  12    GGATGTCAATCCGACTCTACTTTTCCTAAAAATTCCAGCGCAAAATGCCATAAGCACCAC  71
             ||||||||||||||||||||||||||||||||||||||||||||||||||||||||||||
Sbjct  3     GGATGTCAATCCGACTCTACTTTTCCTAAAAATTCCAGCGCAAAATGCCATAAGCACCAC  62

Query  72    ATTCCCTTATACTGGAGATCCTCCATACAGCCATGGAACAGGAACAGGATACACCATGGA  131
             ||||||||||||||||||||||||||||||||||||||||||||||||||||||||||||
Sbjct  63    ATTCCCTTATACTGGAGATCCTCCATACAGCCATGGAACAGGAACAGGATACACCATGGA  122

Query  132   CACAGTAAACAGAACACACCAATACTCAGAAAAGGGAAAGTGGACGACAAACACAGAGAC  191
             ||||||||||||||||||||||||||||||||||||||||||||||||||||||||||||
Sbjct  123   CACAGTAAACAGAACACACCAATACTCAGAAAAGGGAAAGTGGACGACAAACACAGAGAC  182

Query  192   TGGTGCACCCCAGCTCAACCCGATTGATGGACCACTACCTGAGGATAATGAACCAAGTGG  251
             ||||||||||||||||||||||||||||||||||||||||||||||||||||||||||||
Sbjct  183   TGGTGCACCCCAGCTCAACCCGATTGATGGACCACTACCTGAGGATAATGAACCAAGTGG  242

Query  252   GTATGCACAAACAGACTGTGTTCTAGAGGCTATGGCTTTCCTTGAAGAATCCCACCCAGG  311
             ||||||||||||||||||||||||||||||||||||||||||||||||||||||||||||
Sbjct  243   GTATGCACAAACAGACTGTGTTCTAGAGGCTATGGCTTTCCTTGAAGAATCCCACCCAGG  302

Query  312   AATATTTGAGAATTCATGCCTTGAAACAATGGAAGTTGTTCAACAAACAAGGGTAGATAA  371
             ||||||||||||||||||||||||||||||||||||||||||||||||||||||||||||
Sbjct  303   AATATTTGAGAATTCATGCCTTGAAACAATGGAAGTTGTTCAACAAACAAGGGTAGATAA  362

Query  372   ACTAACTCAAGGTCGCCAGACTTATGATTGGACATTAAACAGAAATCAACCGGCAGCAAC  431
             ||||||||||||||||||||||||||||||||||||||||||||||||||||||||||||
Sbjct  363   ACTAACTCAAGGTCGCCAGACTTATGATTGGACATTAAACAGAAATCAACCGGCAGCAAC  422

Query  432   TGCATTGGCCAACACCATAGAAGTCTTTAGATCGAATGGCCTAACAGCTAATGAGTCAGG  491
             ||||||||||||||||||||||||||||||||||||||||||||||||||||||||||||
Sbjct  423   TGCATTGGCCAACACCATAGAAGTCTTTAGATCGAATGGCCTAACAGCTAATGAGTCAGG  482

Query  492   AAGGCTAATAGATTTCTTAAAGGATGTAATGGAATCAATGAACAAAGAGGAAATAGAGAT  551
             ||||||||||||||||||||||||||||||||||||||||||||||||||||||||||||
Sbjct  483   AAGGCTAATAGATTTCTTAAAGGATGTAATGGAATCAATGAACAAAGAGGAAATAGAGAT  542

Query  552   AACAACCCACTTTCAAAGAAAAAGGAGAGTAAGAGACAACATGACCAAGAAGATGGTCAC  611
             ||||||||||||||||||||||||||||||||||||||||||||||||||||||||||||
Sbjct  543   AACAACCCACTTTCAAAGAAAAAGGAGAGTAAGAGACAACATGACCAAGAAGATGGTCAC  602

Query  612   GCAAAGAACAATAGGGAAGAAAAAACAAAGACTGAATAAGAGAGGCTATCTAATAAGAGC  671
             ||||||||||||||||||||||||||||||||||||||||||||||||||||||||||||
Sbjct  603   GCAAAGAACAATAGGGAAGAAAAAACAAAGACTGAATAAGAGAGGCTATCTAATAAGAGC  662

Query  672   ACTGACATTAAATACGATGACCAAAGATGCAGAGAGAGGCAAGTTAAAAAGAAGGGCTAT  731
             ||||||||||||||||||||||||||||||||||||||||||||||||||||||||||||
Sbjct  663   ACTGACATTAAATACGATGACCAAAGATGCAGAGAGAGGCAAGTTAAAAAGAAGGGCTAT  722

Query  732   CGCAACACCTGGGATGCAGATTAGAGGTTTCGTATACTTTGTTGAAACTTTAGCTAGGAG  791
             ||||||||||||||||||||||||||||||||||||||||||||||||||||||||||||
Sbjct  723   CGCAACACCTGGGATGCAGATTAGAGGTTTCGTATACTTTGTTGAAACTTTAGCTAGGAG  782

Query  792   CATTTGCGAAAAGCTTGAACAGTCTGGGCTCCCAGTAGGGGGCAATGAAAAGAAGGCCAA  851
             ||||||||||||||||||||||||||||||||||||||||||||||||||||||||||||
Sbjct  783   CATTTGCGAAAAGCTTGAACAGTCTGGGCTCCCAGTAGGGGGCAATGAAAAGAAGGCCAA  842

Query  852   ACTGGCAAATGTTGTGAGAAAGATGATGACTAATTCACAAGACACAGAGATTTCTTTCAC  911
             ||||||||||||||||||||||||||||||||||||||||||||||||||||||||||||
Sbjct  843   ACTGGCAAATGTTGTGAGAAAGATGATGACTAATTCACAAGACACAGAGATTTCTTTCAC  902

Query  912   AATCACTGGGGACAACACTAAGTGGAATGAAAATCAAAATCCTCGAATGTTCCTGGCGAT  971
             ||||||||||||||||||||||||||||||||||||||||||||||||||||||||||||
Sbjct  903   AATCACTGGGGACAACACTAAGTGGAATGAAAATCAAAATCCTCGAATGTTCCTGGCGAT  962

Query  972   GATTACATATATCACCAGAAATCAACCCGAGTGGTTCAGAAACATCCTGAGCATGGCACC  1031
             ||||||||||||||||||||||||||||||||||||||||||||||||||||||||||||
Sbjct  963   GATTACATATATCACCAGAAATCAACCCGAGTGGTTCAGAAACATCCTGAGCATGGCACC  1022

Query  1032  CATAATGTTCTCAAACAAAATGGCAAGACTAGGGAAAGGGTACATGTTCGAGAGTAAAAG  1091
             ||||||||||||||||||||||||||||||||||||||||||||||||||||||||||||
Sbjct  1023  CATAATGTTCTCAAACAAAATGGCAAGACTAGGGAAAGGGTACATGTTCGAGAGTAAAAG  1082
```

```
Query  1092  AATGAAGATTCGAACACAAATACCAGCAGAAATGCTAGCAAGCATTGACCTGAAGTACTT  1151
             ||||||||||||||||||||||||||||||||||||||||||||||||||||||||||||
Sbjct  1083  AATGAAGATTCGAACACAAATACCAGCAGAAATGCTAGCAAGCATTGACCTGAAGTACTT  1142

Query  1152  CAATGAATCAACAAAGAAGAAAATTGAGAAAATAAGGCCTCTTCTAATAGATGGCACAGC  1211
             ||||||||||||||||||||||||||||||||||||||||||||||||||||||||||||
Sbjct  1143  CAATGAATCAACAAAGAAGAAAATTGAGAAAATAAGGCCTCTTCTAATAGATGGCACAGC  1202

Query  1212  ATCACTGAGTCCTGGGATGATGATGGGCATGTTCAACATGCTAAGTACGGTCTTGGGAGT  1271
             ||||||||||||||||||||||||||||||||||||||||||||||||||||||||||||
Sbjct  1203  ATCACTGAGTCCTGGGATGATGATGGGCATGTTCAACATGCTAAGTACGGTCTTGGGAGT  1262

Query  1272  CTCGATACTGAATCTTGGACAAAAGAAATACACCAAGACAATATACTGGTGGGATGGGCT  1331
             ||||||||||||||||||||||||||||||||||||||||||||||||||||||||||||
Sbjct  1263  CTCGATACTGAATCTTGGACAAAAGAAATACACCAAGACAATATACTGGTGGGATGGGCT  1322

Query  1332  CCAATCATCCGACGATTTTGCTCTCATAGTGAATGCACCAAACCATGAGGGAATACAAGC  1391
             ||||||||||||||||||||||||||||||||||||||||||||||||||||||||||||
Sbjct  1323  CCAATCATCCGACGATTTTGCTCTCATAGTGAATGCACCAAACCATGAGGGAATACAAGC  1382

Query  1392  AGGAGTGGACAGATTCTACAGGACCTGCAAGTTAGTGGGAATCAACATGAGCAAAAAGAA  1451
             ||||||||||||||||||||||||||||||||||||||||||||||||||||||||||||
Sbjct  1383  AGGAGTGGACAGATTCTACAGGACCTGCAAGTTAGTGGGAATCAACATGAGCAAAAAGAA  1442

Query  1452  GTCCTATATAAATAAGACAGGGACATTTGAATTCACAAGCtttttttATCGCTATGGATT  1511
             ||||||||||||||||||||||||||||||||||||||||||||||||||||||||||||
Sbjct  1443  GTCCTATATAAATAAGACAGGGACATTTGAATTCACAAGCTTTTTTTATCGCTATGGATT  1502

Query  1512  TGTGGCTAATTTTAGCATGGAGCTACCCAGCTTTGGAGTGTCTGGAGTAAATGAATCAGC  1571
             ||||||||||||||||||||||||||||||||||||||||||||||||||||||||||||
Sbjct  1503  TGTGGCTAATTTTAGCATGGAGCTACCCAGCTTTGGAGTGTCTGGAGTAAATGAATCAGC  1562

Query  1572  TGACATGAGTATTGGAGTAACAGTGATAAAGAACAACATGATAAACAATGACCTTGGACC  1631
             ||||||||||||||||||||||||||||||||||||||||||||||||||||||||||||
Sbjct  1563  TGACATGAGTATTGGAGTAACAGTGATAAAGAACAACATGATAAACAATGACCTTGGACC  1622

Query  1632  TGCAACGGCCCAGATGGCTCTTCAATTGTTCATCAAAGACTACAGATACACATATAGGTG  1691
             ||||||||||||||||||||||||||||||||||||||||||||||||||||||||||||
Sbjct  1623  TGCAACGGCCCAGATGGCTCTTCAATTGTTCATCAAAGACTACAGATACACATATAGGTG  1682

Query  1692  CCATAGGGGAGACACACAAATTCAGACGAGAAGATCATTTGAGTTAAAGAAGCTGTGGGA  1751
             ||||||||||||||||||||||||||||||||||||||||||||||||||||||||||||
Sbjct  1683  CCATAGGGGAGACACACAAATTCAGACGAGAAGATCATTTGAGTTAAAGAAGCTGTGGGA  1742

Query  1752  TCAAACCCAATCAAAAGTAGGGCTATTAGTATCAGATGGAGGACCAAACTTATACAATAT  1811
             ||||||||||||||| ||||||||||||||||||||||||||||||||||||||||||||
Sbjct  1743  TCAAACCCAATCAAAGGTAGGGCTATTAGTATCAGATGGAGGACCAAACTTATACAATAT  1802

Query  1812  ACGGAATCTTCACATTCCTGAAGTCTGCTTAAAATGGGAGCTAATGGATGATGATTATCG  1871
             ||||||||||||||||||||||||||||||||||||||||||||||||||||||||||||
Sbjct  1803  ACGGAATCTTCACATTCCTGAAGTCTGCTTAAAATGGGAGCTAATGGATGATGATTATCG  1862

Query  1872  GGGAAGACTTTGTAATCCCCTGAATCCCTTTGTCAGTCATAAAGAGATTGATTCTGTAAA  1931
             ||||||||||||||||||||||||||||||||||||||||||||||||||||||||||||
Sbjct  1863  GGGAAGACTTTGTAATCCCCTGAATCCCTTTGTCAGTCATAAAGAGATTGATTCTGTAAA  1922

Query  1932  CAATGCTGTGGTAATGCCAGCCCATGGTCCAGCCAAAAGCATGGAATATGATGCCGTTGC  1991
             ||||||||||||||||||||||||||||||||||||||||||||||||||||||||||||
Sbjct  1923  CAATGCTGTGGTAATGCCAGCCCATGGTCCAGCCAAAAGCATGGAATATGATGCCGTTGC  1982

Query  1992  AACTACACATTCCTGGATTCCCAAGAGGAATCGTTCTATTCTCAACACAAGCCAAAGGGG  2051
             ||||||||||||||||||||||||||||||||||||||||||||||||||||||||||||
Sbjct  1983  AACTACACATTCCTGGATTCCCAAGAGGAATCGTTCTATTCTCAACACAAGCCAAAGGGG  2042

Query  2052  AATTCTTGAGGATGAACAGATGTACCAGAAGTGCTGCAATCTATTCGAGAAATTTTTCCC  2111
             ||||||||||||||||||||||||||||||||||||||||||||||||||||||||||||
Sbjct  2043  AATTCTTGAGGATGAACAGATGTACCAGAAGTGCTGCAATCTATTCGAGAAATTTTTCCC  2102

Query  2112  TAGCAGTTCATATAGGAGACCGGTTGGAATTTCTAGCATGGTGGAGGCCATGGTGTCTAG  2171
             ||||||||||||||||||||||||||||||||||||||||||||||||||||||||||||
Sbjct  2103  TAGCAGTTCATATAGGAGACCGGTTGGAATTTCTAGCATGGTGGAGGCCATGGTGTCTAG  2162
```

```
Query  2172  GGCCCGGATTGATGCCAGGGTCGACTTCGAGTCTGGACGGATCAAGAAAGAAGAGTTCTC  2231
             ||||||||||||||||||||||||||||||||||||||||||||||||||||||||||||
Sbjct  2163  GGCCCGGATTGATGCCAGGGTCGACTTCGAGTCTGGACGGATCAAGAAAGAAGAGTTCTC  2222

Query  2232  TGAGATCATGAAGATCTGTTCCACCATTGAAGAACTCAGACGGCAAAAATAA  2283
             ||||||||||||||||||||||||||||||||||||||||||||||||||||
Sbjct  2223  TGAGATCATGAAGATCTGTTCCACCATTGAAGAACTCAGACGGCAAAAATAA  2274
```

3. Acidic polymerase (PA)

```
Identities = 2148/2150 (99%), Gaps = 0/2150 (0%)

Query  24    ATGGAAGACTTTGTGCGACAATGCTTCAATCCAATGATCGTCGAGCTTGCGGAAAAGGCA  83
             |||||||||||||||||||||||||||||||||||||||||||||||||||| |||||||
Sbjct  1     ATGGAAGACTTTGTGCGACAATGCTTCAATCCAATGATCGTCGAGCTTGCGGRAAAGGCA  60

Query  84    ATGAAAGAATATGGGGAAGATCCGAAAATCGAAACTAACAAGTTTGCTGCAATATGCACA  143
             ||||||||||||||||||||||||||||||||||||||||||||||||||||||||||||
Sbjct  61    ATGAAAGAATATGGGGAAGATCCGAAAATCGAAACTAACAAGTTTGCTGCAATATGCACA  120

Query  144   CATTTGGAAGTTTGTTTCATGTATTCGGATTTCCATTTCATCGACGAACGGGGTGAATCA  203
             ||||||||||||||||||||||||||||||||||||||||||||||||||||||||||||
Sbjct  121   CATTTGGAAGTTTGTTTCATGTATTCGGATTTCCATTTCATCGACGAACGGGGTGAATCA  180

Query  204   ATAATTGTAGAATCTGGTGACCCGAATGCACTATTGAAGCACCGATTTGAGATAATTGAA  263
             ||||||||||||||||||||||||||||||||||||||||||||||||||||||||||||
Sbjct  181   ATAATTGTAGAATCTGGTGACCCGAATGCACTATTGAAGCACCGATTTGAGATAATTGAA  240

Query  264   GGAAGAGACCGAATCATGGCCTGGACAGTGGTGAACAGTATATGTAACACAACAGGGGTA  323
             ||||||||||||||||||||||||||||||||||||||||||||||||||||||||||||
Sbjct  241   GGAAGAGACCGAATCATGGCCTGGACAGTGGTGAACAGTATATGTAACACAACAGGGGTA  300

Query  324   GAGAAGCCTAAATTTCTTCCTGATTTGTATGATTACAAAGAGAACCGGTTCATTGAAATT  383
             ||||||||||||||||||||||||||||||||||||||||||||||||||||||||||||
Sbjct  301   GAGAAGCCTAAATTTCTTCCTGATTTGTATGATTACAAAGAGAACCGGTTCATTGAAATT  360

Query  384   GGAGTAACACGGAGGGAAGTCCACATATATTACCTAGAGAAAGCCAACAAAATAAAATCT  443
             ||||||||||||||||||||||||||||||||||||||||||||||||||||||||||||
Sbjct  361   GGAGTAACACGGAGGGAAGTCCACATATATTACCTAGAGAAAGCCAACAAAATAAAATCT  420

Query  444   GAGAAGACACACATTCACATCTTTTCATTCACTGGAGAGGAGATGGCCACCAAAGCGGAC  503
             ||||||||||||||||||||||||||||||||||||||||||||||||||||||||||||
Sbjct  421   GAGAAGACACACATTCACATCTTTTCATTCACTGGAGAGGAGATGGCCACCAAAGCGGAC  480

Query  504   TACACCCTTGACGAAGAGAGCAGGGCAAGAATCAAAACTAGGCTTTTCACTATAAGACAA  563
             ||||||||||||||||||||||||||||||||||||||||||||||||||||||||||||
Sbjct  481   TACACCCTTGACGAAGAGAGCAGGGCAAGAATCAAAACTAGGCTTTTCACTATAAGACAA  540

Query  564   GAAATGGCCAGTAGGAGTCTATGGGATTCCTTTCGTCAGTCCGAAAGAGGCGAAGAGACA  623
             ||||||||||||||||||||||||||||||||||||||||||||||||||||||||||||
Sbjct  541   GAAATGGCCAGTAGGAGTCTATGGGATTCCTTTCGTCAGTCCGAAAGAGGCGAAGAGACA  600

Query  624   ATTGAAGAAAAATTTGAGATTACAGGAACTATGCGCAAGCTTGCCGACCAAAGTCTCCCA  683
             ||||||||||||||||||||||||||||||||||||||||||||||||||||||||||||
Sbjct  601   ATTGAAGAAAAATTTGAGATTACAGGAACTATGCGCAAGCTTGCCGACCAAAGTCTCCCA  660

Query  684   CCGAACTTCCCCAGCCTTGAAAACTTTAGAGCCTATGTAGATGGATTCGAGCCGAACGGC  743
             ||||||||||||||||||||||||||||||||||||||||||||||||||||||||||||
Sbjct  661   CCGAACTTCCCCAGCCTTGAAAACTTTAGAGCCTATGTAGATGGATTCGAGCCGAACGGC  720

Query  744   TGCATTGAGGGCAAGCTTTCCCAAATGTCAAAAGAAGTGAACGCCAAAATTGAACCATTC  803
             ||||||||||||||||||||||||||||||||||||||||||||||||||||||||||||
Sbjct  721   TGCATTGAGGGCAAGCTTTCCCAAATGTCAAAAGAAGTGAACGCCAAAATTGAACCATTC  780

Query  804   TTGAGGACGACACCACGCCCCCTCAGATTGCCTGATGGGCCTCTTTGCCATCAGCGGTCA  863
             ||||||||||||||||||||||||||||||||||||||||||||||||||||||||||||
Sbjct  781   TTGAGGACGACACCACGCCCCCTCAGATTGCCTGATGGGCCTCTTTGCCATCAGCGGTCA  840

Query  864   AAGTTCCTGCTGATGGATGCTCTGAAATTAAGTATTGAAGACCCGAGTCACGAGGGGGAG  923
             ||||||||||||||||||||||||||||||||||||||||||||||||||||||||||||
Sbjct  841   AAGTTCCTGCTGATGGATGCTCTGAAATTAAGTATTGAAGACCCGAGTCACGAGGGGGAG  900
```

```
Query  924   GGAATACCACTATATGATGCAATCAAATGCATGAAGACATTCTTTGGCTGGAAAGAGCCT  983
             ||||||||||||||||||||||||||||||||||||||||||||||||||||||||||||
Sbjct  901   GGAATACCACTATATGATGCAATCAAATGCATGAAGACATTCTTTGGCTGGAAAGAGCCT  960

Query  984   AACATAGTCAAACCACATGAGAAAGGCATAAATCCCAATTACCTCATGGCTTGGAAGCAG  1043
             ||||||||||||||||||||||||||||||||||||||||||||||||||||||||||||
Sbjct  961   AACATAGTCAAACCACATGAGAAAGGCATAAATCCCAATTACCTCATGGCTTGGAAGCAG  1020

Query  1044  GTGCTAGCAGAGCTACAGGACATTGAAAATGAAGAGAAGATCCCAAGGACAAAGAACATG  1103
             ||||||||||||||||||||||||||||||||||||||||||||||||||||||||||||
Sbjct  1021  GTGCTAGCAGAGCTACAGGACATTGAAAATGAAGAGAAGATCCCAAGGACAAAGAACATG  1080

Query  1104  AAGAGAACAAGCCAATTGAAGTGGGCACTCGGTGAAAATATGGCACCAGAAAAAGTAGAC  1163
             ||||||||||||||||||||||||||||||||||||||||||||||||||||||||||||
Sbjct  1081  AAGAGAACAAGCCAATTGAAGTGGGCACTCGGTGAAAATATGGCACCAGAAAAAGTAGAC  1140

Query  1164  TTTGATGACTGCAAAGATGTTGGAGACCTTAAACAGTATGACAGTGATGAGCCAGAGCCC  1223
             ||||||||||||||||||||||||||||||||||||||||||||||||||||||||||||
Sbjct  1141  TTTGATGACTGCAAAGATGTTGGAGACCTTAAACAGTATGACAGTGATGAGCCAGAGCCC  1200

Query  1224  AGATCTCTAGCAAGCTGGGTCCAAAATGAATTCAATAAGGCATGTGAATTGACTGATTCA  1283
             ||||||||||||||||||||||||||||||||||||||||||||||||||||||||||||
Sbjct  1201  AGATCTCTAGCAAGCTGGGTCCAAAATGAATTCAATAAGGCATGTGAATTGACTGATTCA  1260

Query  1284  AGCTGGATAGAACTTGATGAAATAGGAGAAGATGTTGCCCCGATTGAACATATCGCAAGC  1343
             ||||||||||||||||||||||||||||||||||||||||||||||||||||||||||||
Sbjct  1261  AGCTGGATAGAACTTGATGAAATAGGAGAAGATGTTGCCCCGATTGAACATATCGCAAGC  1320

Query  1344  ATGAGGAGGAACTATTTTACAGCAGAAGTGTCCCACTGCAGGGCTACTGAATACATAATG  1403
             ||||||||||||||||||||||||||||||||||||||||||||||||||||||||||||
Sbjct  1321  ATGAGGAGGAACTATTTTACAGCAGAAGTGTCCCACTGCAGGGCTACTGAATACATAATG  1380

Query  1404  AAGGGAGTGTACATAAATACGGCCTTGCTCAATGCATCCTGTGCAGCCATGGATGACTTT  1463
             ||||||||||||||||||||||||||||||||||||||||||||||||||||||||||||
Sbjct  1381  AAGGGAGTGTACATAAATACGGCCTTGCTCAATGCATCCTGTGCAGCCATGGATGACTTT  1440

Query  1464  CAGCTGATCCCAATGATAAGCAAATGTAGGACCAAAGAAGGAAGACGGAAAACAAACCTG  1523
             ||||||||||||||||||||||||||||||||||||||||||||||||||||||||||||
Sbjct  1441  CAGCTGATCCCAATGATAAGCAAATGTAGGACCAAAGAAGGAAGACGGAAAACAAACCTG  1500

Query  1524  TATGGGTTCATTATAAAAGGAAGGTCTCATTTGAGAAATGATACTGATGTGGTGAACTTT  1583
             ||||||||||||||||||||||||||||||||||||||||||||||||||||||||||||
Sbjct  1501  TATGGGTTCATTATAAAAGGAAGGTCTCATTTGAGAAATGATACTGATGTGGTGAACTTT  1560

Query  1584  GTAAGTATGGAGTTCTCACTCACTGACCCGAGACTGGAGCCACACAAATGGGAAAAATAC  1643
             ||||||||||||||||||||||||||||||||||||||||||||||||||||||||||||
Sbjct  1561  GTAAGTATGGAGTTCTCACTCACTGACCCGAGACTGGAGCCACACAAATGGGAAAAATAC  1620

Query  1644  TGTGTTCTTGAAATAGGAGACATGCTCTTGAGGACTGCGATAGGCCAAGTGTCGAGGCCC  1703
             ||||||||||||||||||||||||||||||||||||||||||||||||||||||||||||
Sbjct  1621  TGTGTTCTTGAAATAGGAGACATGCTCTTGAGGACTGCGATAGGCCAAGTGTCGAGGCCC  1680

Query  1704  ATGTTCCTATATGTGAGAACCAATGGAACCTCCAAGATCAAGATGAAATGGGGCATGGAA  1763
             ||||||||||||||||||||||||||||||||||||||||||||||||||||||||||||
Sbjct  1681  ATGTTCCTATATGTGAGAACCAATGGAACCTCCAAGATCAAGATGAAATGGGGCATGGAA  1740

Query  1764  ATGAGGCGCTGCCTTCTTCAGTCTCTTCAGCAGATTGAGAGCATGATTGAGGCCGAGTCT  1823
             ||||||||||||||||||||||||||||||||||||||||||||||||||||||||||||
Sbjct  1741  ATGAGGCGCTGCCTTCTTCAGTCTCTTCAGCAGATTGAGAGCATGATTGAGGCCGAGTCT  1800

Query  1824  TCTGTCAAAGAGAAAGACATGACCAAGGAATTCTTTGAAAACAAATCGGAAACATGGCCA  1883
             ||||||||||||||||||||||||||||||||||||||||||||||||||||||||||||
Sbjct  1801  TCTGTCAAAGAGAAAGACATGACCAAGGAATTCTTTGAAAACAAATCGGAAACATGGCCA  1860

Query  1884  ATCGGAGAGTCACCCAGGGGAGTGGAGGAAGGCTCTATTGGGAAAGTGTGCAGGACCTTA  1943
             ||||||||||||||||||||||||||||||||||||||||||||||||||||||||||||
Sbjct  1861  ATCGGAGAGTCACCCAGGGGAGTGGAGGAAGGCTCTATTGGGAAAGTGTGCAGGACCTTA  1920

Query  1944  CTGGCAAAATCTGTATTCAACAGTCTATATGCATCTCCACAACTTGAGGGGTTTTCGGCT  2003
             |||||||||||||||||||||||||||||||| |||||||||||||||||||||||||||
Sbjct  1921  CTGGCAAAATCTGTATTCAACAGTCTATATGCGTCTCCACAACTTGAGGGGTTTTCGGCT  1980
```

```
Query  2004  GAATCTAGAAAATTGCTTCTCATTGTTCAGGCACTTAGGGACAACCTGGAACCTGGAACC  2063
             ||||||||||||||||||||||||||||||||||||||||||||||||||||||||||||
Sbjct  1981  GAATCTAGAAAATTGCTTCTCATTGTTCAGGCACTTAGGGACAACCTGGAACCTGGAACC  2040

Query  2064  TTCGATCTTGGGGGGCTATATGAAGCAATCGAGGAGTGCCTGATTAATGATCCCTGGGTT  2123
             ||||||||||||||||||||||||||||||||||||||||||||||||||||||||||||
Sbjct  2041  TTCGATCTTGGGGGGCTATATGAAGCAATCGAGGAGTGCCTGATTAATGATCCCTGGGTT  2100

Query  2124  TTGCTTAATGCATCTTGGTTCAACTCCTTCCTCACACATGCACTGAAGTA  2173
             ||||||||||||||||||||||||||||||||||||||||||||||||||
Sbjct  2101  TTGCTTAATGCATCTTGGTTCAACTCCTTCCTCACACATGCACTGAAGTA  2150
```

4. Hemagglutinin (HA)

```
Identities = 1684/1687 (99%), Gaps = 0/1687 (0%)

Query  11    ACTAGTAGTTCTGCTATATACATTTGCAACCGCAAATGCAGACACATTATGTATAGGTTA  70
             ||||||||||||||||||||||||||||||||||||||||||||||||||||||||||||
Sbjct  12    ACTAGTAGTTCTGCTATATACATTTGCAACCGCAAATGCAGACACATTATGTATAGGTTA  71

Query  71    TCATGCGAACAATTCAACAGACACTGTAGACACAGTACTAGAAAAGAATGTAACAGTAAC  130
             ||||||||||||||||||||||||||||||||||||||||||||||||||||||||||||
Sbjct  72    TCATGCGAACAATTCAACAGACACTGTAGACACAGTACTAGAAAAGAATGTAACAGTAAC  131

Query  131   ACACTCTGTTAACCTTCTAGAAGACAAGCATAACGGGAAACTATGCAAACTAAGAGGGGT  190
             ||||||||||||||||||||||||||||||||||||||||||||||||||||||||||||
Sbjct  132   ACACTCTGTTAACCTTCTAGAAGACAAGCATAACGGGAAACTATGCAAACTAAGAGGGGT  191

Query  191   AGCCCCATTGCATTTGGGTAAATGTAACATTGCTGGCTGGATCCTGGGAAATCCAGAGTG  250
             ||||||||||||||||||||||||||||||||||||||||||||||||||||||||||||
Sbjct  192   AGCCCCATTGCATTTGGGTAAATGTAACATTGCTGGCTGGATCCTGGGAAATCCAGAGTG  251

Query  251   TGAATCACTCTCCACAGCAAGCTCATGGTCCTACATTGTGGAAACACCTAGTTCAGACAA  310
             ||||||||||||||||||||||||||||||||||||||||||||||||||||||||||||
Sbjct  252   TGAATCACTCTCCACAGCAAGCTCATGGTCCTACATTGTGGAAACACCTAGTTCAGACAA  311


Query  311   TGGAACGTGTTACCCAGGAGATTTCATCGATTATGAGGAGCTAAGAGAGCAATTGAGCTC  370
             ||||||||||||||||||||||||||||||||||||||||||||||||||||||||||||
Sbjct  312   TGGAACGTGTTACCCAGGAGATTTCATCGATTATGAGGAGCTAAGAGAGCAATTGAGCTC  371

Query  371   AGTGTCATCATTTGAAAGGTTTGAGATATTCCCCAAGACAAGTTCATGGCCCAATCATGA  430
             ||||||||||||||||||||||||||||||||||||||||||||||||||||||||||||
Sbjct  372   AGTGTCATCATTTGAAAGGTTTGAGATATTCCCCAAGACAAGTTCATGGCCCAATCATGA  431

Query  431   CTCGAACAAAGGTGTAACGGCAGCATGTCCTCATGCTGGAGCAAAAAGCTTCTACAAAAA  490
             ||||||||||||||||||||||||||||||||||||||||||||||||||||||||||||
Sbjct  432   CTCGAACAAAGGTGTAACGGCAGCATGTCCTCATGCTGGAGCAAAAAGCTTCTACAAAAA  491

Query  491   TTTAATATGGCTAGTTAAAGAAGGAAATTCATACCCAAAGCTCAGCAAATCCTACATTAA  550
             ||||||||||||||||||| ||||||||||||||||||||||||||||||||||||||||
Sbjct  492   TTTAATATGGCTAGTTAAAAAAGGAAATTCATACCCAAAGCTCAGCAAATCCTACATTAA  551

Query  551   TGATAAAGGGAAAGAAGTCCTCGTGCTATGGGGCATTCACCATCCATCTACTAGTGCTGA  610
             ||||||||||||||||||||||||||||||||||||||||||||||||||||||||||||
Sbjct  552   TGATAAAGGGAAAGAAGTCCTCGTGCTATGGGGCATTCACCATCCATCTACTAGTGCTGA  611

Query  611   CCAACAAAGTCTCTATCAGAATGCAGATGCATATGTTTTTGTGGGGTCATCAAGATACAG  670
             ||||||||||||||||||||||||||||||||||||||||||||||||||||||||||||
Sbjct  612   CCAACAAAGTCTCTATCAGAATGCAGATGCATATGTTTTTGTGGGGTCATCAAGATACAG  671

Query  671   CAAGAAGTTCAAGCCGGAAATAGCAATAAGACCCAAAGTGAGGGATCAAGAAGGGAGAAT  730
             |||||||||||||||||||||||||||||||||||||||||||| || ||||||||||||
Sbjct  672   CAAGAAGTTCAAGCCGGAAATAGCAATAAGACCCAAAGTGAGGGRTCRAGAAGGGAGAAT  731

Query  731   GAACTATTACTGGACACTAGTAGAGCCGGGAGACAAAATAACATTCGAAGCAACTGGAAA  790
             ||||||||||||||||||||||||||||||||||||||||||||||||||||||||||||
Sbjct  732   GAACTATTACTGGACACTAGTAGAGCCGGGAGACAAAATAACATTCGAAGCAACTGGAAA  791
```

```
Query  791   TCTAGTGGTACCGAGATATGCATTCGCAATGGAAAGAAATGCTGGATCTGGTATTATCAT  850
             ||||||||||||||||||||||||||||||||||||||||||||||||||||||||||||
Sbjct  792   TCTAGTGGTACCGAGATATGCATTCGCAATGGAAAGAAATGCTGGATCTGGTATTATCAT  851

Query  851   TTCAGATACACCAGTCCACGATTGCAATACAACTTGTCAAACACCCAAGGGTGCTATAAA  910
             ||||||||||||||||||||||||||||||||||||||||||||||||||||||||||||
Sbjct  852   TTCAGATACACCAGTCCACGATTGCAATACAACTTGTCAAACACCCAAGGGTGCTATAAA  911

Query  911   CACCAGCCTCCCATTTCAGAATATACATCCGATCACAATTGGAAAATGTCCAAAATATGT  970
             ||||||||||||||||||||||||||||||||||||||||||||||||||||||||||||
Sbjct  912   CACCAGCCTCCCATTTCAGAATATACATCCGATCACAATTGGAAAATGTCCAAAATATGT  971

Query  971   AAAAAGCACAAAATTGAGACTGGCCACAGGATTGAGGAATATCCCGTCTATTCAATCTAG  1030
             ||||||||||||||||||||||||||||||||||||||||||||||||||||||||||||
Sbjct  972   AAAAAGCACAAAATTGAGACTGGCCACAGGATTGAGGAATATCCCGTCTATTCAATCTAG  1031

Query  1031  AGGCCTATTTGGGGCCATTGCCGGTTTCATTGAAGGGGGGTGGACAGGGATGGTAGATGG  1090
             ||||||||||||||||||||||||||||||||||||||||||||||||||||||||||||
Sbjct  1032  AGGCCTATTTGGGGCCATTGCCGGTTTCATTGAAGGGGGGTGGACAGGGATGGTAGATGG  1091

Query  1091  ATGGTACGGTTATCACCATCAAAATGAGCAGGGGTCAGGATATGCAGCCGACCTGAAGAG  1150
             ||||||||||||||||||||||||||||||||||||||||||||||||||||||||||||
Sbjct  1092  ATGGTACGGTTATCACCATCAAAATGAGCAGGGGTCAGGATATGCAGCCGACCTGAAGAG  1151

Query  1151  CACACAGAATGCCATTGACGAGATTACTAACAAAGTAAATTCTGTTATTGAAAAGATGAA  1210
             ||||||||||||||||||||||||||||||||||||||||||||||||||||||||||||
Sbjct  1152  CACACAGAATGCCATTGACGAGATTACTAACAAAGTAAATTCTGTTATTGAAAAGATGAA  1211

Query  1211  TACACAGTTCACAGCAGTAGGTAAAGAGTTCAACCACCTGGAAAAAAGAATAGAGAATTT  1270
             ||||||||||||||||||||||||||||||||||||||||||||||||||||||||||||
Sbjct  1212  TACACAGTTCACAGCAGTAGGTAAAGAGTTCAACCACCTGGAAAAAAGAATAGAGAATTT  1271

Query  1271  AAATAAAAAAGTTGATGATGGTTTCCTGGACATTTGGACTTACAATGCCGAACTGTTGGT  1330
             ||||||||||||||||||||||||||||||||||||||||||||||||||||||||||||
Sbjct  1272  AAATAAAAAAGTTGATGATGGTTTCCTGGACATTTGGACTTACAATGCCGAACTGTTGGT  1331

Query  1331  TCTATTGGAAAATGAAAGAACTTTGGACTACCACGATTCAAATGTGAAGAACTTATATGA  1390
             ||||||||||||||||||||||||||||||||||||||||||||||||||||||||||||
Sbjct  1332  TCTATTGGAAAATGAAAGAACTTTGGACTACCACGATTCAAATGTGAAGAACTTATATGA  1391

Query  1391  AAAGGTAAGAAGCCAGCTAAAAAACAATGCCAAGGAAATTGGAAACGGCTGCTTTGAATT  1450
             ||||||||||||||||||||||||||||||||||||||||||||||||||||||||||||
Sbjct  1392  AAAGGTAAGAAGCCAGCTAAAAAACAATGCCAAGGAAATTGGAAACGGCTGCTTTGAATT  1451

Query  1451  TTACCACAAATGCGATAACACGTGCATGGAAAGTGTCAAAAATGGGACTTATGACTACCC  1510
             ||||||||||||||||||||||||||||||||||||||||||||||||||||||||||||
Sbjct  1452  TTACCACAAATGCGATAACACGTGCATGGAAAGTGTCAAAAATGGGACTTATGACTACCC  1511

Query  1511  AAAATACTCAGAGGAAGCAAAATTAAACAGAGAAGAAATAGATGGGGTAAAGCTGGAATC  1570
             ||||||||||||||||||||||||||||||||||||||||||||||||||||||||||||
Sbjct  1512  AAAATACTCAGAGGAAGCAAAATTAAACAGAGAAGAAATAGATGGGGTAAAGCTGGAATC  1571

Query  1571  AACAAGGATTTACCAGATTTTGGCGATCTATTCAACTGTCGCCAGTTCATTGGTACTGGT  1630
             ||||||||||||||||||||||||||||||||||||||||||||||||||||||||||||
Sbjct  1572  AACAAGGATTTACCAGATTTTGGCGATCTATTCAACTGTCGCCAGTTCATTGGTACTGGT  1631

Query  1631  AGTCTCCCTGGGGGCAATCAGTTTCTGGATGTGCTCTAATGGGTCTCTACAGTGTAGAAT  1690
             ||||||||||||||||||||||||||||||||||||||||||||||||||||||||||||
Sbjct  1632  AGTCTCCCTGGGGGCAATCAGTTTCTGGATGTGCTCTAATGGGTCTCTACAGTGTAGAAT  1691

Query  1691  ATGTATT  1697
             |||||||
Sbjct  1692  ATGTATT  1698
```

5. Nucleoprotein (NP)

```
Identities = 1492/1495 (99%), Gaps = 0/1495 (0%)

Query  30    ATGGCGTCCCAAGGCACCAAACGATCATATGAACAAATGGAGACTGGTGGGGAGCGCCAG  89
             |||||||| |||||||||||||||||||||||||||||||||||||||||||||||||||
Sbjct  1     ATGGCGTCTCAAGGCACCAAACGATCATATGAACAAATGGAGACTGGTGGGGAGCGCCAG  60
```

```
Query  90    GATGCCACAGAAATCAGAGCATCTGTCGGAAGAATGATTGGTGGAATCGGGAGATTCTAC  149
             ||||||||||||||||||||||||||||||||||||||||||||||||||||||||||||
Sbjct  61    GATGCCACAGAAATCAGAGCATCTGTCGGAAGAATGATTGGTGGAATCGGGAGATTCTAC  120

Query  150   ATCCAAATGTGCACTGAACTCAAACTCAGTGATTATGATGGACGACTAATCCAGAATAGC  209
             ||||||||||||||||||||||||||||||||||||||||||||||||||||||||||||
Sbjct  121   ATCCAAATGTGCACTGAACTCAAACTCAGTGATTATGATGGACGACTAATCCAGAATAGC  180

Query  210   ATAACAATAGAGAGGATGGTGCTTTCTGCTTTTGATGAGAGAAGAAATAAATACCTAGAA  269
             ||||||||||||||||||||||||||||||||||||||||||||||||||||||||||||
Sbjct  181   ATAACAATAGAGAGGATGGTGCTTTCTGCTTTTGATGAGAGAAGAAATAAATACCTAGAA  240

Query  270   GAGCATCCCAGTGCTGGGAAGGACCCTAAGAAAACAGGAGGACCCATATATAGAAGAGTA  329
             ||||||||||||||||||||||||||||||||||||||||||||||||||||||||||||
Sbjct  241   GAGCATCCCAGTGCTGGGAAGGACCCTAAGAAAACAGGAGGACCCATATATAGAAGAGTA  300

Query  330   GACGGAAAGTGGATGAGAGAACTCATCCTTTATGACAAAGAAGAAATAAGGAGAGTTTGG  389
             |||||||||||||||||||||||||||||||||||||||| |||||||||||||||||||
Sbjct  301   GACGGAAAGTGGATGAGAGAACTCATCCTTTATGACAAAGRAGAAATAAGGAGAGTTTGG  360

Query  390   CGCCAAGCAAACAATGGCGAAGATGCAACAGCAGGTCTTACTCATATCATGATTTGGCAT  449
             |||| |||||||||||||||||||||||||||||||||||||||||||||||||||||||
Sbjct  361   CGCCTAGCAAACAATGGCGAAGATGCAACAGCAGGTCTTACTCATATCATGATTTGGCAT  420

Query  450   TCCAACCTGAATGATGCCACATATCAGAGAACAAGAGCGCTTGTTCGCACCGGAATGGAT  509
             ||||||||||||||||||||||||||||||||||||||||||||||||||||||||||||
Sbjct  421   TCCAACCTGAATGATGCCACATATCAGAGAACAAGAGCGCTTGTTCGCACCGGAATGGAT  480

Query  510   CCCAGAATGTGCTCTCTAATGCAAGGTTCAACACTTCCCAGAAGGTCTGGTGCCGCAGGT  569
             ||||||||||||||||||||||||||||||||||||||||||||||||||||||||||||
Sbjct  481   CCCAGAATGTGCTCTCTAATGCAAGGTTCAACACTTCCCAGAAGGTCTGGTGCCGCAGGT  540

Query  570   GCTGCGGTGAAAGGAGTTGGAACAATAGCAATGGAGTTAATCAGAATGATCAAACGTGGA  629
             ||||||||||||||||||||||||||||||||||||||||||||||||||||||||||||
Sbjct  541   GCTGCGGTGAAAGGAGTTGGAACAATAGCAATGGAGTTAATCAGAATGATCAAACGTGGA  600

Query  630   ATCAATGACCGAAATTTCTGGAGGGGTGAAAATGGACGAAGGACAAGGGTTGCTTATGAA  689
             ||||||||||||||||||||||||||||||||||||||||||||||||||||||||||||
Sbjct  601   ATCAATGACCGAAATTTCTGGAGGGGTGAAAATGGACGAAGGACAAGGGTTGCTTATGAA  660

Query  690   AGAATGTGCAATATCCTCAAAGGAAAATTTCAAACAGCTGCCCAGAGGGCAATGATGGAT  749
             ||||||||||||||||||||||||||||||||||||||||||||||||||||||||||||
Sbjct  661   AGAATGTGCAATATCCTCAAAGGAAAATTTCAAACAGCTGCCCAGAGGGCAATGATGGAT  720

Query  750   CAAGTAAGAGAAAGTCGAAACCCAGGAAACGCTGAGATTGAAGACCTCATTTTCCTGGCA  809
             ||||||||||||||||||||||||||||||||||||||||||||||||||||||||||||
Sbjct  721   CAAGTAAGAGAAAGTCGAAACCCAGGAAACGCTGAGATTGAAGACCTCATTTTCCTGGCA  780

Query  810   CGGTCAGCACTCATTCTGAGGGGATCAGTTGCACATAAATCCTGCCTGCCTGCTTGTGTG  869
             ||||||||||||||||||||||||||||||||||||||||||||||||||||||||||||
Sbjct  781   CGGTCAGCACTCATTCTGAGGGGATCAGTTGCACATAAATCCTGCCTGCCTGCTTGTGTG  840

Query  870   TATGGGCTTGCAGTAGCAAGTGGGCATGACTTTGAAAGGGAAGGGTACTCACTGGTCGGG  929
             ||||||||||||||||||||||||||||||||||||||||||||||||||||||||||||
Sbjct  841   TATGGGCTTGCAGTAGCAAGTGGGCATGACTTTGAAAGGGAAGGGTACTCACTGGTCGGG  900

Query  930   ATAGACCCATTCAAATTACTCCAAAACAGCCAAGTGGTCAGCCTGATGAGACCAAATGAA  989
             ||||||||||||||||||||||||||||||||||||||||||||||||||||||||||||
Sbjct  901   ATAGACCCATTCAAATTACTCCAAAACAGCCAAGTGGTCAGCCTGATGAGACCAAATGAA  960

Query  990   AACCCAGCTCACAAGAGTCAATTGGTGTGGATGGCATGCCACTCTGCTGCATTTGAAGAT  1049
             ||||||||||||||||||||||||||||||||||||||||||||||||||||||||||||
Sbjct  961   AACCCAGCTCACAAGAGTCAATTGGTGTGGATGGCATGCCACTCTGCTGCATTTGAAGAT  1020

Query  1050  TTAAGAGTATCAAGTTTCATAAGAGGAAAGAAAGTGATTCCAAGAGGAAAGCTTTCCACA  1109
             ||||||||||||||||||||||||||||||||||||||||||||||||||||||||||||
Sbjct  1021  TTAAGAGTATCAAGTTTCATAAGAGGAAAGAAAGTGATTCCAAGAGGAAAGCTTTCCACA  1080

Query  1110  AGAGGGGTCCAGATTGCTTCAAATGAGAATGTGGAAACCATGGACTCCAATACCCTGGAA  1169
             ||||||||||||||||||||||||||||||||||||||||||||||||||||||||||||
Sbjct  1081  AGAGGGGTCCAGATTGCTTCAAATGAGAATGTGGAAACCATGGACTCCAATACCCTGGAA  1140
```

```
Query  1170  CTGAGAAGCAGATACTGGGCCATAAGGACCAGGAGTGGAGGAAATACCAATCAACAAAAG  1229
             ||||||||||||||||||||||||||||||||||||||||||||||||||||||||||||
Sbjct  1141  CTGAGAAGCAGATACTGGGCCATAAGGACCAGGAGTGGAGGAAATACCAATCAACAAAAG  1200

Query  1230  GCATCCGCAGGCCAGATCAGTGTGCAGCCTACATTCTCAGTGCAGCGGAATCTCCCTTTT  1289
             ||||||||||||||||||||||||||||||||||||||||||||||||||||||||||||
Sbjct  1201  GCATCCGCAGGCCAGATCAGTGTGCAGCCTACATTCTCAGTGCAGCGGAATCTCCCTTTT  1260

Query  1290  GAAAGAGCAACCGTTATGGCAGCATTCAGCGGGAACAATGAAGGACGGACATCCGACATG  1349
             ||||||||||||||||||||||||||||||||||||||||||||||||||||||||||||
Sbjct  1261  GAAAGAGCAACCGTTATGGCAGCATTCAGCGGGAACAATGAAGGACGGACATCCGACATG  1320

Query  1350  CGAACAGAAGTTATAAGAATGATGGAAAGTGCAAAGCCAGAAGATTTGTCCTTCCAGGGG  1409
             ||||||||||||||||||||||||||||||||||||||||||||||||||||||||||||
Sbjct  1321  CGAACAGAAGTTATAAGAATGATGGAAAGTGCAAAGCCAGAAGATTTGTCCTTCCAGGGG  1380

Query  1410  CGGGGAGTCTTCGAGCTCTCGGACGAAAAGGCAACGAACCCGATCGTGCCTTCCTTTGAC  1469
             ||||||||||||||||||||||||||||||||||||||||||||||||||||||||||||
Sbjct  1381  CGGGGAGTCTTCGAGCTCTCGGACGAAAAGGCAACGAACCCGATCGTGCCTTCCTTTGAC  1440

Query  1470  ATGAGTAATGAAGGGTCTTATTTCTTCGGAGACAATGCAGAGGAGTATGACAGTT  1524
             |||||||||||||||||||||||||||||||||||||||||||||||||||||||
Sbjct  1441  ATGAGTAATGAAGGGTCTTATTTCTTCGGAGACAATGCAGAGGAGTATGACAGTT  1495
```

6. Neuraminidase (NA)

```
Identities = 1400/1400 (100%), Gaps = 0/1400 (0%)

Query  10    ACCAAAAGATAATAACCATTGGTTCGGTCTGTATGACAATTGGAATGGCTAACTTAATAT  69
             ||||||||||||||||||||||||||||||||||||||||||||||||||||||||||||
Sbjct  11    ACCAAAAGATAATAACCATTGGTTCGGTCTGTATGACAATTGGAATGGCTAACTTAATAT  70

Query  70    TACAAATTGGAAACATAATCTCAATATGGATTAGCCACTCAATTCAACTTGGGAATCAAA  129
             ||||||||||||||||||||||||||||||||||||||||||||||||||||||||||||
Sbjct  71    TACAAATTGGAAACATAATCTCAATATGGATTAGCCACTCAATTCAACTTGGGAATCAAA  130

Query  130   ATCAGATTGAAACATGCAATCAAAGCGTCATTACTTATGAAAACAACACTTGGGTAAATC  189
             ||||||||||||||||||||||||||||||||||||||||||||||||||||||||||||
Sbjct  131   ATCAGATTGAAACATGCAATCAAAGCGTCATTACTTATGAAAACAACACTTGGGTAAATC  190

Query  190   AGACATATGTTAACATCAGCAACACCAACTTTGCTGCTGGACAGTCAGTGGTTTCCGTGA  249
             ||||||||||||||||||||||||||||||||||||||||||||||||||||||||||||
Sbjct  191   AGACATATGTTAACATCAGCAACACCAACTTTGCTGCTGGACAGTCAGTGGTTTCCGTGA  250

Query  250   AATTAGCGGGCAATTCCTCTCTCTGCCCTGTTAGTGGATGGGCTATATACAGTAAAGACA  309
             ||||||||||||||||||||||||||||||||||||||||||||||||||||||||||||
Sbjct  251   AATTAGCGGGCAATTCCTCTCTCTGCCCTGTTAGTGGATGGGCTATATACAGTAAAGACA  310

Query  310   ACAGTGTAAGAATCGGTTCCAAGGGGGATGTGTTTGTCATAAGGGAACCATTCATATCAT  369
             ||||||||||||||||||||||||||||||||||||||||||||||||||||||||||||
Sbjct  311   ACAGTGTAAGAATCGGTTCCAAGGGGGATGTGTTTGTCATAAGGGAACCATTCATATCAT  370

Query  370   GCTCCCCCTTGGAATGCAGAACCTTCTTCTTGACTCAAGGGGCCTTGCTAAATGACAAAC  429
             ||||||||||||||||||||||||||||||||||||||||||||||||||||||||||||
Sbjct  371   GCTCCCCCTTGGAATGCAGAACCTTCTTCTTGACTCAAGGGGCCTTGCTAAATGACAAAC  430

Query  430   ATTCCAATGGAACCATTAAAGACAGGAGCCCATATCGAACCCTAATGAGCTGTCCTATTG  489
             ||||||||||||||||||||||||||||||||||||||||||||||||||||||||||||
Sbjct  431   ATTCCAATGGAACCATTAAAGACAGGAGCCCATATCGAACCCTAATGAGCTGTCCTATTG  490

Query  490   GTGAAGTTCCCTCTCCATACAACTCAAGATTTGAGTCAGTCGCTTGGTCAGCAAGTGCTT  549
             ||||||||||||||||||||||||||||||||||||||||||||||||||||||||||||
Sbjct  491   GTGAAGTTCCCTCTCCATACAACTCAAGATTTGAGTCAGTCGCTTGGTCAGCAAGTGCTT  550

Query  550   GTCATGATGGCATCAATTGGCTAACAATTGGAATTTCTGGCCCAGACAATGGGGCAGTGG  609
             ||||||||||||||||||||||||||||||||||||||||||||||||||||||||||||
Sbjct  551   GTCATGATGGCATCAATTGGCTAACAATTGGAATTTCTGGCCCAGACAATGGGGCAGTGG  610

Query  610   CTGTGTTAAAGTACAACGGCATAATAACAGACACTATCAAGAGTTGGAGAAACAATATAT  669
             ||||||||||||||||||||||||||||||||||||||||||||||||||||||||||||
Sbjct  611   CTGTGTTAAAGTACAACGGCATAATAACAGACACTATCAAGAGTTGGAGAAACAATATAT  670
```

```
Query  670   TGAGAACACAAGAGTCTGAATGTGCATGTGTAAATGGTTCTTGCTTTACTGTAATGACCG  729
             ||||||||||||||||||||||||||||||||||||||||||||||||||||||||||||
Sbjct  671   TGAGAACACAAGAGTCTGAATGTGCATGTGTAAATGGTTCTTGCTTTACTGTAATGACCG  730

Query  730   ATGGACCAAGTAATGGACAGGCCTCATACAAGATCTTCAGAATAGAAAAGGGAAAGATAG  789
             ||||||||||||||||||||||||||||||||||||||||||||||||||||||||||||
Sbjct  731   ATGGACCAAGTAATGGACAGGCCTCATACAAGATCTTCAGAATAGAAAAGGGAAAGATAG  790

Query  790   TCAAATCAGTCGAAATGAATGCCCCTAATTATCACTATGAGGAATGCTCCTGTTATCCTG  849
             ||||||||||||||||||||||||||||||||||||||||||||||||||||||||||||
Sbjct  791   TCAAATCAGTCGAAATGAATGCCCCTAATTATCACTATGAGGAATGCTCCTGTTATCCTG  850

Query  850   ATTCTAGTGAAATCACATGTGTGTGCAGGGATAACTGGCATGGCTCGAATCGACCGTGGG  909
             ||||||||||||||||||||||||||||||||||||||||||||||||||||||||||||
Sbjct  851   ATTCTAGTGAAATCACATGTGTGTGCAGGGATAACTGGCATGGCTCGAATCGACCGTGGG  910

Query  910   TGTCTTTCAACCAGAATCTGGAATATCAGATAGGATACATATGCAGTGGGATTTTCGGAG  969
             ||||||||||||||||||||||||||||||||||||||||||||||||||||||||||||
Sbjct  911   TGTCTTTCAACCAGAATCTGGAATATCAGATAGGATACATATGCAGTGGGATTTTCGGAG  970

Query  970   ACAATCCACGCCCTAATGATAAGACAGGCAGTTGTGGTCCAGTATCGTCTAATGGAGCAA  1029
             ||||||||||||||||||||||||||||||||||||||||||||||||||||||||||||
Sbjct  971   ACAATCCACGCCCTAATGATAAGACAGGCAGTTGTGGTCCAGTATCGTCTAATGGAGCAA  1030

Query  1030  ATGGAGTAAAAGGGTTTTCATTCAAATACGGCAATGGTGTTTGGATAGGGAGAACTAAAA  1089
             ||||||||||||||||||||||||||||||||||||||||||||||||||||||||||||
Sbjct  1031  ATGGAGTAAAAGGGTTTTCATTCAAATACGGCAATGGTGTTTGGATAGGGAGAACTAAAA  1090

Query  1090  GCATTAGTTCAAGAAACGGTTTTGAGATGATTTGGGATCCGAACGGATGGACTGGGACAG  1149
             ||||||||||||||||||||||||||||||||||||||||||||||||||||||||||||
Sbjct  1091  GCATTAGTTCAAGAAACGGTTTTGAGATGATTTGGGATCCGAACGGATGGACTGGGACAG  1150

Query  1150  ACAATAACTTCTCAATAAAGCAAGATATCGTAGGAATAAATGAGTGGTCAGGATATAGCG  1209
             ||||||||||||||||||||||||||||||||||||||||||||||||||||||||||||
Sbjct  1151  ACAATAACTTCTCAATAAAGCAAGATATCGTAGGAATAAATGAGTGGTCAGGATATAGCG  1210

Query  1210  GGAGTTTTGTTCAGCATCCAGAACTAACAGGGCTGGATTGTATAAGACCTTGCTTCTGGG  1269
             ||||||||||||||||||||||||||||||||||||||||||||||||||||||||||||
Sbjct  1211  GGAGTTTTGTTCAGCATCCAGAACTAACAGGGCTGGATTGTATAAGACCTTGCTTCTGGG  1270

Query  1270  TTGAACTAATCAGAGGGCGACCCAAAGAGAACACAATCTGGACTAGCGGGAGCAGCATAT  1329
             ||||||||||||||||||||||||||||||||||||||||||||||||||||||||||||
Sbjct  1271  TTGAACTAATCAGAGGGCGACCCAAAGAGAACACAATCTGGACTAGCGGGAGCAGCATAT  1330

Query  1330  CCTTTTGTGGTGTAAACAGTGACACTGTGGGTTGGTCTTGGCCAGACGGTGCTGAGTTGC  1389
             ||||||||||||||||||||||||||||||||||||||||||||||||||||||||||||
Sbjct  1331  CCTTTTGTGGTGTAAACAGTGACACTGTGGGTTGGTCTTGGCCAGACGGTGCTGAGTTGC  1390

Query  1390  CATTTACCATTGACAAGTAA  1409
             ||||||||||||||||||||
Sbjct  1391  CATTTACCATTGACAAGTAA  1410
```

7. Matrix (M)

```
Identities = 855/855 (100%), Gaps = 0/855 (0%)

Query  26   ATGGACTCCAACACCATGTCAAGCTTTCAGGTAGACTGTTTCCTTTGGCATATCCGCAAG  85
            ||||||||||||||||||||||||||||||||||||||||||||||||||||||||||||
Sbjct  1    ATGGACTCCAACACCATGTCAAGCTTTCAGGTAGACTGTTTCCTTTGGCATATCCGCAAG  60

Query  86   CGATTTGCAGACAATGGATTGGGTGATGCCCCATTCCTTGATCGGCTCCGCCGAGATCAA  145
            ||||||||||||||||||||||||||||||||||||||||||||||||||||||||||||
Sbjct  61   CGATTTGCAGACAATGGATTGGGTGATGCCCCATTCCTTGATCGGCTCCGCCGAGATCAA  120

Query  146  AAGTCCTTAAAAGGAAGAGGCAACACCCTTGGCCTCGATATCGAAACAGCCACTCTTGTT  205
            ||||||||||||||||||||||||||||||||||||||||||||||||||||||||||||
Sbjct  121  AAGTCCTTAAAAGGAAGAGGCAACACCCTTGGCCTCGATATCGAAACAGCCACTCTTGTT  180

Query  206  GGGAAACAAATCGTGGAATGGATCTTGAAAGAGGAATCCAGCGAGACACTTAGAATGACA  265
            ||||||||||||||||||||||||||||||||||||||||||||||||||||||||||||
```

```
Sbjct  181  GGGAAACAAATCGTGGAATGGATCTTGAAAGAGGAATCCAGCGAGACACTTAGAATGACA  240

Query  266  ATTGCATCTGTACCTACTTCGCGCTACCTTTCTGACATGACCCTCGAGGAAATGTCACGA  325
            ||||||||||||||||||||||||||||||||||||||||||||||||||||||||||||
Sbjct  241  ATTGCATCTGTACCTACTTCGCGCTACCTTTCTGACATGACCCTCGAGGAAATGTCACGA  300

Query  326  GACTGGTTCATGCTCATGCCTAGGCAAAAGATAATAGGCCCTCTTTGCGTGCGATTGGAC  385
            ||||||||||||||||||||||||||||||||||||||||||||||||||||||||||||
Sbjct  301  GACTGGTTCATGCTCATGCCTAGGCAAAAGATAATAGGCCCTCTTTGCGTGCGATTGGAC  360

Query  386  CAGGCGATCATGGAAAAGAACATAGTACTGAAAGCGAACTTCAGTGTAATCTTTAACCGA  445
            ||||||||||||||||||||||||||||||||||||||||||||||||||||||||||||
Sbjct  361  CAGGCGATCATGGAAAAGAACATAGTACTGAAAGCGAACTTCAGTGTAATCTTTAACCGA  420

Query  446  TTAGAGACCTTGATACTACTAAGGGCTTTCACTGAGGAGGGAGCAATAGTTGGAGAAATT  505
            ||||||||||||||||||||||||||||||||||||||||||||||||||||||||||||
Sbjct  421  TTAGAGACCTTGATACTACTAAGGGCTTTCACTGAGGAGGGAGCAATAGTTGGAGAAATT  480

Query  506  TCACCATTACCTTCTCTTCCAGGACATACTTATGAGGATGTCAAAAATGCAGTTGGGGTC  565
            ||||||||||||||||||||||||||||||||||||||||||||||||||||||||||||
Sbjct  481  TCACCATTACCTTCTCTTCCAGGACATACTTATGAGGATGTCAAAAATGCAGTTGGGGTC  540

Query  566  CTCATCGGAGGACTTGAATGGAATGGTAACACGGTTCGAGTCTCTGAAAATATACAGAGA  625
            ||||||||||||||||||||||||||||||||||||||||||||||||||||||||||||
Sbjct  541  CTCATCGGAGGACTTGAATGGAATGGTAACACGGTTCGAGTCTCTGAAAATATACAGAGA  600

Query  626  TTCGCTTGGAGAAACTGTGATGAGAATGGGAGACCTTCACTACCTCCAGAGCAGAAATGA  685
            ||||||||||||||||||||||||||||||||||||||||||||||||||||||||||||
Sbjct  601  TTCGCTTGGAGAAACTGTGATGAGAATGGGAGACCTTCACTACCTCCAGAGCAGAAATGA  660

Query  686  AAAGTGGCGAGAGCAATTGGGACAGAAATTTGAGGAAATAAGGTGGTTAATTGAAGAAAT  745
            ||||||||||||||||||||||||||||||||||||||||||||||||||||||||||||
Sbjct  661  AAAGTGGCGAGAGCAATTGGGACAGAAATTTGAGGAAATAAGGTGGTTAATTGAAGAAAT  720

Query  746  GCGGCACAGATTGAAAGCGACAGAGAATAGTTTCGAACAAATAACATTTATGCAAGCCTT  805
            ||||||||||||||||||||||||||||||||||||||||||||||||||||||||||||
Sbjct  721  GCGGCACAGATTGAAAGCGACAGAGAATAGTTTCGAACAAATAACATTTATGCAAGCCTT  780

Query  806  ACAACTACTGCTTGAAGTAGAACAAGAGATAAGAGCTTTCTCGTTTCAGCTTATTTAATG  865
            ||||||||||||||||||||||||||||||||||||||||||||||||||||||||||||
Sbjct  781  ACAACTACTGCTTGAAGTAGAACAAGAGATAAGAGCTTTCTCGTTTCAGCTTATTTAATG  840

Query  866  ATAAAAAACACCCTT  880
            |||||||||||||||
Sbjct  841  ATAAAAAACACCCTT  855
```

8. Nonstructural protein (NS)

```
Identities = 855/855 (100%), Gaps = 0/855 (0%)

Query  26   ATGGACTCCAACACCATGTCAAGCTTTCAGGTAGACTGTTTCCTTTGGCATATCCGCAAG  85
            ||||||||||||||||||||||||||||||||||||||||||||||||||||||||||||
Sbjct  1    ATGGACTCCAACACCATGTCAAGCTTTCAGGTAGACTGTTTCCTTTGGCATATCCGCAAG  60

Query  86   CGATTTGCAGACAATGGATTGGGTGATGCCCCATTCCTTGATCGGCTCCGCCGAGATCAA  145
            ||||||||||||||||||||||||||||||||||||||||||||||||||||||||||||
Sbjct  61   CGATTTGCAGACAATGGATTGGGTGATGCCCCATTCCTTGATCGGCTCCGCCGAGATCAA  120

Query  146  AAGTCCTTAAAAGGAAGAGGCAACACCCTTGGCCTCGATATCGAAACAGCCACTCTTGTT  205
            ||||||||||||||||||||||||||||||||||||||||||||||||||||||||||||
Sbjct  121  AAGTCCTTAAAAGGAAGAGGCAACACCCTTGGCCTCGATATCGAAACAGCCACTCTTGTT  180

Query  206  GGGAAACAAATCGTGGAATGGATCTTGAAAGAGGAATCCAGCGAGACACTTAGAATGACA  265
            ||||||||||||||||||||||||||||||||||||||||||||||||||||||||||||
Sbjct  181  GGGAAACAAATCGTGGAATGGATCTTGAAAGAGGAATCCAGCGAGACACTTAGAATGACA  240

Query  266  ATTGCATCTGTACCTACTTCGCGCTACCTTTCTGACATGACCCTCGAGGAAATGTCACGA  325
            ||||||||||||||||||||||||||||||||||||||||||||||||||||||||||||
Sbjct  241  ATTGCATCTGTACCTACTTCGCGCTACCTTTCTGACATGACCCTCGAGGAAATGTCACGA  300
```

```
Query  326  GACTGGTTCATGCTCATGCCTAGGCAAAAGATAATAGGCCCTCTTTGCGTGCGATTGGAC  385
            ||||||||||||||||||||||||||||||||||||||||||||||||||||||||||||
Sbjct  301  GACTGGTTCATGCTCATGCCTAGGCAAAAGATAATAGGCCCTCTTTGCGTGCGATTGGAC  360

Query  386  CAGGCGATCATGGAAAAGAACATAGTACTGAAAGCGAACTTCAGTGTAATCTTTAACCGA  445
            ||||||||||||||||||||||||||||||||||||||||||||||||||||||||||||
Sbjct  361  CAGGCGATCATGGAAAAGAACATAGTACTGAAAGCGAACTTCAGTGTAATCTTTAACCGA  420

Query  446  TTAGAGACCTTGATACTACTAAGGGCTTTCACTGAGGAGGGAGCAATAGTTGGAGAAATT  505
            ||||||||||||||||||||||||||||||||||||||||||||||||||||||||||||
Sbjct  421  TTAGAGACCTTGATACTACTAAGGGCTTTCACTGAGGAGGGAGCAATAGTTGGAGAAATT  480

Query  506  TCACCATTACCTTCTCTTCCAGGACATACTTATGAGGATGTCAAAAATGCAGTTGGGGTC  565
            ||||||||||||||||||||||||||||||||||||||||||||||||||||||||||||
Sbjct  481  TCACCATTACCTTCTCTTCCAGGACATACTTATGAGGATGTCAAAAATGCAGTTGGGGTC  540

Query  566  CTCATCGGAGGACTTGAATGGAATGGTAACACGGTTCGAGTCTCTGAAAATATACAGAGA  625
            ||||||||||||||||||||||||||||||||||||||||||||||||||||||||||||
Sbjct  541  CTCATCGGAGGACTTGAATGGAATGGTAACACGGTTCGAGTCTCTGAAAATATACAGAGA  600

Query  626  TTCGCTTGGAGAAACTGTGATGAGAATGGGAGACCTTCACTACCTCCAGAGCAGAAATGA  685
            ||||||||||||||||||||||||||||||||||||||||||||||||||||||||||||
Sbjct  601  TTCGCTTGGAGAAACTGTGATGAGAATGGGAGACCTTCACTACCTCCAGAGCAGAAATGA  660

Query  686  AAAGTGGCGAGAGCAATTGGGACAGAAATTTGAGGAAATAAGGTGGTTAATTGAAGAAAT  745
            ||||||||||||||||||||||||||||||||||||||||||||||||||||||||||||
Sbjct  661  AAAGTGGCGAGAGCAATTGGGACAGAAATTTGAGGAAATAAGGTGGTTAATTGAAGAAAT  720

Query  746  GCGGCACAGATTGAAAGCGACAGAGAATAGTTTCGAACAAATAACATTTATGCAAGCCTT  805
            ||||||||||||||||||||||||||||||||||||||||||||||||||||||||||||
Sbjct  721  GCGGCACAGATTGAAAGCGACAGAGAATAGTTTCGAACAAATAACATTTATGCAAGCCTT  780

Query  806  ACAACTACTGCTTGAAGTAGAACAAGAGATAAGAGCTTTCTCGTTTCAGCTTATTTAATG  865
            ||||||||||||||||||||||||||||||||||||||||||||||||||||||||||||
Sbjct  781  ACAACTACTGCTTGAAGTAGAACAAGAGATAAGAGCTTTCTCGTTTCAGCTTATTTAATG  840

Query  866  ATAAAAAACACCCTT  880
            |||||||||||||||
Sbjct  841  ATAAAAAACACCCTT  855
```
