# Supplementary material for: IAVCP (Influenza A Virus Consensus and Phylogeny): Automatic Identification of the Genomic Sequence of the Influenza A Virus from High-Throughput Sequencing Data
Source: Viruses. 2024 May 29;16(6):873. doi: 10.3390/v16060873 (PMC11209090; doi:10.3390/v16060873)
Supplement: Supplementary file 1 [file viruses-16-00873-s001.zip › viruses-2945568 suppl revised/viruses-2945568-supplementary.html]

Snakemake Report


Loading Snakemake Report...

Please enable Javascript in your browser to see this report.
